# Phenotypic and genomic analyses of bacteriophages targeting environmental and clinical CS3-expressing enterotoxigenic *Escherichia coli* (ETEC) strains

**DOI:** 10.1371/journal.pone.0209357

**Published:** 2018-12-20

**Authors:** Sajib Chakraborty, Astrid von Mentzer, Yasmin Ara Begum, Mehnaz Manzur, Mahmudul Hasan, Amar N. Ghosh, M. Anwar Hossain, Andrew Camilli, Firdausi Qadri

**Affiliations:** 1 Department of Biochemistry and Molecular Biology, University of Dhaka, Dhaka, Bangladesh; 2 icddr,b (International Centre for Diarrhoeal Disease Research, Bangladesh), Mohakhali, Dhaka, Bangladesh; 3 Department of Microbiology and Immunology, Sahlgrenska Academy, University of Gothenburg, Gothenburg, Sweden; 4 National Institute of Cholera and Enteric Diseases, Kolkata, India; 5 Department of Molecular Biology and Microbiology, and Howard Hughes Medical Institute, Tufts University School of Medicine, Boston, MA, United States of America; Icahn School of Medicine at Mount Sinai, UNITED STATES

## Abstract

Diarrhea due to infection of enterotoxigenic *Escherichia coli* (ETEC) is of great concern in several low and middle-income countries. ETEC infection is considered to be the most common cause of diarrhea in Bangladesh and is mainly spread through contaminated water and food. ETEC pathogenesis is mediated by the expression of enterotoxins and colonization factors (CFs) that target the intestinal mucosa. ETEC can survive for extended time periods in water, where they are likely to be attacked by bacteriophages. Antibiotic resistance is common amongst enteric pathogens and therefore is the use of bacteriophages (phage) as a therapeutic tool an interesting approach. This study was designed to identify novel phages that specifically target ETEC virulence factors. In total, 48 phages and 195 ETEC isolates were collected from water sources and stool samples. Amongst the identified ETEC specific phages, an enterobacteria phage T7, designated as IMM-002, showed a significant specificity towards colonization factor CS3-expressing ETEC isolates. Antibody-blocking and phage-neutralization assays revealed that CS3 is used as a host receptor for the IMM-002 phage. The bacterial CRISPR-Cas (Clustered Regularly Interspaced Short Palindromic Repeats-CRISPR-associated) defence mechanism can invoke immunity against phages. Genomic analyses coupled with plaque assay experiments indicate that the ETEC CRISPR-Cas system is involved in the resistance against the CS3-specific phage (IMM-002) and the previously identified CS7-specific phage (IMM-001). As environmental water serves as a reservoir for ETEC, it is important to search for new antimicrobial agents such as phages in environmental water as well as the human gut. A better understanding of how the interplay between ETEC-specific phages and ETEC isolates affects the ETEC diversity, both in environmental ecosystems and within the host, is important for the development of new treatments for ETEC infections.

## Introduction

Enterotoxigenic *Escherichia coli* (ETEC) is one of the main causes of childhood-diarrhea in low and middle-income countries and in travelers to endemic areas [1]. ETEC is defined by their ability to produce enterotoxins; heat-labile toxin (LT) and/or heat-stable toxin (ST) (including two subtypes, STh and STp). Colonization factors (CF) are outer membrane fimbrial, fibrillar or afimbrial proteins, which mediate adherence to the small intestinal mucosa. To date, over 25 different CFs, have been described for ETEC infecting humans [2,3]. The most prevalent CFs are present in 50–80% of all clinical ETEC isolates, these include CFA/1, CS1, CS2, CS3, CS4, CS5, CS6, CS7, CS14, CS17, and CS21 [4].

ETEC-mediated diarrhea can typically be initiated through the intake of contaminated food or water. The ability of ETEC strains to survive for months in water such as rivers, ponds and lake without losing the ability to express the virulence factors indicates that ETEC may utilize environmental water both as an ecological niche and as a route of transmission [5].

Bacteriophage (phage) predation of other diarrheagenic bacteria, specifically *Vibrio cholerae*, has been reported to influence the seasonal epidemics of cholera in Bangladesh [6]. Unlike *V*. *cholerae* the interaction between ETEC and phages is poorly understood. Studies have shown that *E*. *coli* phages can be specific to *E*. *coli* serogroups [7,8] or to the capsular polysaccharide antigen of *E*. *coli* [9]. To date, only one phage that specifically targets ETEC expressing the CF CS7 has been reported [10]. It is of interest to investigate if there are additional CFs that could be targeted by phages to gain further insight into how the interplay between ETEC and phages could affect ETEC diversity.

The Clustered Regularly Interspaced Short Palindromic Repeat (CRISPR) arrays and CRISPR associated (Cas) genes constitute CRISPR-Cas systems that protect bacteria from phage infections and restricts foreign DNA to be incorporated into the bacterial genome. The interference mechanism of CRISPR-Cas has emerged as an ideal system to study predator-prey, in particular, host-phage interactions [11]. The adaptability of the CRISPR system allows the bacteria to cope with the dynamic nature of the arms race that is constantly ongoing between phages and their host bacteria [12]. Many bacterial and archaeal genomes have acquired one or multiple CRISPR-Cas loci through horizontal gene transfer (HGT), including new spacers which are complementary to genomes of invading phages [13].The acquisition of new spacers are dependent on the Cas1 and Cas2 proteins [14,15]. The spacer sequences of CRISPR arrays reflect the past invasion encounters that a particular bacterium has been exposed to. Phage DNA sequences that share complementarity to already existing spacers in the bacterial genome are referred to as protospacer sequences [16]. Invading protospacer sequences must harbor a protospacer adjacent motif (PAM) to be successfully targeted by the bacterial spacer sequences[16]. PAM plays an integral role for the interference mechanism since mutations in the PAM sequence within the invading DNA prevent cleavage of the protospacer [17,18]. The CRIPSR-Cas systems are divided into five distinct types depending on the *cas* genes encoding the effector modules. These CRISPR-Cas types are further divided into different subtypes according to the architectures of the genomic loci of the CRISPR-Cas systems [19]. It has recently been shown that in Type I subtype E (Type I-E) CRISPR-Cas systems the complementarity between the spacer and protospacer is only strict for a seed region consisting of seven-nucleotides adjacent to the PAM [20]. The CRISPR-Cas system has not been as extensively investigated in pathogenic *E*. *coli*, such as ETEC, as it has in laboratory *E*. *coli* strains. Recently García-Gutiérrez *et al*. identified CRISPR-Cas systems in pathogenic *E*. *coli* including endometrial (EnPEC) and uropathogenic *E*. *coli* (UPEC) [21]. However, the study did not report on the aspect of CRISPR-Cas mediated host-phage interactions between pathogenic *E*. *coli* strains and phages. One challenge in studying the dynamic interaction between *E*. *coli* strains and their predatory phages is the lack of phage genomes sequences. Only a small percentage of identified *E*. *coli* Type I-E CRISPR spacers that are publicly available actually match phage-derived protospacer sequences [22,23].

The aim of this study was to identify phages targeting specific virulence factor expressed by ETEC from environmental water sources and clinical stool samples in Bangladesh. We have isolated and characterized a new phage, designated IMM-002, which showed remarkable specificity towards ETEC strains expressing colonization factor CS3. Next, we characterized he newly isolated IMM-002 phage and the previously isolated CS7-specific IMM-001 phage using molecular and genomic analyses. Furthermore, we have described the CRISPR-Cas systems identified in CS3 and CS7-expressing ETEC strains to better understand the interaction between ETEC and their predatory phages in both water and clinical stool samples.

## Materials and methods

### Isolation of ETEC strains and phages from environmental water

Environmental water samples from three sewage-plants and five different ponds around Dhaka city were collected in 500 milliliters (ml) sterile bottles. To isolate ETEC strains from the pond and water from sewage-plants, 150 ml aliquot of each water sample was filtered through a 0.22 μm Millipore nitrocellulose membrane filter paper. The material collected on the filter was washed with 1 ml of MacConkey broth (Difco, Becton Dickinson, USA), and inoculated into 9 ml MacConkey broth at 37°C for 4 hours. Subsequently, 100 μl of these cultures were spread onto MacConkey agar plates and incubated overnight at 37°C. Lactose-fermenting *E*. *coli* were tested for the presence of the enterotoxins LT (*eltAB*), STh (*estA*), and STp*(estB*) by multiplex PCR amplification method using gene-specific primers, as described by Sjoling *el al*. [24]. Briefly, a total of 13 μl of the master mix was added to each tube. Then 2 μl of previously prepared template DNA was added to both tubes in a total volume of 20 μl. PCR reaction was performed with a PTC-200 thermal cycler (Peltier Thermal Cycler, MJ Research) for 45 cycles. The PCR products were separated in a 2% agarose gel, stained by ethidium bromide and visualized under UV-light.

Phages were isolated from the same water samples from which ETEC isolates were screened for. Ten ml of each water sample was sterilized with one ml of chloroform using a slightly modified procedure [25]. Chloroform treated water was filtered through a 0.22 μm membrane filter. Twenty microliters (μl) of filtered water samples were spotted onto well-dried lawns of indicator ETEC strains in Heart Infusion agar (HIA) plates. As indicator strains 13 different ETEC strains expressing a specific CF or combination of CFs were used: 258909–3 (CFA/I), E1392-75 (CS1 plus CS3), 278485 (CS2 plus CS3), 3023 (CS3),E11881/9 (CS4 plus CS6), VM 75688 (CS5 plus CS6), BCE#243 (CS6), T-2255272 (CS7), E34420A (CS8 or CFA/III), 350CIA (CS12 or PCFO159), E7474A (CS14 or PCFO166), E-20738A (CS17) and E9034A (CS21).The plates were incubated overnight at 37°C, and plaques were observed on the following day. The phages which showed higher stability were selected for further studies. Each of the selected phages was purified by three successive picks of single plaques by the soft agar layer method [25].

### Isolation and characterization of phage and ETEC strains from clinical specimens

For isolation of ETEC strains from clinical samples, stools from 200 patients (including 150 children<5 years of age and 50 adults) suffering from acute diarrhea were plated onto MacConkey agar plates and incubated at 37°C overnight. The detection of the ETEC strains in stool samples was conducted by identifying the presence of the enterotoxins LT (*eltAB*), STh (*estA*), and STp (*estB*) by multiplex PCR (as described above).

To isolate phages from the clinical samples, same stool specimens were used that were found to be ETEC positive. Stool samples were sterilized with chloroform and centrifuged at 3000 x *g* for 30 minutes. Chloroform treated water was filtered through a 0.22 μm membrane filter. One ml of the supernatant was added to the five ml early log phase growth of the indicator strains (10^8^ CFU) (as described above) in Heart Infusion broth (HIB) (Becton Dickinson, USA). The phage purification step was performed as described above.

### Phenotypic detection of ETEC toxin and CFs

The expression of LT and ST was tested using GM1 enzyme-linked immunosorbent assay (ELISA) methods [26]. Six lactose-fermenting *E*. *coli* colonies from MacConkey agar were inoculated on GM1-coated microtiter plates containing Luria-Bertani (LB) broth for 18 hours. The supernatant was tested for ST using an inhibition ELISA procedure [27] and for LT by using an anti-LT monoclonal antibody [26]. ETEC strains were cultured on CFA agar plates containing 0.15% bile [28] and tested for the expression of CFA/I, CS1 to CS8, CS12, CS14, CS17, CS19, and CS21 by a monoclonal antibody-based dot-blot assay [3]. Reference ETEC strains were used as positive controls [26]. The monoclonal antibodies against CFs except anti-CS21 were produced at the Department of Microbiology and Immunology at the University of Gothenburg and provided by Professor Ann-Mari Svennerholm. The CS21 antibody was produced at the immunology laboratory at icddr,b [29].

### Serotyping of ETEC strains

ETEC strains were serotyped with standard techniques [30] using eight commercially available polyvalent and 43 monovalent antisera (Denka Seiken, Japan) for specific somatic (O) antigens. ETEC colonies were sub-cultured on 5% sheep blood agar plates and serological reactions were performed using a slide agglutination test.

### Screening of the selected phages against ETEC strains

To determine the phage specificity for ETEC virulence factors, spotting of phages on lawns of each of the reference ETEC strains was performed. Single colonies of the reference ETEC strains were inoculated into 3 ml of HIB and incubated at 37°C for 4 hours. The fresh culture was then spread on a well-dried HIA plate. The plate was then dried at 37°C for 1 hour and appropriate dilutions of 10 phages were then spotted on the plate and incubated at 37°C for 18–20 hours. Broth without phage was employed as a negative control on each plate. The presence or absence of phage lysis was recorded for each phage against each reference ETEC strain tested.

### Antibody blocking assay by anti-CS3 monoclonal antibodies

To test the inhibition of phage infection, 100 μl of 10^−1^ to 10^−5^ dilutions of specific anti-CF monoclonal antibody (anti-CS1, anti-CS2, and anti-CS3) in HIB broth were incubated for 3 hours at room temperature with ETEC strains 278485–2 and E1392-75; 10^8^ CFU in 100 μl of HIB broth. The HIB medium without antibody was used as a control. The recipient cells mixed with antibodies were plated and incubated at 37°C for 1 hour prior to the addition of the 20 μl of phage solution (10^7^ PFU/ml in HIB). Plaques were counted in the following day after an overnight incubation period at 37°C. Here we used two ETEC strains 278485–2 (CS2+CS3) and E1392-75 (CS1+CS3) for the assay and the efficiency of plating (EOP) (%) of IMM-002 in these two strains with or without antibody is measured and an average was taken.

### Phage neutralization assay by purified CF fimbrial protein

The phage-neutralizing capacity of purified CFs (CS1, CS2, and CS3) was determined by incubating phage with various concentrations of the purified CFs supplied by the Department of Microbiology and Immunology at the University of Gothenburg. A total of 100 μl of purified CFs at different concentrations, ranging from 100 μg/ml to 500 μg/ml, were incubated with phage particles (10^7^ PFU in 100 μl of HIB broth) for 2 hours. The phage-CF mixtures (20 μl) were spotted on the lawn of reference ETEC strains (278485–2 and E1392-75) expressing specific CF(s) in the HIA plate. The EOP (%) was calculated by taking the average of the results obtained by using two reference ETEC strains independently. Plaques were counted after incubation at 37°C overnight.

### Electron microscopy of IMM-002 phage

Morphological studies on bacteriophage were carried out by negative staining with 2% uranyl acetate and examined under a Philips transmission electron microscope (Model 420T) [31].

### Genome sequencing and analysis of IMM-002 and IMM-001 phages

The phage isolates (IMM-001 and IMM-002) were sequenced using the Homopolymer tail-mediated ligation PCR (HTML-PCR) [32] on an Illumina HiSeq2500 instrument. A single-end, 50 cycle run was performed, which yielded 5,508,206 and 3,859,974 high-quality reads for CS3 and CS7, respectively. The genomes were de novo assembled using CLC Genomics Workbench software (Qiagen, Inc) with default settings. Single contigs with average read coverages of 136 and 117 for IMM-002 and IMM-001, respectively, were obtained. Since the phage genomes are unlikely to be a covalently closed circle within phage virions, we have chosen to leave them in linear form. Contigs with read coverages less than 10 were removed as these were shown to be trace DNA contamination from the *E*. *coli* host used to generate the high titer phage stocks used for sequencing. Genomic libraries were generated for IMM-002 and IMM-001 phage as described by Lazinski *et al*. [32]. Open reading frames (ORFs) of the sequenced phages were identified by the NCBI ORF finder (https://www.ncbi.nlm.nih.gov/orffinder/l). Genome comparison of the annotated IMM-002 and the two most closely related phages was performed using progressive MAUVE(v2.0) [33]. The phage genomes were submitted to GenBank with the following accession numbers: MF630921 (IMM-002) and MF630922 (IMM-001) (File S1).

### Identification and characterization of CRISPR arrays in CS3 and CS7-expressing ETEC strains

Whole genome sequences of 13 different CS3 (in combination with CS1, CS2 or CS21) and CS7-expressing ETEC strains were retrieved from NCBI genome database (See Supplementary S4 Table). ETEC genomes were analyzed using the CRISPR finder tool http://crispr.i2bc.paris-saclay.fr/Server/ [34] to identify potential CRISPR loci. CRISPR loci, as confirmed by the CRISPR finder, were taken into consideration and questionable CRISPR loci were discarded.

### Phylogenetic analysis

*“cas* operons” were identified using nucleotide BLAST (BLASTn) and the individual *cas* genes were extracted and annotated manually. The protein sequence of Cas1 from each CS3 positive ETEC strain was aligned by MUSCLE and a phylogenetic tree using the maximum likelihood algorithm in Seaview (v 4.5.4) was generated. The amino acid sequence comparisons of the Cas proteins with the reference proteins representing the two CRISPR-Cas I-E variants E1 (E24377A) and E2 (MG1655) were performed in Seaview (v 4.5.4) after using the built-in muscle aligner.

### Searching protospacer sequences in the phage genome

In order to identify and locate the protospacer sequences in the phage genome, BLASTn (http://blast.ncbi.nlm.nih.gov/) was utilized. The parameters were optimized to search for short nucleotide matches as described by Yaung*et al*. [35]. Briefly, a word size of 7 was set, optimized for short sequences. To identify the protospacer sequences the following criteria were chosen: no mismatch in the 5’ seed region consisting of 7 nucleotides and outside of the seed region, 10 mismatches were allowed. Furthermore, PAMs were also documented for a potential protospacer sequences.

### Identification of CRISPR targets

CRISPRTarget tool (http://bioanalysis.otago.ac.nz/CRISPRTarget/crispr_analysis.html) was used to identify the plasmid and phage targets for the CS3 and CS7 expressing ETEC CRISPR systems. The spacer sequences within the CRISPR arrays of CS3- and CS7-expressing ETEC strains were used to scan the target in Genbank-phage and Refseq-Plasmid databases. Among the targets reported by CRISPRTarget tool the phage and plasmid sequences that were associated with known Type I-E and Type I-F PAM were selected for further analysis.

### Plaque assay to determine the phage resistance capacity of the ETEC strains

To determine the efficiency of CRISPR-Cas interference of CS3 and CS7-expressing ETEC strains against IMM-002 and IMM-001 phage infection, respectively, plaque assays were performed by spotting phages on lawns of bacteria. In total, 10^8^ CFU ETEC strains in 100 μl of HIB broth were plated and incubated at 37°C for 1 hour prior to the addition of the 20 μl of phage solution (10^7^ PFU/ml in HIB). The EOP (%) was calculated (as mentioned above) in the following day after an overnight incubation period at 37°C to determine the percentage bacteria infected by phages. In total, eleven CS3-expressing and thirteen CS7-expressing ETEC strains were tested.

### Ethical statements

The collection of samples was carried out in accordance with guidelines and regulations of ethical guidelines for clinical research and ethical review committee, icddr,b (International Center for diarrheal disease research, Bangladesh). All experimental protocols were approved by the ethical review committee, icddr,b. Informed consent was obtained from the participants to collect the stool samples. Environmental water samples from different ponds around Dhaka city were collected by the field staff of icddr,b. Collection of water samples for research purpose is exempted from any regulatory and ethical control as per rule by the Department of Environment of the Bangladesh Government.

## Results and discussion

### Isolation of ETEC-specific phages and determination of phage specificity to ETEC colonization factors

**Fig 1:**
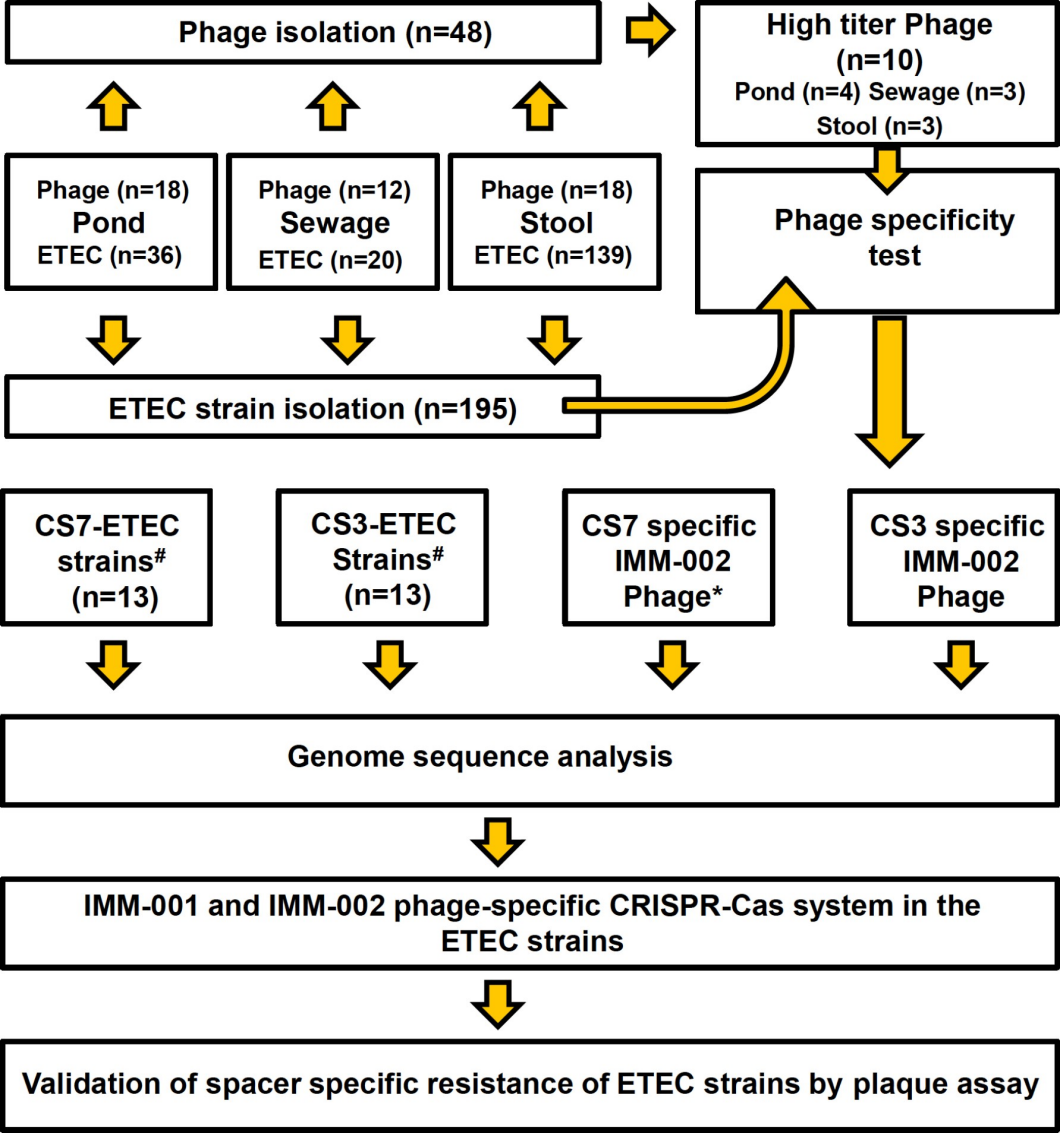
Schematic diagram representing the workflow including isolation, specificity determination, characterization and genome analysis of CF-specific ETEC phages and host ETEC strains. In total 48 phages and 195 ETEC isolates were collected from pond water, sewage-plant(s) and stool samples. ETEC strains were characterized by determining the colonization factor and toxin profile by genotypic and phenotypic methods, including PCR, ELISA and dot blot assays. Furthermore, the ETEC strains were subjected to serotyping. The specificity of each isolated phage was determined by plaque assays against the 195 characterized ETEC strains. One selected phage specific for ETEC expressing CS3 (IMM-002) was selected for further analyses. The IMM-002 phage (indicated by *) and the previously isolated phage IMM-001 [10], targeting ETEC expressing CS7, were whole genome sequenced. Apart from the 195 newly isolated ETEC strains, 23 publicly available genomes of ETEC strains expressing CS3 (n = 13) and CS7 (n = 13) were analyzed and further used for phage susceptibility via plaque assay.

Water samples from ponds and sewage-plants, as well as stool samples, were screened for phages and ETEC isolates. In total, 48 phages were isolated from pond water (n = 18), sewage-plants (n = 12) and stool samples (n = 18) (Fig 1). Based on the stability upon storage and high titer of the phages, ten phages (four from pond water, three from and sewage-plants and three from stool samples) were selected for further characterization (Table 1). A total of 195 ETEC strains with diverse toxin and CF profiles were isolated from the same pond water (n = 36), sewage-plants (n = 20) and stool samples (n = 139) from which the phages were isolated (Fig 1).

**Table 1:** List of ten phages isolated from pond, sewage and stool samples.

PhageID	Source	Propagating strains				Titer of the phage
		Strain ID.	Species	Toxin type	CFs	(PFU/ml)
PΦ9	Pond	VM75688	*E*. *coli*	LT+ST	CS5+CS6	3.7 x 10^7^
PΦ3	Pond	350CIA	*E*. *coli*	LT+ST	CS12	2.5 x 10^7^
PΦ18	Pond	258909	*E*. *coli*	LT+ST	CFA/I	2x 10^5^
PΦ15*	Pond	278485–2	*E*. *coli*	LT+ST	CS2+CS3	2.5 x 10^8^
SΦ2	Stool	E1392-75	*E*. *coli*	LT+ST	CS1+3	2 x 10^4^
SΦ9	Stool	E7476	*E*. *coli*	ST	CS14	1 x 10^5^
SΦ14	Stool	VM75688	*E*. *coli*	LT+ST	CS5+CS6	2.5 x 10^4^
EΦ2	Sewage	278485–2	*E*. *coli*	LT+ST	CS2+3	1 x 10^8^
EΦ4	Sewage	VM75688	*E*. *coli*	LT+ST	CS5+6	2.5 x 10^7^
EΦ9	Sewage	E7476	*E*. *coli*	ST	CS14	3.7 x 10^7^

*IMM-002 phage

Among the 195 ETEC strains, 31 and 83 strains expressed LT or ST alone, respectively, whereas the remaining 81 ETEC strains expressed both LT and ST, as confirmed by PCR and ELISA (Table 2). Eleven different CFs individually or in combination with others and 11 different O-antigens were identified by dot blot assays. Eight strains did not express any of the 13 CFs tested (Table 2). The 10 selected phages were tested for their specificity against the 195 ETEC strains. One phage, denoted as IMM-002, showed a significant specificity toward ETEC strains expressing CS3 alone or in combination with other CFs such as CS1, CS2 and CS21. The CS3-expessing ETEC strains were isolated from all three sources (ponds: n = 26, sewage-plants: n = 6, stool samples: n = 48). All the CS3 positive ETEC strains (n = 83) that were lysed by IMM-002 belonged to the O6 serogroup. However, CS17 expressing ETEC strains with the same O6 serotype as CS3 expressing ETEC strains were not susceptible to IMM-002. Furthermore, other common enteric bacteria were not susceptible to IMM-002 infection (S1 Table) indicating that the IMM-002 phage is ETEC specific.

**Table 2:** Screening of 10 phages against 195 ETEC strains.

No. of strains tested	No. of sensitive strains	CFs	O serogroup	Toxins		Phages tested									
				LT	ST	PΦ9	PΦ3	SΦ2	PΦ18	PΦ15*	SΦ9	EΦ2	EΦ4	SΦ14	EΦ9
25	25	CS2 + CS3	O6	**+**	**+**	**-**	**-**	**-**	**-**	**+**	**-**	**+**	**-**	**-**	**-**
23	23	CS2 + CS3	O6	**-**	**+**	**-**	**-**	**-**	**-**	**+**	**-**	**-**	**-**	**-**	**-**
12	12	CS1 + CS3	O6	**+**	**+**	**-**	**-**	**+**	**-**	**+**	**-**	**+**	**-**	**-**	**-**
10	10	CS1+CS3+CS21	O6	**+**	**+**	**-**	**-**	**-**	**-**	**+**	**-**	**-**	**-**	**-**	**-**
7	7	CS1+CS3+CS21	O6	**-**	**+**	**-**	**-**	**-**	**-**	**+**	**-**	**-**	**-**	**-**	**-**
6	6	CS3	O6	**-**	**+**	**-**	**-**	** **	**-**	**+**	**-**	**-**	**-**	**-**	**-**
15	15	CS5 + CS6	O115,O167,O12	**+**	**+**	**+**	**+**	**-**	**+**	**-**	**-**	**-**	**+**	**+**	**-**
7	6	CS5+CS6	O115	**-**	**+**	**+**	**+**	**-**	**+**	**-**	**-**	**-**	**+**	**+**	**-**
5	0	CS6	O167	**+**	**+**	**-**	**-**	**-**	**-**	**-**	**-**	**-**	**-**	**-**	**-**
4	1	CS6	O167,O19	**-**	**+**	**+**	**-**	**-**	**-**	**-**	**-**	**-**	**-**	**-**	**-**
17	0	CS7	O114	**+**	**-**	**-**	**-**	**-**	**-**	**-**	**-**	**-**	**-**	**-**	**-**
8	1	CS12	O159	**+**	**+**	**-**	**+**	**-**	**+**	**-**	**-**	**-**	**+**	**+**	**-**
6	1	CS12	O159	**+**	**-**	**-**	**+**	**-**	**+**	**-**	**-**	**-**	**+**	**+**	**-**
17	5	CS14	O166, NT	**-**	**+**	**-**	**+**	**-**	**+**	**-**	**+**	**-**	**+**	**+**	**+**
2	2	CS14	O166	**+**	**+**	**+**	**-**	**-**	**-**	**-**	**-**	**-**	**-**	**-**	**-**
5	0	CS17	O128,O6	**+**	**-**	**-**	**-**	**-**	**-**	**-**	**-**	**-**	**-**	**-**	**-**
1	0	CS17	O20	**-**	**+**	**-**	**-**	**-**	**-**	**-**	**-**	**-**	**-**	**-**	** **
9	1	CFA/I	O126	**-**	**+**	**-**	**-**	**-**	**+**	**-**	**+**	**-**	**-**	**-**	**+**
2	0	CFA/I	O126	**+**	**+**	**-**	**-**	**-**	**-**	**-**	**-**	**-**	**-**	**-**	**-**
3	0	CF-ve	NT	**+**	**-**	**-**	**-**	**-**	**-**	**-**	**-**	**-**	**-**	**-**	**-**
2	0	CF -ve	NT	**+**	**+**	**-**	**-**	**-**	**-**	**-**	**-**	**-**	**-**	**-**	**-**
3	0	CF -ve	NT	**-**	**+**	**-**	**-**	**-**	**-**	**-**	**-**	**-**	**-**	**-**	**-**
6	**0**	CFA/I+CS21	O71,NT	**-**	**+**	**-**	**-**	**-**	**-**	**-**	**-**	**-**	**-**	**-**	**-**

*PΦ15 is designated as IMM-002

### CS3 is used as a receptor for the IMM-002 phage

**Fig 2:**
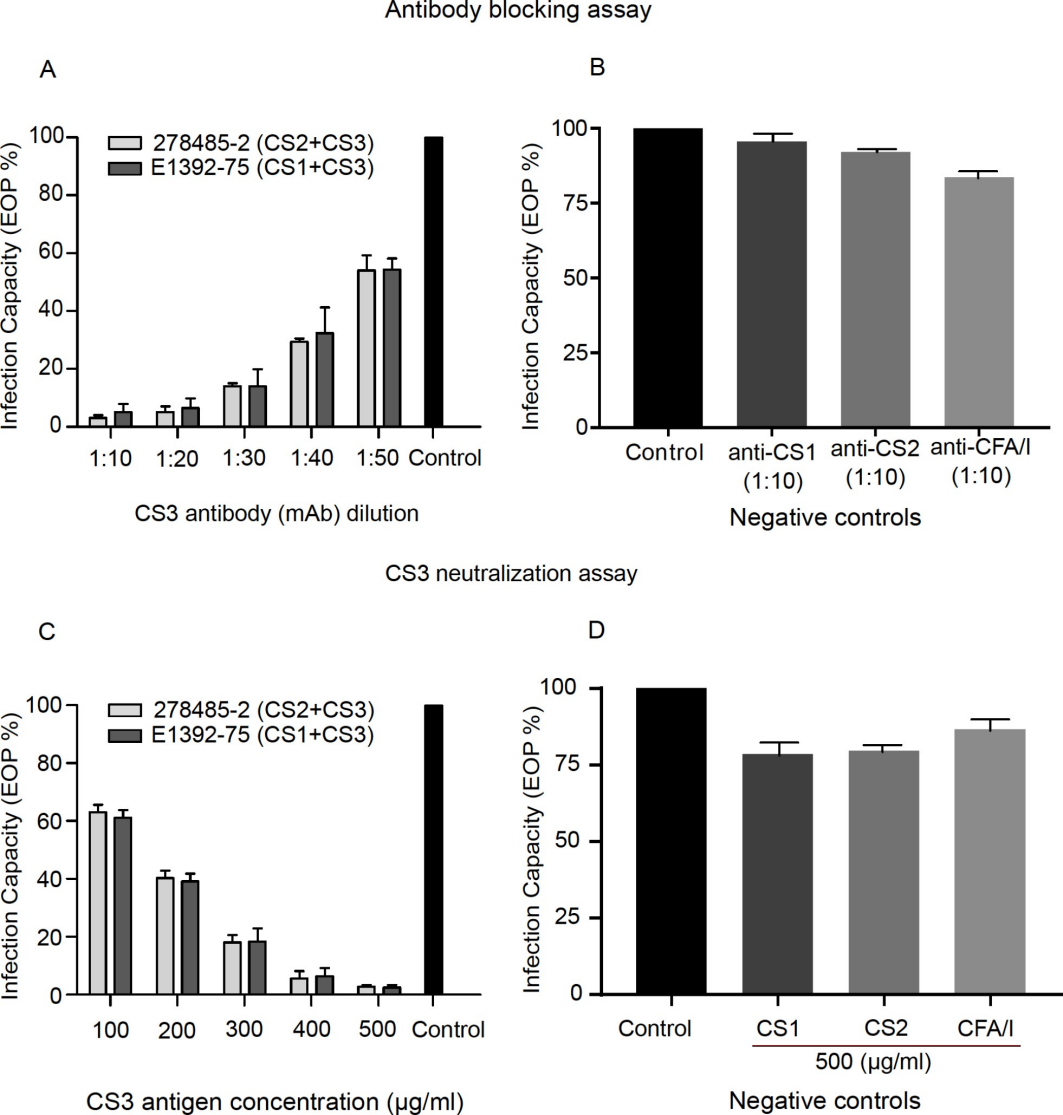
Inhibition of phage IMM-002 infection by antibody blocking using anti-CS3 mAb and CS3 neutralization assay. (A) IMM-002 phage infection capacity against two representative CS3-expressing ETEC strains (278485-2and E1392-75) was tested. After pre-treatment of the ETEC cells with different dilutions of anti-CS3 monoclonal antibody (mAb) ranging from 1:10 to 1:50 the efficiency of plating (EOP %) was determined. Three biological replicate measurements were carried out independently for each of the two different strains. EOP (%) was calculated by taking the average of the biological replicates for the two strains independently. EOP % for 278485–2 (CS2+CS3) and E1392-75 (CS1+CS3) strains are shown by light and dark grey colors respectively. The mean and standard deviation from three biological replicates are shown. The single back bar presents the negative control where the two ETEC strains (278485-2and E1392-75) left without the pre-treatment of anti-CS3 mAb. (B) Negative controls showing the percentage of infection of IMM-002 on CS3-expressing ETEC without mAb pre-treatment (Control) or pre-treated with non-specific mAbs (anti-CS1, CS2 and CFA/I) at 1:10 dilution. (C) Neutralization capacity of purified CS3 fimbrial antigen on IMM-002 phage infection against CS3-expressing ETEC strains (278485-2and E1392-75). The EOP (%) for IMM-002 against two ETEC strains (light and dark grey representing 278485–2 and E1392-75 strains respectively) pre-treated with different concentra0074ions (μg/ml) of purified CS3 antigen is shown. The mean and standard deviation from three biological replicates are shown. (D) Negative controls showing the EOP (%) of IMM-002 phage without purified CS3 pre-treatment (Control) or pre-treated with 500 μg/ml purified CS1, CS2 and CFA/I antigen. The mean and standard deviation from two independent experiments (each with three biological replicates) are shown.

The major subunit (CstA) is the main building block of the CS3 fibrillae and also mediates adherence to the intestinal cells [36].To determine whether the IMM-002 phage specifically targets CS3, an antibody blocking assay was performed using a monoclonal (mAb) anti-CS3. Two representative CS3-expressing ETEC strains were used for the antibody blocking assay: 278485-2(CS2+CS3) and E1392-75 (CS1+CS3). The relative efficiency of plating (EOP) of phage IMM-002 against the representative ETEC strains with or without anti-CS3 antibody was measured. The relative EOP represents the titer of the phage on a given ETEC strain compared to the maximum titer observed for control (without mAb). Blocking the CS3 with the anti-CS3 mAb hampered the infectious capabilities of IMM-002 (Fig 2A). The phage-infection capacity decreased gradually as the concentration of the anti-CS3 antibody increased. Thus, when the anti-CS3 antibody titer was highest (1:10), the lowest EOP (%) was observed on the plates (Fig 2A). The EOP (%) was highest in the control where no anti-CS3 mAb was pre-incubated with the recipient ETEC bacteria (Fig 2A and 2B). To rule out non-specific binding of anti-CS3 mAb, mAbs against unrelated CFs (CS1, CS2, and CFA/I) were tested in antibody blocking assays. None of the mAbs prevented infection by the IMM-002 phage and no significant plaque reduction was observed (Fig 2B). When IMM-002 phages were pre-incubated with a high concentration of CS3 antigen (500 μg/ml), EOP (%) was significantly reduced, whereas phages pre-incubated with a lower concentration of CS3 antigen produced higher EOP (%) (Fig 2C). No significant neutralization activity of CS1, CS2, and CFA/I antigens was seen as the EOP (%) remained unchanged when IMM-002 was pre-incubated with 500 μg/ml of these antigens as in the control (Fig 2D).

Interestingly, IMM-002 did not discriminate between the ETEC strains that express CS3 alone or together with additional CFs (CS1+CS3±CS21 or CS2+CS3±CS21). We show that the IMM-002 phage infection is CS3-depedent by the antibody-blocking assay. CS3 was blocked by an anti-CS3 mAb which binds to the structural subunit CstA of CS3 [37]. This strongly indicates that unoccupied CS3 fibrillar structural proteins (CstA) are necessary for the phage infection and CS3 may act as a receptor or an attachment site for IMM-002. Furthermore, the purified CS3 antigen was shown to neutralize IMM-002 phage infection. This is most likely due to a CS3-specific blockage of the MM-002 tail preventing the phage from binding the CS3 fibrillae. In summary, these experiments provide evidence that unoccupied CS3 is required for the IMM-002 to attach toCS3-expressing ETEC strains.

### Morphology of the IMM-002 phage belongs to *Podoviridae* family

**Fig 3:**
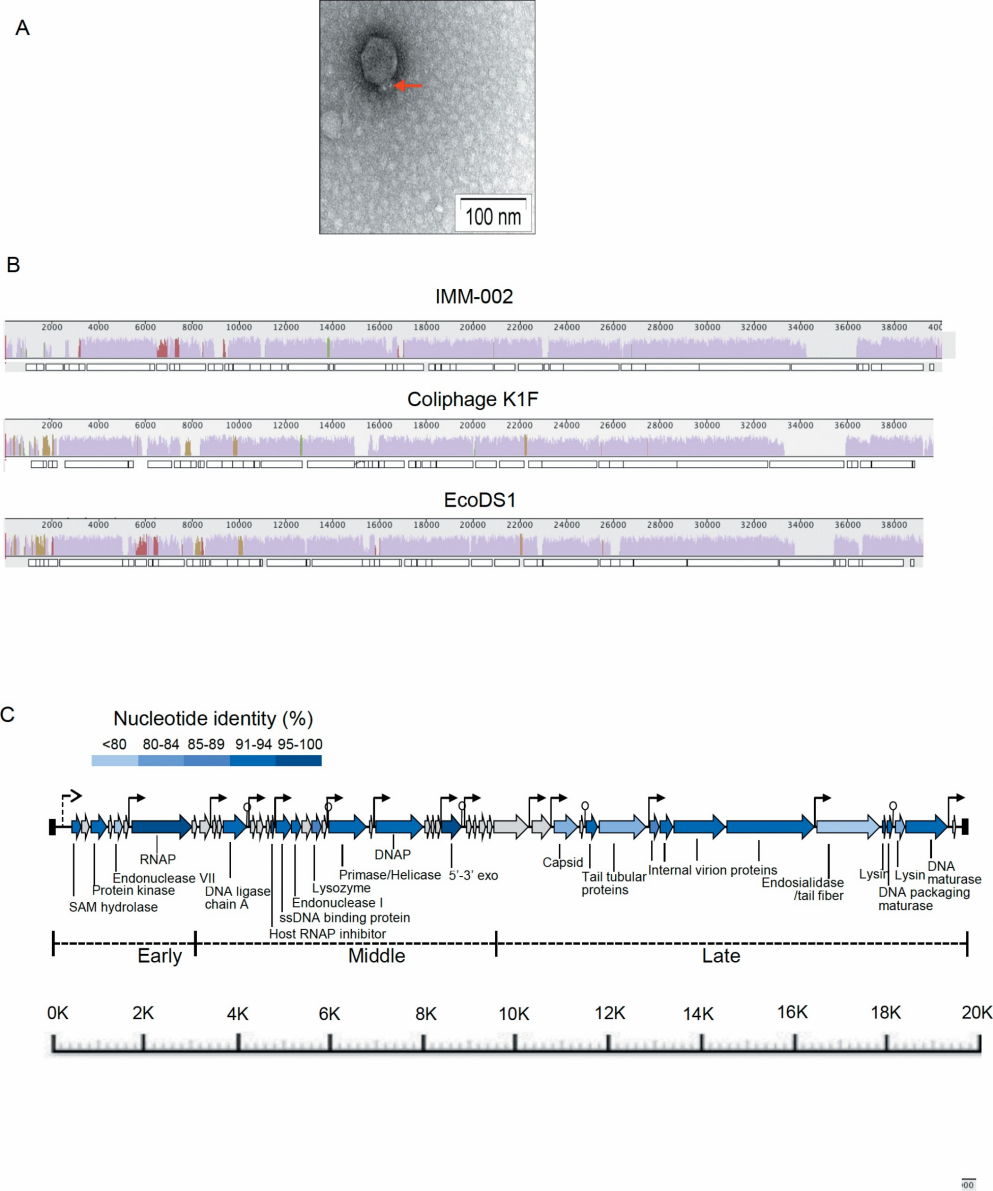
Morphology and Genomic landscape of the IMM-002 phage. (A) Electron micrograph of the CS3-ETEC-specific phage IMM-002. The icosahedral head with a similar diameter as phage T7 and a short tail (indicated by red arrow) of the IMM-002 phage are visible (bar indicates 100 nm). (B) Genome comparison of IMM-002 and the two most closely related *E*. *coli* phages, Coliphage K1F and EcoDS1, using progressiveMauve. The predicted ORFs in each genome are shown below each Mauve graph as rectangles. The degree of nucleotide similarity between aligned regions is indicated by the height of the Mauve-generated similarity profile (colored blocks), where mauve represents the highly conserved core genome, dark-colored segments are conserved in two of the three genomes, and segments without any coloring are unique to that genome. The numbers above each genome are the coordinates for that genome. Nucleotide sequence and gene synteny are for the most part conserved between the three phages. (C) Genomic map of phage IMM-002. Predicted ORFs are indicated with arrows. Predicted annotation is shown below for all homologous genes, with the remainder representing hypothetical genes. The genome is divided up into three gene classes (early, middle and late) by homology to ORFs in phage T7. The dashed line arrow at the far left shows the location of an *E*. *coli* consensus sigma-70 promoter, while the rest of the line arrows indicate the presence of phage-specific promoters. Rho-independent transcriptional terminators are shown by the vertical line and circle symbols. The 189-bp terminal repeats are shown by the solid rectangles. The percent of nucleotide sequence identity between the annotated genes of IMM-002 and reference T7 phage—EcoDS1 (Accession number: NC_011042.1) is shown. The sequence identity (%) is depicted as a gradient color code. The ORFs without any color represents the genes for which no homolog was detected in the EcoDS1 genome.

The electron micrographic analysis showed that the newly isolated phage IMM-002 possess a 7- icosahedral (60 nm) head and a very short tail (Fig 3A). These morphological features of the IMM-002 phage closely resemble a member of *Podoviridae* family [38]. In contrast, the previously isolated IMM-001 phage, specific against CS7-expressing ETEC strains, showed along tail with some curvature [10].

### Sequencing of IMM-002 and IMM-001 phage reveals T7 and *Siphoviridae*-like genomic signatures

In order to investigate the genomic landscape of IMM-002 and the previously identified CS7-specific phage (IMM-001) the phages were whole genome sequenced and analyzed.

Whole-genome BLAST analysis revealed that IMM-002 shared 79% and 80% nucleotide identity with the genomes of two T7 *E*.*coli* phages:ColiphageK1F (Accession number: DQ111067.1) and EcoDS1 (Accession number: NC_011042.1), respectively. Comparative genomic analysis of IMM-002 phage and the two closely related T7 *E*.*coli* phages: Coliphage K1F and EcoDS1 showed that the nucleotide sequence and gene synteny are highly conserved between the three phages (Fig 3B). Furthermore, using the reference phage genomes we identified the known three gene classes (early, middle and late) in the IMM-002 phage: early genes that are primarily involved in phage growth, including RNA polymerase (RNAP); middle genes that drive DNA metabolism; and late genes that are responsible for virion production and host cell lysis. Rho-independent transcriptional terminators were also identified (Fig 3C). The nucleotide sequence comparison between the annotated IMM-002 gene and the reference T7 phage (EcoDS1) is shown as the gradient color (Fig 3C). Among the annotated genes, RNAP (99%), host-RNAP inhibitor (96%) and exonuclease (96%) showed the highest nucleotide identity with EcoDS1. The lowest sequence identity was observed for endonuclease (54%).

An alignment-based sequence logo showed a conserved promoter sequence across the two reference T7 phages and IMM-002 (S1A and S1B Fig). Altogether these results confirmed the identity of IMM-002 phage as T7-phage. Although most T7 phage receptors are known to be lipopolysaccharides [39], there are reports suggesting that T7 may also recognize and utilize other outer membrane proteins present on the host cell [40]. The role of bacterial adhesins as receptors in phage infection is not an unusual phenomenon as it was shown that the *V*. *cholerae* bacteriophage (CTXΦ) utilizes the *V*. *cholera* type IV pilus (known as toxin co-regulated pilus or TCP) as its receptor [41].

In contrast to IMM-002, IMM-001 genome did not exhibit high nucleotide identity with any of the known phages in the NCBI database, whole-genome sequence alignment revealed only 40% and 38% nucleotide identity with two *E*. *coli*-specific *Siphoviridae* phages: Eco ACG-M12 (Accession number: NC_019404) and Eco CEB EC3a (Accession number: KY398841), respectively. These two *E*. *coli*-specific *Siphoviridae* phages (Eco ACG-M12 and Eco CEB EC3a) were used for comparative genomics analysis with respect to IMM-001 (S2A Fig). To further investigate the origin of IMM-001, the amino acid sequence of all annotated proteins were compared to the NCBI database. The protein-based homology analysis revealed that the highest average sequence identity score was obtained for *Siphoviridae*—Eco ACG-M12. S2 Fig depicts the sequence identity of individual proteins between IMM-001 and the closely related Eco ACG-M12. Out of all annotated proteins, the tail fiber protein revealed the highest sequence identity (95%) to the *E*. *coli*-specific phage Eco ACG-M12 that belong to the *Siphoviridae* family.

The genomic analyses of the previously identified phage (IMM-001) targeting CS7-expressing ETEC strains identified 98 ORFs in total, out of which 21 annotated (S2B Fig). Promoter analysis of IMM-001 showed 63% sequence identity when compared to *E*. *coli*-specific phages belonging to the *Siphoviridae*family including (S2C Fig) Eco ACG-M12 and Eco CEB EC3a.

The isolation of two ETEC CF-specific phages belonging to the *podoviral* T7-like genus and *siphoviral* Rtp-like genus suggests that there may be other types of phages targeting additional ETEC CFs and thus may influence the CF profile of circulating ETEC bacteria. The study of phage-host dynamics is necessary for the re-emerging field of phage therapy [42]. Due to the increased prevalence of antibiotic-resistant bacteria, the potential of phage therapy needs to be re-assessed for combating the multi-drug resistance in bacterial pathogens. However, the phage resistance mechanisms in bacteria may weaken the idea of phage-therapy. Therefore, to make phage therapy widely applicable it is necessary to investigate the acquired and built-in phage-resistance mechanisms in the host strains.

### Characterization of CRISPR-Cas systems in CS3 and CS7-expressing ETEC strains

**Fig 4:**
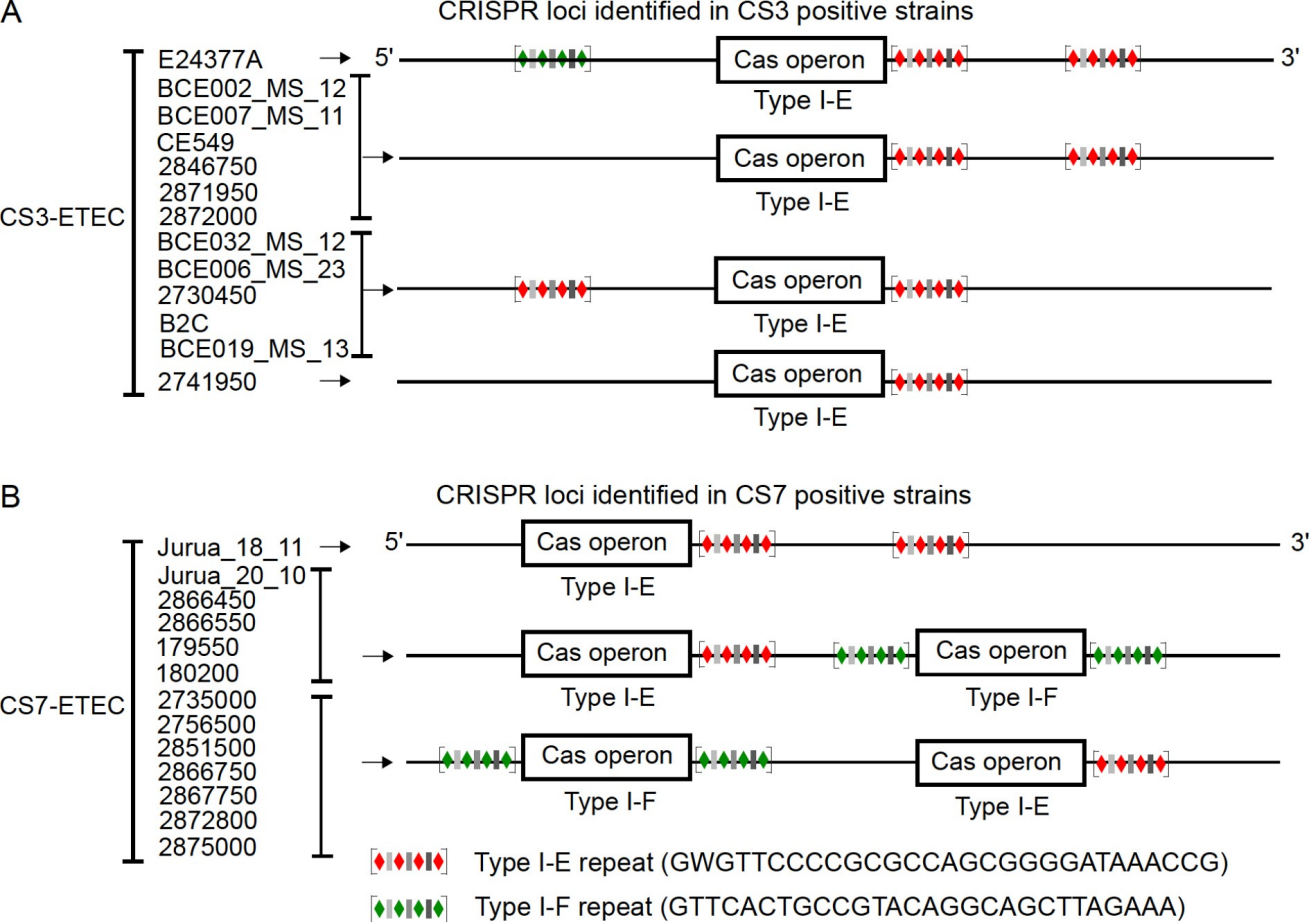
Characterization of direct repeats and *cas* genes within CRISPR arrays of CS3 and CS7-expressing ETEC strains. Schematic diagram showing the organization of *cas* operons and the localization of the CRISPR loci within the genomes of CS3 (A) and CS7 (B)-expressing ETEC strains. The CRISPR loci were identified using CRISPRFinder [44].The diamond and bars represents the direct repeats (DRs) and spacer sequences, respectively. The colors of the CRISPR loci indicate their subtype, green and blue representing Type I-F and Type I-E DRs. The consensus DR sequences for both subtypes are given.

CRISPR-Cas interference mechanism is a defense system which potentially could provide immunity to bacteriophages [43]. Inspecting phage genome sequences alone is not enough to study the CRISPR-mediated phage-host interactions. Hence, it is important to analyze the host genomes of the targeted ETEC strains as well. Therefore, genomes of CS3 (n = 11) and CS7 (n = 13) expressing ETEC strains were analyzed. The CS3-expressing strains also expressed CS1 or CS2 as well as CS21.The selected strains were previously isolated from clinical stool samples and whole genome sequenced (S2 Table). With the aim of uncovering the past history of interactions between ETEC strains and ETEC-specific phages, genomes of CS3 and CS7-expressing ETEC strains were searched for CRISPR loci and *cas* genes. CRISPR finder [44] analysis revealed the presence of multiple confirmed CRISPR loci in the genomes of the ETEC strains (S3 and S4 Tables). Single Type I-E *cas* operon was identified in each of the CS3 expressing ETEC strains with an adjacent CRISPR locus harboring Type I-E repeat sequences (Fig 4A). Additional Type I-E CRISPR loci without associated *cas* operon were identified in all CS3 positive strains except one (2741950). Interestingly, only one CS3 positive ETEC strain (E24377A) harbors a Type I-F orphan CRISPR locus.

In contrast to CS3 expressing ETEC strains, all CS7 expressing ETEC strains, except one (Jurua_18_11) possess two types of *cas* operons, belonging to Type I-E and Type I-F, respectively (Fig 4B). All the Type I-F *cas* operons of CS7 expressing ETEC strains were flanked by two CRISPR loci. Previous characterizations of CRISPR-Cas systems have shown that both commensal and pathogenic *E*. *coli* harbor either the Type I-E or the Type I-F CRISPR-Cas system [21,45]. Additionally, a recent study has shown that unlike Type I-E, Type I-F systems are predominantly found in the *E*.*coli* strains which are susceptible to antibiotics implying a possible negative correlation between the CRISPR I-F system and antimicrobial susceptibility of *E*. *coli* strains [46]. Interestingly, the presence of both Type I-E and Type I-F CRISPR-Cas systems in the analyzed 12 CS7-expressing ETEC strains indicated that pathogenic *E*.*coli* strains are more diverse than previously thought with respect to their CRISPR-Cas content and architecture. Previous studies have proposed a possible target bias, where Type I-E and Type I-F systems preferentially target phages and plasmids, respectively [46]. Corroborating the previous findings, we identified that the ETEC Type I-E CRISPR systems preferentially target invading phage DNA and the ETEC Type I-F CRISPR systems target foreign plasmid DNA by using the CRISPRTarget tool (http://bioanalysis.otago.ac.nz/CRISPRTarget/crispr_analysis.html). The CS7-expressing strains harbored both the Type I-E and I-F CRISPR-Cas systems, indicating that these strains may be equally effective against invading phages and plasmids. Previously a positive correlation has been shown between the presence of CRISPR I-F system and antimicrobial-susceptibility *E*. *coli* [46]. Whether the presence of the Type I-F CRISPR-Cas system makes the CS7 expressing ETEC strains more susceptible to antibiotics remains to be elucidated.

**Fig 5:**
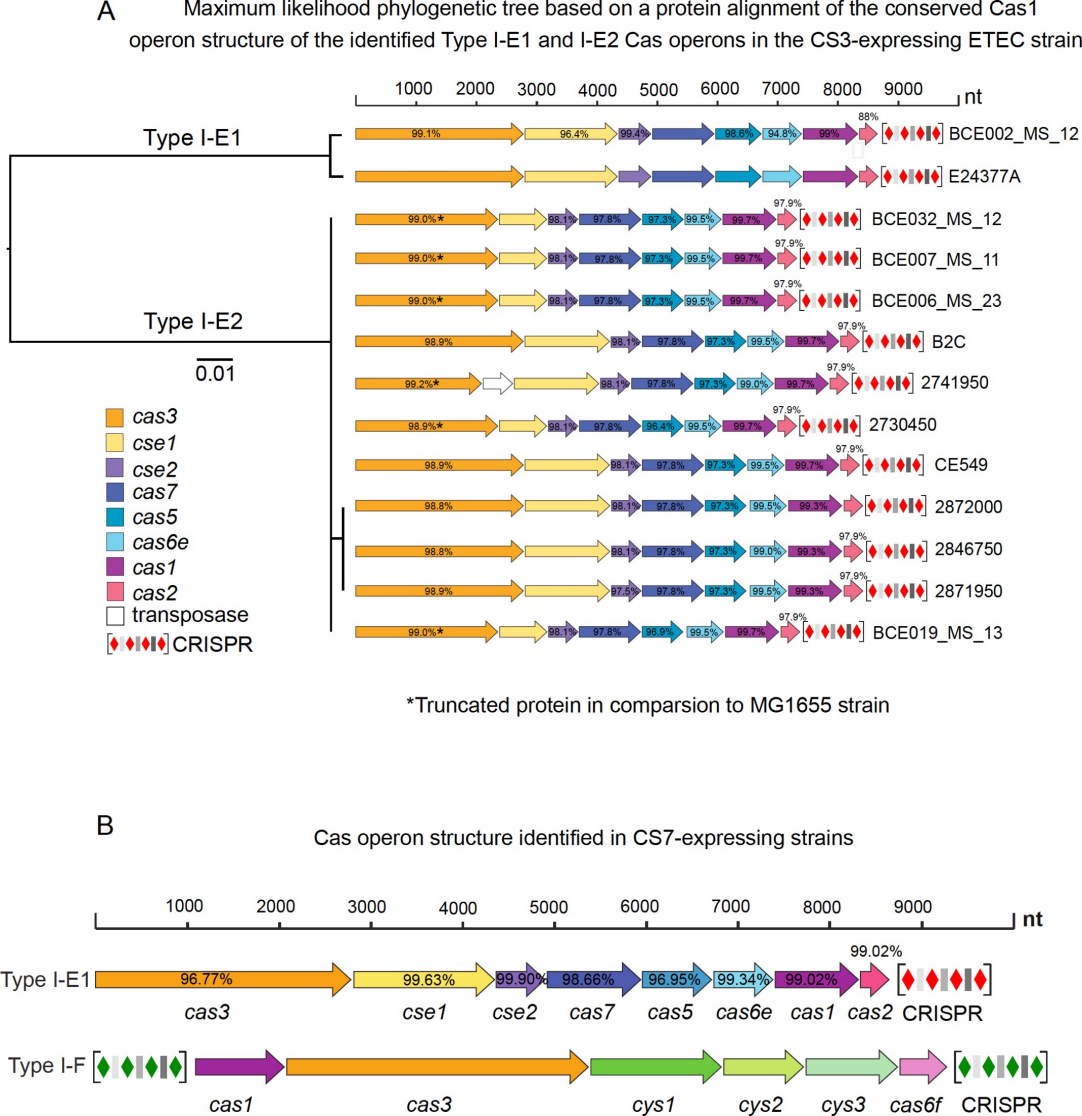
Diversity and genetic organization of *cas* operons in CS3 and CS7-expressing strains. (A) A phylogenetic tree based on the extracted Cas1 amino acid sequences from the CS3-expressing ETEC strains was constructed using the maximum likelihood algorithm. The extracted *cas* operons, belonging to Type I-E, are mapped onto the tree, showing the structure of the operon and the amino acid sequence identity (numbers within the genes) to the reference *E*. *coli* strain MG1655 (See S3A Fig). The two identified variants (E1 and E2) of the Type I-E *cas* operon are indicated in the tree. The truncated Cas3 protein is shorter than the Cas3 of the reference (MG1655), missing the end of the sequence (*) (A). The two types of *cas* operons identified in the CS7-expressing ETEC strains, Type I-E and Type I-F are shown. The number within in the genes indicate the amino acid sequence identity (%) between the encoded genes of the Type I-E *cas* operon and that of the reference *E*. *coli* strain MG1655 (Type I-E2) and E24377A (Type I-E1). The tree scale representing an evolutionary distance of 0.01 is given. A ruler for nucleotide length starting from 0 to 10,000 nucleotide is given as a measure of the length of the *cas* operons in each strain.(B) Type I-E and Type I-F *cas* operons of CS7 expressing ETEC strains, composed of a distinct set of *cas* genes are shown. The amino acid sequence identity (numbers within the genes) with respect to the reference strain E24377A (Type I-E1) is indicated (See S3A Fig).

Previous phylogenetic analysis of the Cas1 and Cas2 proteins present in *E*. *coli* have shown that the Type I-E can be grouped into two subtypes, Type I-E1 and Type I-E2 [45]. The structure of the *cas* operons and the location of the associated CRISPR repeats in the CS3 and CS7-expressing strains were compared with *cas* operons of two reference strains, the commensal *E*. *coli* K-12 MG1655 (Accession number: U00096.1)and the ETEC reference strain E24377A (Accession number: CP000800.1). MG1655 and E24377A harbor Type I-E2 and Type I-E1 CRISPR-Cas systems, respectively [45] and they both lack Type I-F repeat sequences and associated I-F *cas* operons (Fig 4 and S3 Fig). Similar to the MG1655 strain (I-E2), the CS3 and CS7-expressing ETEC strains harbor two CRISPR loci. The Cas proteins of CS3-expressing ETEC strains are most similar to the Type I-E2, except one BCE002-MS12 which is highly similar to the Type I-E1 *cas* operon of E24377A (Fig 5 and S3 Fig). A phylogenetic tree based on the amino acid sequence alignment of the conserved gene encoding Cas1 of each of the *cas* operons identified in the CS3-expressing strains showed that two strains, BCE002_MS_12 and E24377A belonging to the subtype I-E1, were evolutionary distant to the rest of the strains that exhibited high sequence identity to Cas1 of the reference strain MG1655 (I-E2) (Fig 5A).This result corroborates the occurrence of two variants (E1 and E2) of Type I-E CRISPR-Cas system in the CS3 expressing ETEC strains.

Furthermore, a comparison of the *cas* operon structure of the CS3-expressing strains revealed that *cas* operons in several strains (BCE032_MS_12, BCE007_MS_11, BCE006_MS_23, 2730450, and BCE019_MS_13) encompass a truncated *cas3* gene. The strain 2741950 also has a truncated *cas3* gene, however, this is most likely due to the insertion of an insertion sequence between *cas3* and *cse1*. Interestingly, it has been previously shown that insertion sequences may contribute to the variability and movement of the Type I-E *cas* genes in *E*. *coli* [45,47]. Thus, the presence of insertion sequences may influence the evolution of CRISPR-Cas systems as well as serve as a vehicle of horizontal transfer of CRISPR-Cas loci between ETEC strains.

Protein analysis of the truncated version of Cas3 (785 amino acids) compared to Cas3 in the reference strain MG1655 (888 amino acids) showed that they both have the conserved DEAD/DEAH box Helicase domain indicating that the Cas3 protein may still be functional. However, further studies are required to confirm the functional capacity of the truncated version of Cas3. Furthermore, the strains with a truncated *cas3* gene also have a truncated *cse1* gene. The *cse1* gene product containing a zinc-binding motif coordinated by four cysteine residues has been shown to interact with Cse2B and Cas5proteins within the Cascade complex [48]. Cse1 also facilitates the double-stranded target DNA binding activity of Cascade complex [49]. Interestingly, the zinc and DNA binding domains are intact in the truncated *cse1* gene, indicating that this protein is also most likely fully functional. Sequence comparison of Cse2, Cas7, Cas5, Cas6e-E, Cas1 and Cas2 proteins belonging to the Type I-E2 *cas* operons of CS3-expressing ETEC strains to the corresponding proteins in the reference strain MG1655 (Type I-E2) revealed that the Type I-E2 *cas* operon were highly conserved, sharing ≥96.4% of their amino acid sequence with MG1655 strain (Fig 5A). Moreover the structure of the Type I-E operons of the CS3-expressing ETEC strains (I-E1 and I-E2), CS7-expressing ETEC strains (Type I-E1) and the reference strains—MG1655 (Type I-E2) and E24377A (Type I-E1) were identical to each other (Fig 5, S3A Fig).

The I-E *cas* genes (Cas3, Cse1, Cse2, Cas7, Cas5, Cas6e, Cas1 and Cas2) of the CS7 expressing ETEC strains shared more than 96.7% of their protein sequence with the corresponding proteins of the E24377A (I-E1) reference strain (Fig 5B). Unlike the CS3 expressing ETEC strains, the Type I-E *cas* operons of CS7-expressing ETEC strains belonging to Type I-E1 variant were highly conserved and devoid of any Type I-E2 *cas* operon. Akin to the Type I-E1 *cas* operon, I-F *cas* genes of CS7-expressing ETEC strains exhibited highly conserved sequences. The Type I-F *cas* operon was composed of six genes including two core genes (cas2 and cas3) and four subtype genes (*cys1*, *cys2*, *cys3 and*,*cas6f*) (Fig 5B).

In summary, we report the presence of multiple Type I-E CRISPR-Cas loci in CS3 (in combination with CS1, CS2, and CS21) and both Type I-E and Type I-F CRISPR-Cas loci in CS7-expressing ETEC strains. In-depth analysis revealed that the Type I-E operons of the analyzed CS3-expressing strains encompass two variants—I-E1 and I-E2. Unlike the CS3 expressing ETEC strains, highly conserved Type I-E (I-E1) and Type I-F cas-operons were identified in CS7-positive ETEC strains. Previously it was proposed that *E*. *coli* strains may carry CRISPR-Cas systems that belong to either type I-E or type I-F [50]. The co-occurrence of type I-E or type I-F were uncommon since only a single *E*.*coli* strain has been identified to harbor both I-E and I-F *cas* genes [51]. The co-occurrence of I-E and I-F *cas* operons in the CS7-expressing ETEC strains uncovers the diverse nature of CRISPR-Cas content of pathogenic *E*.*coli* strains.

It has been shown that CRISPR locus can be transferred horizontally as a complete package between bacterial strains [13] and the presence of insertion sequences within the CRISPR locus may be involved in the horizontal transfer of CRISPR locus between bacteria their natural habitats. In light of this augment, the presence of insertion sequences within the CRISPR locus of the CS3-expressing ETEC strain—2741950 signifies the possibility of horizontal transfer of CRISPR locus among pathogenic *E*.*coli* strains. The genomic analysis of CRISPR-Cas systems shows that both CS3- and CS7-expressing ETEC strains harbor the necessary CRISPR locus, phage-specific spacers, and *cas* operons to efficiently execute the CRISPR mediated interference to combat CF-specific phage infections.

### Identification of IMM-002 and IMM-001 specific spacer sequences within CS3 and CS7 expressing ETEC strains

To identify the putative spacer sequences that are specifically targeting the IMM-001 and IMM-002 phages, the spacer sequences within the CRISPR arrays residing in the genomes of the CS3- and CS7-expressing ETEC strains were aligned with the IMM-002 and IMM-001 phage genomes, respectively. All the candidate spacer sequences were further filtered based on the PAM sequence and seed-complementarity. Spacer sequences were considered phage specific if they hybridized with protospacers that have intact canonical Type I-E PAM sequences (AAG, ATG, AGG, and GAG) with no mismatches. Additionally, identified spacers should be 100% complementary to protospacer sequences in the seed region.

In total, two putative spacers targeting protospacer sequences in the IMM-002 genome were identified in the CS3-expressing ETEC strains. The IMM-002 specific spacers were present in six of the thirteen screened CS3-ETEC strains (Table 3). Only one of the CS7-expressing ETEC strains lacked IMM-001-specific spacers and the other twelve harbored two putative spacers (Table 4).

**Table 3:** Identification of spacer sequences, PAM and phage-resistance phenotype in CS3-expressing ETEC strains.

Isolate ID	Spacer sequence	Identity (%)^1^	Seed (%)^2^	PAM^3^	PAM type	Phage infection^4^
BCE007_MS_11	AACGTCAGGTTGTCGCCGCTCTGCGTGGTCGC	81 (26/32)	100 (7/7)	GAG	I-E	Resistant
BCE019_MS_13						
BCE032_MS_12						
2730450						
2871950						
E24377A						
BCE007_MS_11	CTGCTGCTCGAGCTGGTGGAGTGCTGCTATAG	72 (23/32)	100 (7/7)	AGG	I-E	Resistant
BCE019_MS_13						
BCE032_MS_12						
2730450						
2871950						
E24377A						
BCE002_MS12	No spacer	N/A	N/A	N/A	N/A	Susceptible
BCE006_MS_23						
2741950						
2846750						
2872000						
B2C	No spacer	N/A	N/A	N/A	N/A	Not tested
CE549						

^1^ Identity (%) represents the % of nucleotide matching between ETEC spacer and IMM-002 protospacer sequences

^2^Seed (%) represents the % of nucleotide matching in the seed region between spacer and protospacer sequences

^3^ PAM indicates "Protospacer Adjacent Motif" that is located in the genome of IMM-002 phage

4 Phage infection indicates the plaque-assay which determined the susceptible or resistance phenotype of ETEC strains

**Table 4:** Identification of spacer sequences, PAM and phage-resistance phenotype in CS7 expressing ETEC strains.

Isolate ID	spacer sequence	Identity(%)^1^	Seed (%)^2^	PAM^3^	PAM type	Phage infection^4^
179550	TGCAAAACAAAACTGTATTGATCGCGTTTTGT	88 (28/32)	100 (7/7)	AGG	I-E	Resistant
180200						
2735000						
2756500						
2851500						
2866450						
2866550						
2866750						
2867750						
2872800						
2875000						
Jurua_20_10						
179550	TGGCATGCAATCACTACAGCTATTAATTTCTA	60 (19/32)	100 (7/7)	AAG	I-E	Resistant
180200						
2735000						
2756500						
2851500						
2866450						
2866550						
2866750						
2867750						
2872800						
2875000						
Jurua_20_10						
Jurua_18_11	No spacer	N/A	N/A	N/A	N/A	Susceptible

^1^ Identity (%) represents the % of nucleotide matching between ETEC spacer and IMM-001 protospacer sequences

^2^Seed (%) represents the % of nucleotide matching in the seed region between spacer and protospacer sequences

^3^ PAM indicates "Protospacer Adjacent Motif" that is located in the genome of IMM-001 phage

^4^ Phage infection indicates the plaque-assay which determined the susceptible or resistance phenotype of ETEC strains.

The identification of bacterial spacer sequences that were complementary to phage protospacers may indicate that the CS3 and CS7-expressing ETEC strains may have equipped with CRISPR-Cas resistance mechanism against the targeted phage genomes. To uncover whether the CRISPR-Cas systems of ETEC strains target IMM-001 and IMM-002 phages, the genome sequences of IMM-001 and IMM-002 were analyzed. The presence of IMM-002 and IMM-001-specific spacer sequences within the CRISPR locus of six CS3 and twelve CS7-expressing ETEC strains, respectively, hints towards possible CRISPR-Cas mediated resistance against these phages. Interestingly, the CRISPR-spacer sequences targeting the phages were not identified in all the analyzed thirteen CS3 and CS7 positive ETEC strains (Table 3 and Table 4). The strain-specific CRISPR spacers targeting the phage genomes may reflect the history of past encounters of phage invasion in these ETEC strains. The presence of spacers targeting specific IMM-002 and IMM-001 protospacers could also be due to the horizontal transfer of CRISPR-loci between ETEC strains.

### Phage-specific spacers in CRISPR-Cas systems determines the susceptibility to phage infection of ETEC strains

**Fig 6:**
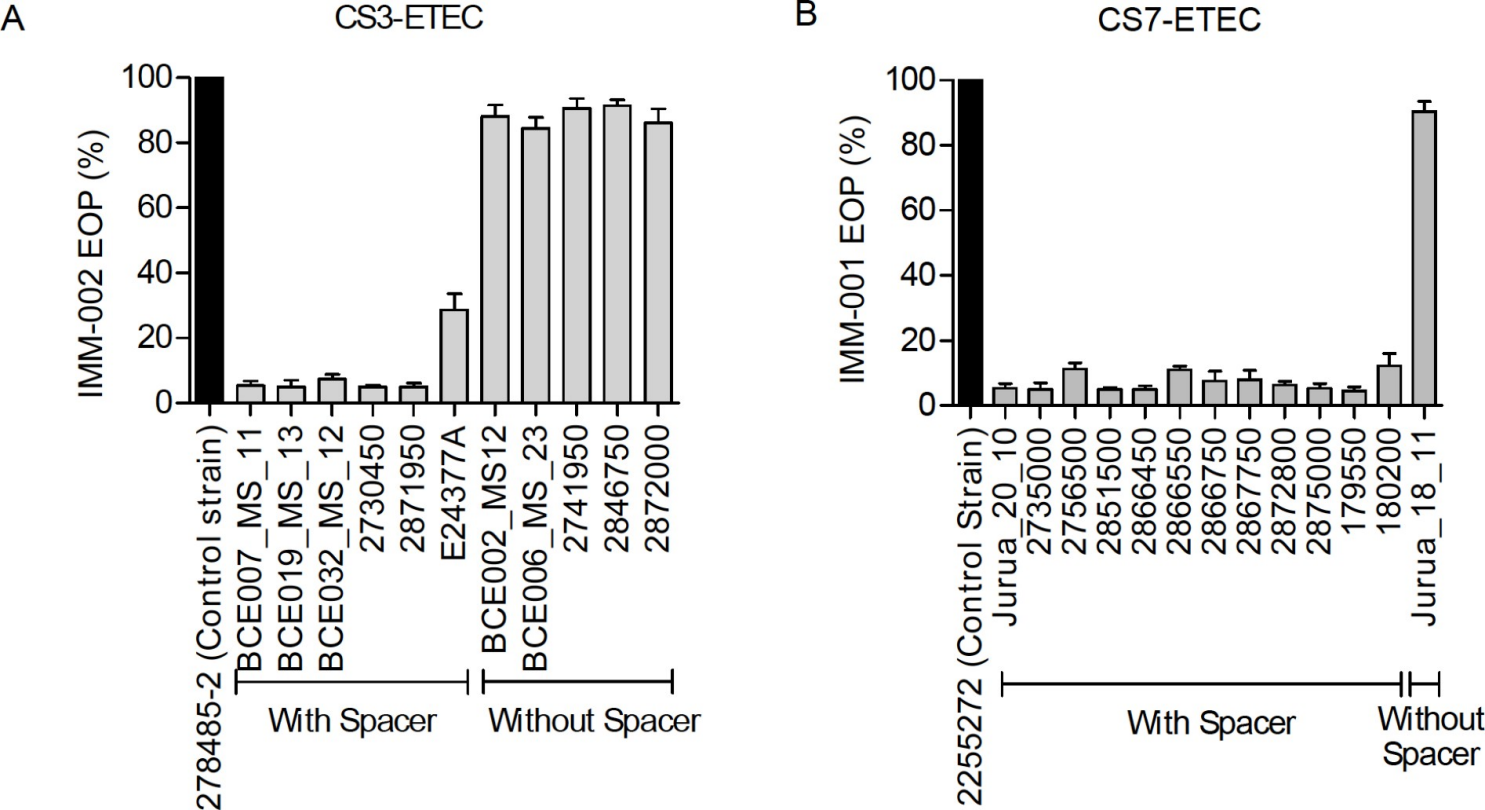
Validation of the efficiency of phage specific CRISPR-Cas systems in CS3 and CS7 expressing ETEC strains. The plaque assay results as calculated by EOP (%) representing the susceptibility of CS3 (A) and CS7 (B) expressing ETEC strains against IMM-002 and IMM-001 phages are shown. Out of the eleven CS3-ETEC strains, six harbored IMM-002 specific spacer sequences while the rest were devoid of IMM-002 specific spacers. Among the thirteen CS7-ETEC strains; twelve harbored IMM-001 specific spacers and were resistant to IMM-001 phage infection. One strain (Jurua_18_11) did not contain the phage-specific spacers and was thus, susceptible to IMM-001 phage infection. The percentage of infection (EOP) of IMM-002 and IMM-002 phages were calculated from the plaque assay. Two strains (CS3: 278485-2and CS7: 225572) were used as negative controls for IMM-002 (CS3-specific) and IMM-001 (CS7-specific) plaque assays.

To investigate whether the presence of phage-specific spacers conferred protection against infection of the specific phage, eleven CS3-expressing and thirteen CS7-expressing ETEC strains were tested for phage infection by plaque assay. The results showed the six CS3- expressing ETEC strains (BCE032_MS_12, BCE019_MS_13, BCE007_MS_11, BCE006_MS_23, 2741950 and 2730450) harboring the two identified phage specific spacers targeting protospacers in IMM-002 were resistant to IMM-002 phage infection (Fig 6A). The five CS3-ETEC strains lacking IMM-002 specific spacers (BCE002_MS12, BCE006_MS_23, 2741950, 2846750 and 2872000) were susceptible to IMM-002 phage infection (Fig 6A). Twelve CS7 expressing ETEC strains (isolate IDs: Jurua_20_10, 2875000, 2872800, 2867750, 2866750, 2866550, 2866450, 2756500, 2735000, 180200, 179550, and 2851500) harboring IMM-001 phage-specific spacers showed resistance to the phage infection whereas one CS7- expressing ETEC strain (Jurua_18_11) lacking the IMM-001 specific spacer sequences was susceptible to phage infection (Fig 6B). The strains lacking the phage targeting spacers may have lost these sequences during genome replication or may not have been invaded by IMM-001 and IMM-002 previously. This was clear, as CS3- and CS7-expressing ETEC strains without the phage-specific spacer sequences were not resistant to phage infection.

**Fig 7:**
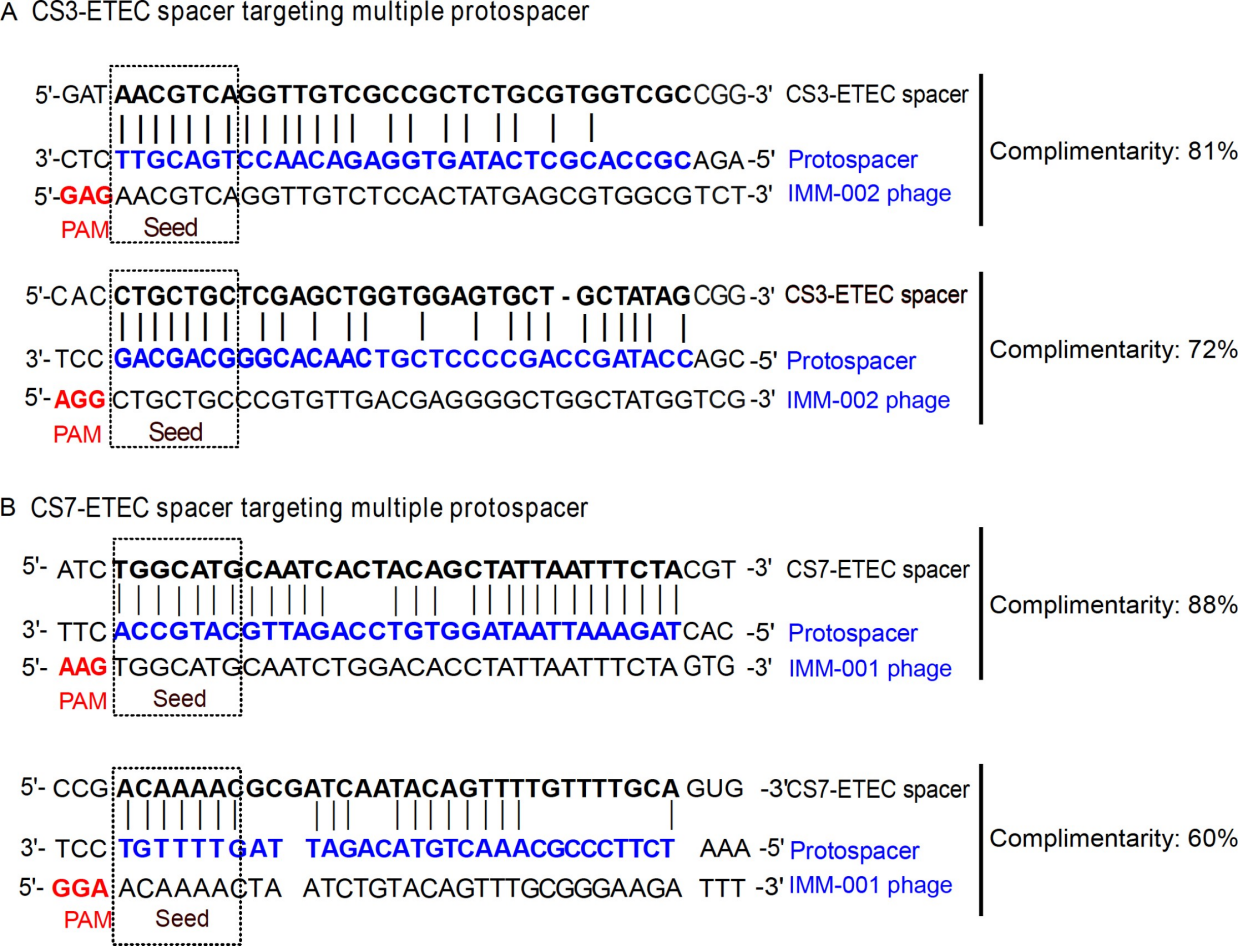
CRISPR spacers of CS3 and CS7-expressing ETEC targeting specific protospacers within IMM-002 and IMM-001 phage genomes. The complementarity between the identified spacers and protospacers in the CS3-expressing ETEC strains (A) and the CS7-expressing ETEC strains (B) are shown by sequence alignment and as the percentage of nucleotide identity. The PAM (red) and seed sequences (dashed line box) are indicated. The targeted protospacer is shown as double-stranded DNA (blue).

The alignment of putative spacers from CS3-positive strains showed 81% and 72% sequence identity to the IMM-002 protospacer sequences with GAG and AGG PAM respectively (Fig 7A and Table 3). The same pattern of spacer-protospacer matching was seen for the CS7-positive strains and IMM-001 phage. Among two identified spacers of CS7-ETEC strains one spacer showed 88% with the IMM-001 protospacer with AGG PAM while the other spacer showed 60% with AAG PAM associated protospacer from CS7-specific phage IMM-001 (Fig 7B, and Table 4). The seed sequences of both IMM-001 and IMM-002 protospacers were 100% complementary to each of the corresponding spacers, indicating that these phages are most likely still able, to be targeted by the CRISPR-Cas systems.

Additionally we performed the screening of potential spacers with less stringent criteria by allowing more mismatch between spacer-protospacer pair. Indeed, we identified one potential protospacers for IMM-002 with these less stringent criteria. Interestingly, the newly identified protospacer did not exhibited any miss-match in the seed region, rather sowed a nucleotide mismatch in PAM sequence (S4 Fig). The mutation in the PAM of this protospacer may not generate effective CRISPR-Cas resistance against the phage IMM-002 [18] and thus may not be utilized as a target protospacer by CS3-expressing ETEC strains. This IMM-002 protospacer with mutated PAM sequence may point towards selective evolutionary pressures excreted by the ETEC CRISPR-Cas system on the phage genome.

One can argue that there could be other mechanisms responsible for this apparent resistance phenotype of certain ETEC strains. One of the most predominant mechanisms by which host bacteria becomes resistant to phage infection includes the receptor modification. A study conducted by Qimron *et al*. showed that mutations in the genes that encode the lipopolysaccharide receptor for T7-phage adsorption lead to the phage-resistance phenotype in *E*.*coli* strains[52]. The phenomenon of receptor mutation-driven phage resistance is also shown for other bacterial strains such as *Yersinia pestis* [53] and *Listeria monocytogenes* [54]. Therefore, it is natural to assume that the “phage-resistance” pattern in the analyzed CS3 and CS7-expressing ETEC strains in this study may occur due to modification of the receptor. The translated amino acid sequences of the major subunits of the colonization factors CS3 and CS7 from the selected ETEC strains were compared to the reference sequences of CS3 (Accession: FN822745.1, p1018) and CS7 (Accession: AY009095) major subunits. The amino acid sequence of the CS3 and CS7 major subunits were 100% identical to the reference sequences, except in two CS7-expressing strains (Jurua_20_10 and 2866450), where the major subunit had one amino acid replacement, isoleucine (ile) to leucine (leu). The substitution of Ile to leu is less likely to change the confirmation or the functional capacity of the CS7 CF since these are both hydrophobic amino acids. The results from the amino acid sequence comparison argue against the hypothesis that receptor modification may be responsible for the resistance showed by certain CS3- and most of the CS7-expressing ETEC strains.

The dynamics of the prevalence of ETEC strains with different CFs may be correlated with the presence of CF-specific phages in the environment at a particular time period. A previous study, Begum *et al*. showed that during the period of 1996 to 1998 CS5+CS6 expressing ETEC strains were predominant [55] in Bangladesh, but in 2008 CS7-ETEC strains emerged and became more prevalent during an epidemic caused by the flood in 2008 at Dhaka, Bangladesh [55].

These findings indicate how phages may affect the population dynamics of ETEC strains with specific CF profiles in both environmental sources and within the human host. However, the underlying reason for the observed shifts in CF prevalence among ETEC strains still remains to be explored.

## Conclusions

The promise of phage therapy in combating the antibiotic-resistance bacteria in their natural habitats and in human gut has become more interesting due to the emergence of multi-drug resistant bacteria. One of the advantages of phage therapy is the specificity towards pathogenic strains, leaving the normal microflora largely unaltered. Phage therapy should include a cocktail of phages which is capable of lysing all pathogenic strains of a certain bacterial species. The existence of ETEC-CF-specific phages in the environment as reported by this and a previous study may encourage large-scale studies to identify other lytic phages specific to ETEC virulence factors and can serve as promising candidates in treating ETEC mediated diarrhea. One of the important features of the two characterized ETEC specific phages—IMM-001 and IMM-002—is that these are not temperate in nature but virulent phages. This virulent nature of these phages makes them suitable candidates for phage therapy and prophylaxis. However, the current study points towards the host-phage arms race and possible phage resistance mechanism that may pose a hindrance to the potential of phage-therapy. It is possible that CRISPR mediated phage-resistance phenotypes are transferred through horizontal transfer between bacterial strains, in addition to incorporating new spacers into a vertically descending CRISPR locus. Overall, the study highlighted the complex interaction between phages and their host strains in their natural environments.

## Supporting information

S1 Fig Analysis of the conserved T7 promoter sequence. (A) Sequence logo for the IMM-002 conserved promoter sequence. Logo was generated using WebLogo (http://weblogo.berkeley.edu/). (B) Comparison of IMM-002 phage promoter consensus sequences with closely related phages (IUPAC single letter DNA notation).
(PDF)

S2 Fig Genomic landscape of IMM-001 phage. (A) Genome comparison of the annotated IMM-001 and the two most closely related Enterobacteria phages, vBEcoS ACG-M12 and vBEcoS CEB EC3a, using progressiveMauve. The degree of nucleotide similarity between aligned regions is indicated by the height of the Mauve-generated similarity profile (colored blocks), where mauve represents the highly conserved segments across all three genomes, dark red represents conserved segments between IMM-001 and vbEcoS ACG-M12, green represents conserved segments in the vB ECOS ACG-M12 and vBEcoS CEB EC3a and yellow indicates the conserved regions in IMM-001 and vBEcoS CEB EC3a. The numbers above each genome are the coordinates for that genome. (B) Genomic map of phage IMM-002. Predicted ORFs are shown by arrows. Numbering above the ORFs (not all ORFs are numbered–it seems as they are all numbered!) is according to phage T1 nomenclature. Predicted annotation is shown below for all homologous genes. The line arrow at the far left shows the location of an *E*. *coli* consensus sigma-70 promoter, while the rest of the line arrows indicate the presence of phage-specific promoters. Percent of nucleotide sequence identify between the annotated genes of IMM-001 and reference *Siphoviridae* phage—Eco ACG-M12 (Accession: NC_019404) has been shown as color code. (C) Sequence comparison of IMM-001 phage promoter consensus sequences with the closely related reference phages vBEcoS ACG-M12 and vB CEB EC3a (IUPAC single letter DNA notation).
(PDF)

S3 Fig Structure of the *cas* operon and associated CRISPR repeat loci of the reference *E*. *coli* strain MG1655. The location of CRISPR loci is indicated by “[]” where the diamond and bars represents the DR and spacer sequences, respectively. The genetic organization of the *cas* operon of MG1655 is depicted and color-coded according to the *cas* genes.
(PDF)

S4 Fig Alignment of ETEC spacer and IMM-002 protospacer with mutated PAM. The complementarity between the putative spacers and protospacers in the CS3-expressing ETEC strains are shown by sequence alignment. The PAM (red) sequence is indicated. The potential single nucleotide mutation is indicated by star symbol. The protospacer is shown as double-stranded DNA (blue).
(PDF)

S1 Table Susceptibility of IMM-002 against ETEC and common *Enterobacteriaceae*. (PDF)

S2 Table List of whole-genome sequenced CS3 and CS7 expressing ETEC strains. (PDF)

S3 Table CRISPR loci of CS3-ETEC. (PDF)

S4 Table CRISPR loci of CS7-ETEC. (PDF)

S5 Table Cas proteins comparison of ETEC strains with that of the reference strains. (PDF)

S1 File Genbank file for IMM-002_T7_phage and Genbank file for IMM-001_Rtp_phage. (PDF)
