# Correction: Phenotypic and genomic analyses of bacteriophages targeting environmental and clinical CS3-expressing enterotoxigenic *Escherichia coli* (ETEC) strains

**DOI:** 10.1371/journal.pone.0213612

**Published:** 2019-03-05

**Authors:** Sajib Chakraborty, Astrid von Mentzer, Yasmin Ara Begum, Mehnaz Manzur, Mahmudul Hasan, Amar N. Ghosh, M. Anwar Hossain, Andrew Camilli, Firdausi Qadri

The second author's initials appear incorrectly in the citation. The correct citation is: Chakraborty S, von Mentzer A, Begum YA, Manzur M, Hasan M, Ghosh AN, et al. (2018) Phenotypic and genomic analyses of bacteriophages targeting environmental and clinical CS3-expressing enterotoxigenic *Escherichia coli* (ETEC) strains. PLoS ONE 13(12): e0209357. https://doi.org/10.1371/journal.pone.0209357
