# Supplementary figures and images for: Phenotypic and genomic analyses of bacteriophages targeting environmental and clinical CS3-expressing enterotoxigenic Escherichia coli (ETEC) strains
Source: PLoS One. 2018 Dec 20;13(12):e0209357. doi: 10.1371/journal.pone.0209357 (PMC6301781; doi:10.1371/journal.pone.0209357)

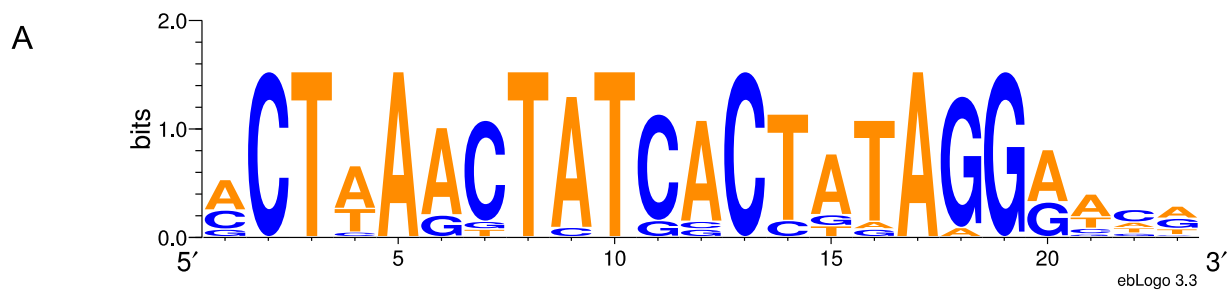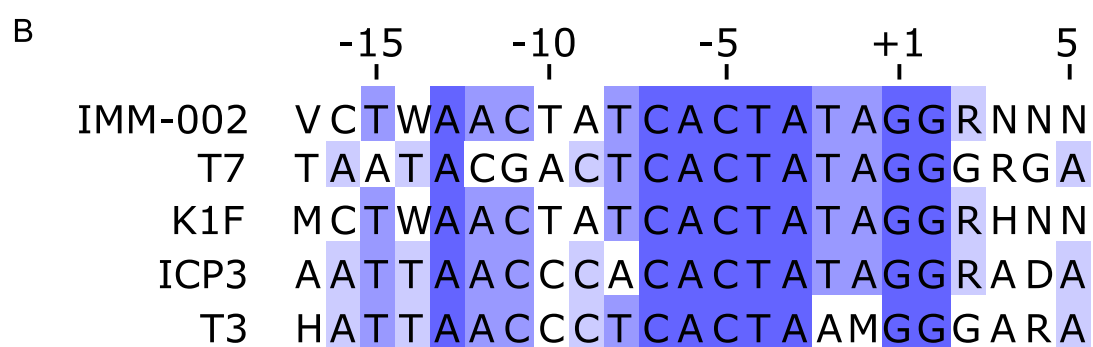

Supplement: S1 Fig — (A) Sequence logo for the IMM-002 conserved promoter sequence. Logo was generated using WebLogo (http://weblogo.berkeley.edu/). (B) Comparison of IMM-002 phage promoter consensus sequences with closely related phages (IUPAC single letter DNA notation). (PDF) [file pone.0209357.s001.pdf]

# IMM-002 protospacer with mutated PAM

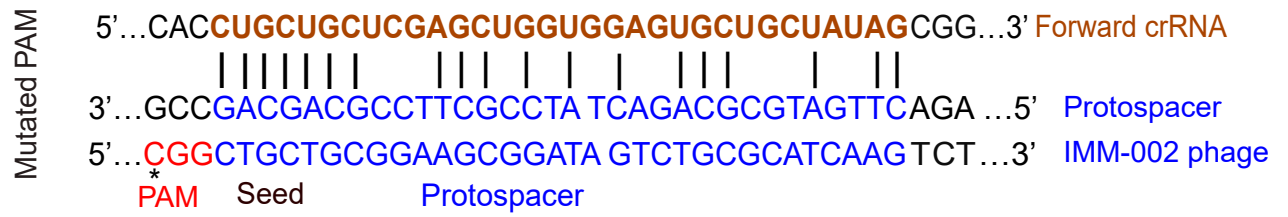

Supplement: S4 Fig — The complementarity between the putative spacers and protospacers in the CS3-expressing ETEC strains are shown by sequence alignment. The PAM (red) sequence is indicated. The potential single nucleotide mutation is indicated by star symbol. The protospacer is shown as double-stranded DNA (blue). (PDF) [file pone.0209357.s004.pdf]
