# Supplementary material for: Phenotypic and genomic analyses of bacteriophages targeting environmental and clinical CS3-expressing enterotoxigenic Escherichia coli (ETEC) strains
Source: PLoS One. 2018 Dec 20;13(12):e0209357. doi: 10.1371/journal.pone.0209357 (PMC6301781; doi:10.1371/journal.pone.0209357)
Supplement: S2 Fig — (A) Genome comparison of the annotated IMM-001 and the two most closely related Enterobacteria phages, vBEcoS ACG-M12 and vBEcoS CEB EC3a, using progressiveMauve. The degree of nucleotide similarity between aligned regions is indicated by the height of the Mauve-generated similarity profile (colored blocks), where mauve represents the highly conserved segments across all three genomes, dark red represents conserved segments between IMM-001 and vbEcoS ACG-M12, green represents conserved segments in the vB ECOS ACG-M12 and vBEcoS CEB EC3a and yellow indicates the conserved regions in IMM-001 and vBEcoS CEB EC3a. The numbers above each genome are the coordinates for that genome. (B) Genomic map of phage IMM-002. Predicted ORFs are shown by arrows. Numbering above the ORFs (not all ORFs are numbered–it seems as they are all numbered!) is according to phage T1 nomenclature. Predicted annotation is shown below for all homologous genes. The line arrow at the far left shows the location of an E. coli consensus sigma-70 promoter, while the rest of the line arrows indicate the presence of phage-specific promoters. Percent of nucleotide sequence identify between the annotated genes of IMM-001 and reference Siphoviridae phage—Eco ACG-M12 (Accession: NC_019404) has been shown as color code. (C) Sequence comparison of IMM-001 phage promoter consensus sequences with the closely related reference phages vBEcoS ACG-M12 and vB CEB EC3a (IUPAC single letter DNA notation). (PDF) [file pone.0209357.s002.pdf]

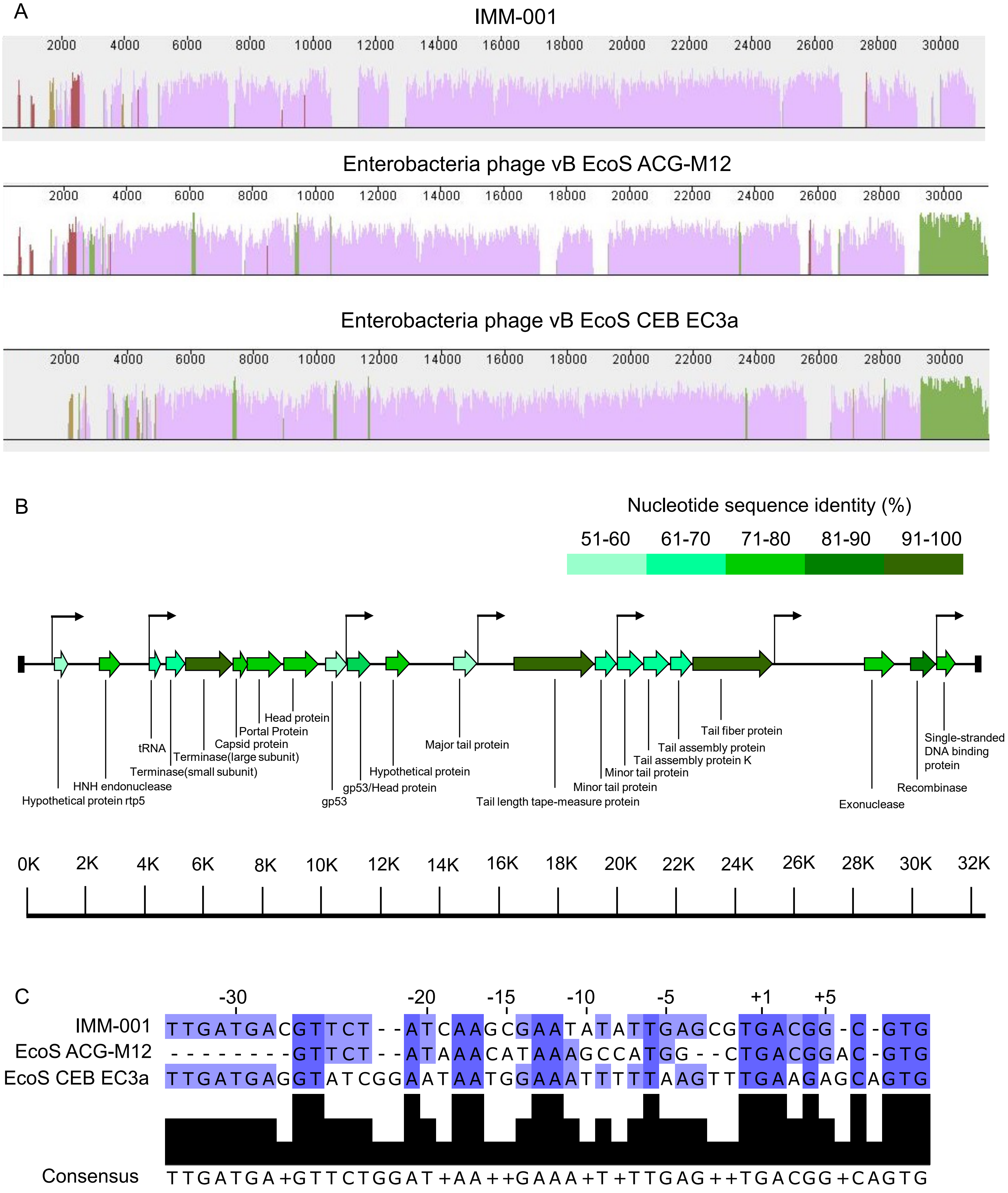
A
IMM-001
2000 4000 6000 8000 10000 12000 14000 16000 18000 20000 22000 24000 26000 28000 30000
Enterobacteria phage vB EcoS ACG-M12
2000 4000 6000 8000 10000 12000 14000 16000 18000 20000 22000 24000 26000 28000 30000
Enterobacteria phage vB EcoS CEB EC3a
2000 4000 6000 8000 10000 12000 14000 16000 18000 20000 22000 24000 26000 28000 30000
B
Nucleotide sequence identity (%)
51-60 61-70 71-80 81-90 91-100
Head protein
Portal Protein
Capsid protein
tRNA
Terminase(large subunit)
Terminase(small subunit)
HNH endonuclease
Hypothetical protein rtp5
gp53
gp53/Head protein
Hypothetical protein
Major tail protein
Tail length tape-measure protein
Minor tail protein
Minor tail protein
Tail assembly protein K
Tail assembly protein
Tail fiber protein
Exonuclease
Recombinase
Single-stranded DNA binding protein
0K 2K 4K 6K 8K 10K 12K 14K 16K 18K 20K 22K 24K 26K 28K 30K 32K
C
-30 -20 -15 -10 -5 +1 +5
IMM-001 TTGATGACGTTCT--ATCAAGCGAATATATTGAGCGTGACGG-C-GTG
EcoS ACG-M12 --------GTTCT--ATAAACATAAAGCCATGG--CTGACGGAC-GTG
EcoS CEB EC3a TTGATGAGGTATCGGAATAATGGAAATTTTTAAGTTTGAAGAGCAGTG
Consensus TTGATGA+GTTCTGGAT+AA++GAAA+T+TTGAG++TGACGG+CAGTG
