# Supplementary material for: Phenotypic and genomic analyses of bacteriophages targeting environmental and clinical CS3-expressing enterotoxigenic Escherichia coli (ETEC) strains
Source: PLoS One. 2018 Dec 20;13(12):e0209357. doi: 10.1371/journal.pone.0209357 (PMC6301781; doi:10.1371/journal.pone.0209357)

CRISPR loci identified in MG1655 *E.coli* strain

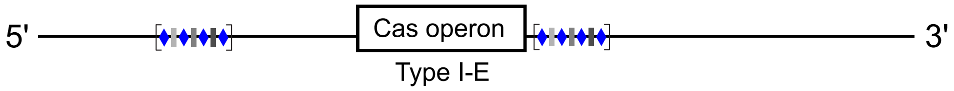

Cas operon structure identified in MG1655 *E.coli* strain

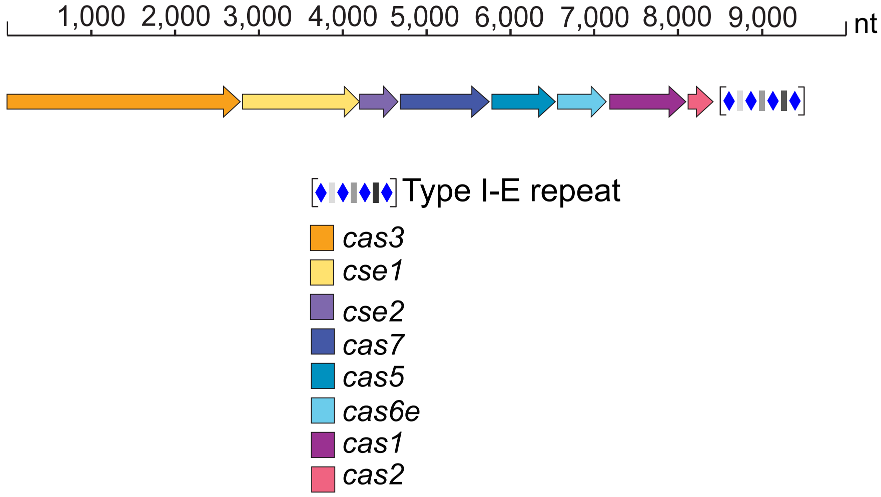

Supplement: S3 Fig — The location of CRISPR loci is indicated by “[]” where the diamond and bars represents the DR and spacer sequences, respectively. The genetic organization of the cas operon of MG1655 is depicted and color-coded according to the cas genes. (PDF) [file pone.0209357.s003.pdf]
