# Supplementary material for: Phenotypic and genomic analyses of bacteriophages targeting environmental and clinical CS3-expressing enterotoxigenic Escherichia coli (ETEC) strains
Source: PLoS One. 2018 Dec 20;13(12):e0209357. doi: 10.1371/journal.pone.0209357 (PMC6301781; doi:10.1371/journal.pone.0209357)
Supplement: S1 Table — (PDF) [file pone.0209357.s005.pdf]

Supplementary Table S1: Susceptibility of IMM-002 against ETEC and common Enterobacteriaceae

Enteric bacteria tested	No. of isolates tested	No. of isolates susceptible to phage IMM-002
ETEC (CS3 negative)[1]	112	0
ETEC (CS3 positive)[2]	83	83
E. coli (nontoxigenic)[3]	12	0
EPEC[4]	6	0
Vibrio cholerae[4]	10	0
Shigella flexneri[4]	3	0
Shigella sonnei[4]	3	0
Shigella boydii[4]	3	0
Shigella dysenteriae[4]	3	0
Salmonella typhi[4]	6	0
Salmonella paratyphi[4]	2	0
Klebseilla spp.[4]	3	0
Proteus spp.[4]	3	0

[1]ETEC strains isolated from pond, sewage and stool samples

[2]CS3-ETEC strains isolated from pond, sewage and stool samples

[3]Isolated from stool and Negative for any known toxin as screened by PCR

[4]Isolated from stool and confirmed by PCR
