# Supplementary material for: Phenotypic and genomic analyses of bacteriophages targeting environmental and clinical CS3-expressing enterotoxigenic Escherichia coli (ETEC) strains
Source: PLoS One. 2018 Dec 20;13(12):e0209357. doi: 10.1371/journal.pone.0209357 (PMC6301781; doi:10.1371/journal.pone.0209357)
Supplement: S2 Table — (PDF) [file pone.0209357.s006.pdf]

Supplementary Table S2: List of whole-genome sequenced CS3 and CS7 expressing ETEC strains

Isolate	GenBank Accession	Year of isolation	PCR Toxin Profile	PCR CF Profile	PMID
2730450	APXE00000000	2007	LT/ST	CS1+CS3+CS21	26060273
2741950	APXF00000000	2007	LT/ST	CS1+CS3+CS21	26060273
2846750	AQGG00000000	2008	LT/ST	CS2+CS3	26060273
2871950	AQDE00000000	2008	LT	CS2+CS3	26060273
2872000	AQDD00000000	2008	LT	CS2+CS3	26060273
BCE002_MS12	AQDA00000000	2003	LT/STh	CS1+CS3	26060273
BCE006_MS_23	APZM00000000	2004	LT+ST+	CS1+CS3	26060273
BCE007_MS_11	AQFJ00000000	2003	LT+ST+	CS1+CS3	26060273
BCE019_MS_13	AQCZ00000000	2003	LT+ST+	CS1+CS3	26060273
BCE032_MS_12	APXO00000000	2003	LT+ST+	CS1+CS3	26060273
E24377A	NC_009801	2012	LT/ST	CS1, CS3	26060273
B2C	AUZS0100000	1971	ST	CS2, CS3	24723709
CE549	JTGK00000000	2010	LT/ST	CS2, CS3, CS21	25932050

Isolate	GenBank Accession	Year of isolation	PCR Toxin Profile	PCR CF Profile	PMID
179550	APXB00000000	2010	LT	CS7	26060273
180200	APXC00000000	2010	LT/ST	CS7	26060273
2735000	AQGH00000000	2007	LT	CS7	26060273
2756500	AQDY00000000	2007	LT	CS7	26060273
2851500	AQDN00000000	2008	LT	CS7	26060273
2866450	AQDI00000000	2008	LT	CS7	26060273
2866550	AQDH00000000	2008	LT	CS7	26060273
2866750	AQDG00000000	2008	LT	CS7	26060273
2867750	AQDF00000000	2008	LT	CS7	26060273
2872800	AQDC00000000	2008	LT	CS7	26060273
2875000	AQDB00000000	2008	LT	CS7	26060273
Jurua_18_11	AQFB00000000	1998	LT	CS7	26060273
Jurua_20_10	AQFA00000000	1998	LT	CS7	26060273
