# Supplementary material for: Phenotypic and genomic analyses of bacteriophages targeting environmental and clinical CS3-expressing enterotoxigenic Escherichia coli (ETEC) strains
Source: PLoS One. 2018 Dec 20;13(12):e0209357. doi: 10.1371/journal.pone.0209357 (PMC6301781; doi:10.1371/journal.pone.0209357)
Supplement: S3 Table — (PDF) [file pone.0209357.s007.pdf]

Supplementary Table S3: CRISPR loci of CS3-ETEC

Isolate ID	No. of confirmed CRISPR	CRISPR ID/rank	Start position	End position	Length (bp)	DR length (bp)	DR consensus sequence	No. of spacers	Associated Type I-E Cas operon
E24377A	3	NC_009801_1	975103	975251	149	29	Type I-F repeat	2	No
		NC_009801_2	3056470	3057231	762	29	Type I-E repeat	12	Yes
		NC_009801_3	3082931	3084484	1554	29	Type I-E repeat	25	No
BCE002_MS12	2	tmp_75_Crispr_1	94671	95004	334	29	Type I-E repeat	5	Yes
		tmp_75_Crispr_2	120705	121282	578	29	Type I-E repeat	9	No
BCE007_MS_11	2	tmp_12_Crispr_2	175737	176558	822	29	Type I-E repeat	13	Yes
		tmp_12_Crispr_3	201524	202406	883	29	Type I-E repeat	14	No
BCE032_MS_12	2	tmp_79_Crispr_2	90606	91549	944	29	Type I-E repeat	15	Yes
		tmp_81_Crispr_1	10032	10914	883	29	Type I-E repeat	14	No
BCE006_MS_23	2	tmp_80_Crispr_1	59639	60582	944	29	Type I-E repeat	15	Yes
		tmp_82_Crispr_1	10098	10980	883	29	Type I-E repeat	14	No
2730450	2	tmp_151_Crispr_2	90946	91889	944	29	Type I-E repeat	15	Yes
		tmp_152_Crispr_1	9935	10817	883	29	Type I-E repeat	14	No
2741950	1	tmp_154_Crispr_1	19130	20012	883	29	Type I-E repeat	14	Yes
B2C	2	tmp_255_Crispr_1	29416	29993	578	29	Type I-E repeat	9	No
		tmp_255_Crispr_2	55543	55937	395	29	Type I-E repeat	6	Yes
CE549	2	tmp_166_Crispr_2	75442	76202	761	29	Type I-E repeat	12	Yes
		tmp_166_Crispr_3	101752	102085	334	29	Type I-E repeat	5	No
2846750	2	tmp_75_Crispr_2	91140	92266	1127	29	Type I-E repeat	18	Yes
		tmp_75_Crispr_3	117816	118149	334	29	Type I-E repeat	5	No
2871950	2	tmp_74_Crispr_2	90988	92114	1127	29	Type I-E repeat	18	Yes
		tmp_74_Crispr_3	117663	118119	457	30	Type I-E repeat	7	No
2872000	2	tmp_69_Crispr_2	90839	91965	1127	29	Type I-E repeat	18	Yes
		tmp_69_Crispr_3	117514	117970	457	30	Type I-E repeat	7	No
BCE019_MS_13	2	tmp_75_Crispr_1	91040	91983	944	29	Type I-E repeat	15	Yes
		tmp_77_Crispr_1	9990	10872	883	29	Type I-E repeat	14	No

Type I-F repeat	GTTCACTGCCGTACAGGCAGCTTAGAAA
Type I-E repeat	GWGTTCCCCGCGCCAGCGGGGATAAACCG
