# Supplementary material for: Phenotypic and genomic analyses of bacteriophages targeting environmental and clinical CS3-expressing enterotoxigenic Escherichia coli (ETEC) strains
Source: PLoS One. 2018 Dec 20;13(12):e0209357. doi: 10.1371/journal.pone.0209357 (PMC6301781; doi:10.1371/journal.pone.0209357)
Supplement: S4 Table — (PDF) [file pone.0209357.s008.pdf]

Supplementary Table S4: CRISPR loci of CS7-ETEC

Isolate ID	No. of confirmed CRISPR	CRISPR ID/rank	Start position	End position	Length(bp)	DR length	DR consensus sequence	No. of spacers	Associated Cas operon
Jurua_18_11	2	tmp_19_Crispr_1	95518	96036	519	29	Type I-E repeat	8	Type I-E Cas operon
		tmp_19_Crispr_2	122996	123329	334	29	Type I-E repeat	5	No
Jurua_20_10	4	tmp_30_Crispr_1	193224	194091	868	28	Type I-F repeat	14	Type I-F Cas operon
		tmp_30_Crispr_2	203155	203783	629	28	Type I-F repeat	10	Type I-F Cas operon
		tmp_39_Crispr_1	126608	127124	517	29	Type I-E repeat	8	Type I-E Cas operon
		tmp_39_Crispr_2	152823	153461	639	29	Type I-E repeat	10	No
2735000	4	tmp_22_Crispr_1	68332	69019	688	28	Type I-F repeat	11	Type I-F Cas operon
		tmp_22_Crispr_2	78083	78831	749	28	Type I-F repeat	12	Type I-F Cas operon
		tmp_45_Crispr_1	95151	95667	517	29	Type I-E repeat	8	Type I-E Cas operon
		tmp_45_Crispr_2	121367	122249	883	29	Type I-E repeat	14	No
2756500	4	tmp_114_Crispr_1	68386	69073	688	28	Type I-F repeat	11	Type I-F Cas operon
		tmp_114_Crispr_2	78137	78885	749	28	Type I-F repeat	12	Type I-F Cas operon
		tmp_150_Crispr_1	94542	95058	517	29	Type I-E repeat	8	Type I-E Cas operon
		tmp_150_Crispr_2	120758	121640	883	29	Type I-E repeat	14	No
2851500	4	tmp_61_Crispr_1	68325	69012	688	28	Type I-F repeat	11	Type I-F Cas operon
		tmp_61_Crispr_2	78076	78824	749	28	Type I-F repeat	12	Type I-F Cas operon
		tmp_83_Crispr_1	94541	95057	517	29	Type I-E repeat	8	Type I-E Cas operon
		tmp_83_Crispr_2	120757	121639	883	29	Type I-E repeat	14	No
2866450	4	tmp_64_Crispr_1	68443	69130	688	28	Type I-F repeat	11	Type I-F Cas operon
		tmp_64_Crispr_2	78194	78942	749	28	Type I-F repeat	12	Type I-F Cas operon
		tmp_95_Crispr_1	46877	47393	517	29	Type I-E repeat	8	Type I-E Cas operon
		tmp_95_Crispr_2	73093	73853	761	29	Type I-E repeat	12	No
2866550	4	tmp_68_Crispr_1	68516	69203	688	28	Type I-F repeat	11	Type I-F Cas operon
		tmp_68_Crispr_2	78267	79015	749	28	Type I-F repeat	12	Type I-F Cas operon
		tmp_104_Crispr_1	63867	64383	517	29	Type I-E repeat	8	Type I-E Cas operon
		tmp_104_Crispr_2	90083	90965	883	29	Type I-E repeat	14	No
2866750	4	tmp_26_Crispr_1	68495	69182	688	28	Type I-F repeat	11	Type I-F Cas operon
		tmp_26_Crispr_2	78246	78994	749	28	Type I-F repeat	12	Type I-F Cas operon
		tmp_48_Crispr_1	94699	95215	517	29	Type I-E repeat	8	Type I-E Cas operon
		tmp_48_Crispr_2	120915	121797	883	29	Type I-E repeat	14	No
2867750	4	tmp_28_Crispr_1	68427	69114	688	28	Type I-F repeat	11	Type I-F Cas operon
		tmp_28_Crispr_2	78178	78926	749	28	Type I-F repeat	12	Type I-F Cas operon
		tmp_55_Crispr_1	94647	95163	517	29	Type I-E repeat	8	Type I-E Cas operon
		tmp_55_Crispr_2	120863	121745	883	29	Type I-E repeat	14	No
2872800	4	tmp_66_Crispr_1	68533	69220	688	28	Type I-F repeat	11	Type I-F Cas operon
		tmp_66_Crispr_2	78284	79032	749	28	Type I-F repeat	12	Type I-F Cas operon
		tmp_87_Crispr_1	94590	95106	517	29	Type I-E repeat	8	Type I-E Cas operon
		tmp_87_Crispr_2	120806	121566	761	29	Type I-E repeat	14	No
2875000	4	tmp_69_Crispr_1	68420	69107	688	28	Type I-F repeat	11	Type I-F Cas operon
		tmp_69_Crispr_2	78171	78919	749	28	Type I-F repeat	12	Type I-F Cas operon
		tmp_95_Crispr_1	94877	95393	517	29	Type I-E repeat	8	Type I-E Cas operon
		tmp_95_Crispr_2	121093	121853	761	29	Type I-E repeat	12	No

179550	4	tmp_180_Crispr_1	68354	69041	688	28	Type I-F repeat	11	Type I-F Cas operon
		tmp_180_Crispr_2	78105	78974	870	28	Type I-F repeat	14	Type I-F Cas operon
		tmp_264_Crispr_1	63866	64382	517	29	Type I-E repeat	8	Type I-E Cas operon
		tmp_266_Crispr_1	137	1019	883	29	Type I-E repeat	14	No
180200	4	tmp_157_Crispr_1	9477	10164	688	28	Type I-F repeat	11	Type I-F Cas operon
		tmp_157_Crispr_2	19228	19976	749	28	Type I-F repeat	12	Type I-F Cas operon
		tmp_223_Crispr_1	64207	64723	517	29	Type I-E repeat	8	Type I-E Cas operon
		tmp_226_Crispr_1	204	1086	883	29	Type I-E repeat	14	No

Type I-F repeat	GTTCACTGCCGTACAGGCAGCTTAGAAA
Type I-E repeat	GWGTTCCCCGCGCCAGCGGGGATAAACCG
