# Supplementary material for: Phenotypic and genomic analyses of bacteriophages targeting environmental and clinical CS3-expressing enterotoxigenic Escherichia coli (ETEC) strains
Source: PLoS One. 2018 Dec 20;13(12):e0209357. doi: 10.1371/journal.pone.0209357 (PMC6301781; doi:10.1371/journal.pone.0209357)
Supplement: S5 Table — (PDF) [file pone.0209357.s009.pdf]

Table S5

Amino acid sequence comparison of Cas proteins of CS7-expressing ETEC strains with that of the reference strain E24377A

Cas protein	179550	180200	2735000	3E+06	3E+06	2866450	2866550	2866750	2867750	3E+06	3E+06	Jurua18	Jurua20
Cas3	96.9	96.9	96.9	96.9	96.9	96.9	96.9	96.9	96.9	96.9	96.9	95.2	97
Cse1	99.8	99.8	99.8	99.8	99.8	99.8	99.8	99.8	99.8	99.8	99.8	97.7	99.8
Cse2	100	100	99.4	99.4	100	100	100	100	100	100	100	100	100
Cas7	98.6	98.6	98.6	98.6	98.6	98.6	98.6	98.6	98.6	98.6	98.6	99.4	98.6
Cas5	96.7	96.7	96.7	96.7	96.7	96.7	96.7	96.7	96.7	96.7	96.7	100	96.7
Cas6	99.1	99.5	99.5	99.5	99.5	99.5	99.1	99.1	99.1	99.5	99.1	99.5	99.5
Cas1	99	99	99	99	99	99	99	99	99	99	99	99.3	99
Cas2	98.9	98.9	98.9	98.9	98.9	98.9	98.9	98.9	98.9	98.9	98.9	98.9	98.9

Amino acids sequence comparison of Cas proteins of CS7-expressing ETEC strains with that of reference strain MG1655

Cas protein	179550	180200	2735000	3E+06	3E+06	2866450	2866550	2866750	2867750	3E+06	3E+06	Jurua18	Jurua20
Cas3	29.7	29.7	29.7	29.7	29.7	29.7	29.7	29.6	29.7	29.7	29.7	30	29.7
Cse1	21.5	21.5	21.5	21.5	21.5	21.5	21.5	21.5	21.5	21.5	21.5	21.2	21.5
Cse2	22.8	22.8	22.8	22.8	22.8	22.8	22.8	22.8	22.8	22.8	22.8	22.8	22.8
Cas7	28.9	28.9	28.9	28.9	28.9	28.9	28.9	28.9	28.9	28.9	28.9	29.1	28.9
Cas5	32.4	32.4	32.4	32.4	32.4	32.4	32.4	32.4	32.4	32.4	32.4	35.2	32.4
Cas6	29.5	29.5	29.5	29.5	29.5	29.5	29.5	29.5	29.5	29.5	29.5	29.5	29.5
Cas1	83.7	83.7	83.7	83.7	83.7	83.7	83.7	83.7	83.7	83.7	83.7	83.7	83.7
Cas2	85.3	85.3	85.3	85.3	85.3	85.3	85.3	85.3	85.3	85.3	85.3	85.3	85.3
