# Supplementary material for: Phenotypic and genomic analyses of bacteriophages targeting environmental and clinical CS3-expressing enterotoxigenic Escherichia coli (ETEC) strains
Source: PLoS One. 2018 Dec 20;13(12):e0209357. doi: 10.1371/journal.pone.0209357 (PMC6301781; doi:10.1371/journal.pone.0209357)
Supplement: S1 File — (PDF) [file pone.0209357.s010.pdf]

## Supplementary GeneBank File S1: Genebank file for IMM-002_T7_phage

```
LOCUS       IMM-002_T7_phage       40060 bp    DNA     linear   PHG 10-
AUG-2017
DEFINITION  Enterobacteria phage T7 isolate IMM-002.
ACCESSION   IMM-002_T7_phage
VERSION
KEYWORDS    .
SOURCE      Enterobacteria phage T7 (T7)
  ORGANISM  Enterobacteria phage T7
            Viruses; dsDNA viruses, no RNA stage; Caudovirales;
Podoviridae;
            Autographivirinae; T7virus.
REFERENCE   1  (bases 1 to 40060)
  AUTHORS   Chakraborty,S., Begum,Y.A., Qadri,F. and Camilli,A.
  TITLE     Genomic analysis reveals CRISPR-Cas mediated host-pathogen
            interaction between enterotoxigenic Escherichia coli and
phages
  JOURNAL   Unpublished
REFERENCE   2  (bases 1 to 40060)
  AUTHORS   Chakraborty,S., Begum,Y.A., Qadri,F. and Camilli,A.
  TITLE     Direct Submission
  JOURNAL   Submitted (10-AUG-2017) Department of Biochemistry and
Molecular
            Biology, University of Dhaka, Dhaka-1000, Dhaka Dhaka-1000,
            Bangladesh
COMMENT     Bankit Comment: Vecscreen Comment:Submitter says Vecscreen
match is
            due to: This is part of Bacteriophage genome.
            Bankit Comment: TOTAL # OF SEQS:1.

            ##Assembly-Data-START##
            Assembly Method       :: Cap3 v. January 2014
            Sequencing Technology :: Illumina
            ##Assembly-Data-END##
FEATURES             Location/Qualifiers
     source          1..40060
                     /organism="Enterobacteria phage T7"
                     /mol_type="genomic DNA"
                     /isolate="IMM-002"
                     /isolation_source="Environmental water"
                     /host="Enterotoxigenic E. coli (ETEC)"
                     /specimen_voucher="icddr,b_IMM-002"
                     /db_xref="taxon:10760"
                     /country="Bangladesh"
                     /collection_date="2013"
                     /note="[cultured bacterial source]"
     CDS             205..348
                     /codon_start=1
                     /transl_table=11
                     /product="Hypothetical protein"

/translation="MSITYSQPAYSLTTYSHLPSVKPSPTANRPTVTRHYVDLQFIRV
                     LRS"
```

```
     CDS             complement(351..470)
                     /codon_start=1
                     /transl_table=11
                     /product="glycine cleavage system protein H,
partial"

/translation="MIDFKSVLWIIRVRLADTNNDNPSQLWSLTAKRTGCDNL"
     CDS             887..1339
                     /codon_start=1
                     /transl_table=11
                     /product="gp0.3"

/translation="MIYTNEPANVFYVLVSAARANLSDTVNMGRQLGLKALLQAGPEY

YGVLECTGLTGCYREEGQEVATEERTYRVRCESKAQAINVARLACNDFEQDCVMVYKS
                     QTHTAGLMFCKGWDGYKTTSLNGSFQQVDEPKGECFTKDAEGRFWEVV"
     CDS             1339..1659
                     /codon_start=1
                     /transl_table=11
                     /product="hypothetical protein"

/translation="MIAYKIFRIRKDGTLGALFINRKAVIEVGEWYEAESHPTKGFAY

RPGWHCTFEQNAPHLKLDLKSGERRAWFKVEVENITTYDRPESQGGAWVLAQRLRVLA
                     QVSE"
     CDS             1611..1691
                     /codon_start=1
                     /transl_table=11
                     /product="Hypothetical protein"
                     /translation="MGISTASAGISTGLRVINYRSSTWVA"
     CDS             1725..2459
                     /codon_start=1
                     /transl_table=11
                     /product="Phage protein kinase"

/translation="MNAHDKVIMNNLMSQLHAIICKAGYKVESLGSGIAAQDRWGAVE

VLAREHGYKLLGLGHFAAAFEHEDLPGYAIKVGFKKDDSGAAYAAFCRENEGMAGLPV

IHLVKRFSRAYMVAMDKYRSLEDIGGNYCREGKTYEQRVLNVSWRVVNAIIDQCEQPS

EALSWWLDQDARIEFAHVETQYIKDLAQTAKKINSFFYGLASFDTHRANVMVDNNGRL
                     IITDPVSWTASDHQDELQGRLNAIAE"
     CDS             2526..2720
                     /codon_start=1
                     /transl_table=11
                     /product="gp0.6"

/translation="MGWILLVIGYGLILAVITKDIIKARKVYRFQYVSLGRWTVRQPN
                     GRFMRNLANVWDIATLGSKL"
     CDS             2720..3142
                     /codon_start=1
                     /transl_table=11
                     /product="Hypothetical protein"
```

```
/translation="MCKTCAKCGETKSLDQFGSNKRNKDGLDYRCKTCRKQDAQDRRQ

KAREAGTYKTSKAEENRKLRYGLDLEEYESLLQQQGGVCAICKGQCPTGWRLAVDHSH
                     ETGKVRGLLCLQCNTALGQFKDSPELLANAIKYLQENQ"
     CDS             complement(3101..3328)
                     /codon_start=1
                     /transl_table=11
                     /product="Hypothetical protein"

/translation="MTAGLSPSSLRPEVYHRQCVVVHRRPGTSARPYCPRPGVLLAVP
                     GRAPAVDERCDVRSCACTLLILLKIFDCVCQ"
     CDS             3142..3393
                     /codon_start=1
                     /transl_table=11
                     /product="Phage protein"

/translation="MYTHKTSRHTVRQQQERDRVLQARLRAEGNKAELMCLAYGGRPR
                     TGDGKLLVSGWRAIDQRSYIKHTTKGDFSHLHANPLICK"
     CDS             3497..6178
                     /codon_start=1
                     /transl_table=11
                     /product="T7-like phage DNA-directed RNA polymerase"

/translation="MSVISIDKHDFSDVSNAIEPFNVLADHYGQDLAVKQLQLEHEAY

TEGERRFIKNLERQAERGELADNQVAKPLMQTLVPTIAKAVREWHEGPDGKSSTSRPS

VAFTMLSTDEKAVKDRSLRISAESAAVIILKVILSKLVKPEGIPITPMASAIGRTLED

EIRFGRIREQEQEHFKKTIAENLKKRAGASYKKAYMQAVEASMLEQGQLADAWGTWSP

TEAVHVGIKMLELVIQSSQLVELKRYGAGNAAADVEMVHLSDFWVKKMSQRGFSLAGI

APVYQPCVVPPKPWTGVVGGGYWAKGRRPLPLIRLGSKAAVQRYEDVYMPEVYEAVNI

IQQTPWKVNKKVLDVVNMVEKLNNTPIADIPQMEPMKPEDYAGETEEELKAWKKAAAG

IYRREKARQSRRLSLSFIVGQANKFSQFKAIWFPYNMDWRGRVYAVPMFNPQGNDMQK

GLLTLAVGKPIGADGFKWLKVHGANCAGVDKVTFEERIKWVEDNHDNILAAAKSPMDS

IDWWGQLDSPFCFLAFCFEYAGVMHHGLSYSCSLPIAFDGSCSGIQHFSAMLRDHVGG

HAVNLTPSGKVQDIYRIVSDRVEEQLKELLVNGSDNEVKTFEDKKTGEITERLVLGTR

ELARQWLTYGMSRSVTKRSVMTLAYGSKEYGFADQVFEDTVMPAIDSGKGAMFTDPSQ

ASRFMAKMIWDAVSVTVVAAVDAMKWLQGAAKLLAAEVKDKKTKEVLKPCLPVHWVTP

DGFPVWQEYRKKDTTRLNLLFLGSFNLQPTVNKGSKKELDKHKQESGISPNFVHSQDG

SHLRKTVVHTHRKYGVMSFAVIHDSFGTIPADAEFLFKGVRETMVETYRDNDVLQDFY
                     EQFADQLHESQRDKLPELPKRGKLNIEDILLSDFAFA"
```

```
     CDS             6191..6391
                     /codon_start=1
                     /transl_table=11
                     /product="Phage protein"

/translation="MKFAHKQTGVKGGTQIVTVTEHNGKGLVKTTVIPTEMSKQLNVP
                     FKWLVKLVQERHESELREVVTK"
     CDS             complement(6324..6440)
                     /codon_start=1
                     /transl_table=11
                     /product="metal resistance protein"

/translation="MYTAKKASHRASHLRSVISLPPRGVLTRVALEPASPAT"
     CDS             6388..6465
                     /codon_start=1
                     /transl_table=11
                     /product="DUF2842 domain-containing protein"
                     /translation="MTDLKWLALWLAFLAVYILIQRRRG"
     CDS             complement(6398..6520)
                     /codon_start=1
                     /transl_table=11
                     /product="hypothetical protein AK812_SmicGene8738"

/translation="MVASSAAIRAFSLRLNMVFSLFCVGLRCTLLRRPAIGPAT"
     CDS             6414..6929
                     /codon_start=1
                     /transl_table=11
                     /product="gp1.05"

/translation="MAGLLSSVHLNPTQKRLNTMFNRKLKARIAALEATIQRQDHNLE

LANREQARLRAKLQKAETKREVEPLRVKRMVGLKFKALFNGIYQPTEFKTGPGPCGKT

VTHFTTSRDERTLTIHQHHTDESSKSFEYRLSDIDGRIQYDYELVQVYGEEAERERMV
                     QALRNRPRFIA"
     CDS             6981..7073
                     /codon_start=1
                     /transl_table=11
                     /product="Uncharacterised protein"
                     /translation="MINPVAKLLPNSSNQELHHVSDHYQLRAHP"
     CDS             7036..7215
                     /codon_start=1
                     /transl_table=11
                     /product="Phage protein"

/translation="MYQTIINFERIRERQQTEGYIPKGRKLNKTKRGGGVKGAFRNAE
                     GKDSLVNQEKYFVGA"
     CDS             7218..7466
                     /codon_start=1
                     /transl_table=11
                     /product="transporter, partial"

/translation="MSRRWLFDASTSQWSSLGRTHREMGESGLEVRMDELPCSRTTLV
                     RITEADGKPLVSREFTEHEIFSCEQWCLQVLNDYRSWK"
```

```
     CDS             7466..8551
                     /codon_start=1
                     /transl_table=11
                     /product="DNA ligase, phage-associated"

/translation="MTTTLKTNPHRAVDYSESGVKKALDAAGSLEAEVKYDGVRLNIP

VLREGETYWLSRESKPLPALEWMNSELGNAWTQADWRWFLRQAGYEGVGLMIDGEVMV

KGVDFNTSSGLIRTKWLKPNNEAFAECYPGRGKKVPFWVARSRLQVVVYGVVDMTTIA

DPKAEGPIHSVTRLKAEAIVPLLQKYFPEIDWVLSESHTVFDLESLNSLYEEKRLEGH

EGLVIKDPLGNYKRGKKSGMWKMKPEDTIDGTVCGLVWGTQGKANEGKVIGFEVLLED

GMVVNACGLTEEQKDEFTAKVKEDTLAAVGIFAPYGIGGNEPCFDNPYEGWQVEVLFM
                     ERFPDGSLRHPSFNQWRGTEDNPTIKS"
     CDS             8337..8555
                     /codon_start=1
                     /transl_table=11
                     /product="major fimbrial subuni"

/translation="MSSPPRLKRTLSPLWAYLLRMVLAVTNPASITHTKAGRLKSSSW
                     SASRTAPCATQVSTSGVARKTIRPLRAN"
     CDS             8674..8928
                     /codon_start=1
                     /transl_table=11
                     /product="gp1.6"

/translation="MSKNLMFNRFSQTFHLSHNPFACIKRNEKVGFFGKAVKLAPTVY
                     ALIVPSKVEEARQKKETSVPVVYTKWPRVRLVLEFIKEEL"
     CDS             8928..9305
                     /codon_start=1
                     /transl_table=11
                     /product="gp1.65"

/translation="MAHYCVDCQNDHTECTCHLKYQTPAPSCSPTYTGGSADYYQVPI

TKTTTPGRPDYIAECNDIIEALGMNFAEGNAFKAIWRRAAQRTLGLRKAGAKDDGLYD
                     AEKVEFFGARLVAQSKAAGHDSK"
     CDS             complement(9290..9391)
                     /codon_start=1
                     /transl_table=11
                     /product="hypothetical protein SAMN05444584_0310"
                     /translation="MVIVLSLSRGYTFRDSRRYPSSQTSSRTIHLES"
     CDS             9384..9560
                     /codon_start=1
                     /transl_table=11
                     /product="PTS sucrose transporter subunit IIABC"

/translation="MTIYKGIFGSLYAVNQHGVVCVKPDGGEWCASHYSVEGFQDAID
                     KGYVTEVVDDQGVN"
     CDS             9541..9711
                     /codon_start=1
```

/transl_table=11
/product="Host RNA polymerase inhibitor, T7-like
gp2"

/translation="MTKESTEKKYIVELEGRVQSFDVPVYAKSLDEATLKAQEYEDAG
FVVGRIRPELHD"
CDS 9757..10455
/codon_start=1
/transl_table=11
/product="helix-destabilizing protein"

/translation="MAKEQLKTFTTPVAGLVEPYSWLNKSDTKFNERGEHKVNLTFDL

SDPKVRKMIDVLQKIHDDAYAKALADHEKNPPQVQRGKKPIEPREGDMPWIENGDGTV

TLKFKCFASYLKDGKSEPIVLRFYDTDAKLIRDVPNIGTGSKLKVKFKVLPFKWNAAT

GASVKLQLESVLLVELKEWSGDGTGGDGGWGDDEDIGSGGYKASTDGDFGSDDFGEDA
DGGDDSASGGDYDF"
CDS complement(10123..10467)
/codon_start=1
/transl_table=11
/product="Hypothetical protein"

/translation="MQPRLEVVVATGRRVIATIGIFTEVIRPEVTVSRRFVATGADVF

IVTPATITTSTIAGPLFEFDQQNGFELQLNACTSRSVPLERQDFELDFQLRPGANIRD
VTDEFSIGVVEP"
CDS 10301..10516
/codon_start=1
/transl_table=11
/product="gp2.55 protein"

/translation="MVLVVMVAGVTMKTSAPVATKRLLTVTSGLMTSVKMPMVAMTLR
PVATTTSNRGCMDTKTGALCGCLPLWT"
CDS complement(10452..10691)
/codon_start=1
/transl_table=11
/product="acyl carrier protein"

/translation="MLLAVLTVEDTFGLDDDAIRKDEVWCILVVGSRNHIVDIMAFVL
RFDAVLFQPLLVLGLKSRAVGTHRVPPFWCPCSHD"
CDS complement(10583..10918)
/codon_start=1
/transl_table=11
/product="Hypothetical protein"

/translation="MLSHFLAPFFGMSIPEGRRTVASFSHSTGINLSANLKPFFSHHA

PYVVGDPLYSLEREDENTNRMSSSGCCSRTSICFLRSSLSKIPLVSTMMPFGRMKSGV
YLWSEAGIT"
CDS 10907..11356
/codon_start=1
/transl_table=11

/product="gp3.2 protein"

/translation="MTKHLTCVDLPFGAQYKFHPDVIEAHPGLSTDTYERRDNHSVVV

TGPFKGNTVQWYGHEKTRIVEVGKPQVLPASSVPPVTVKREVLTIDKIGVGQTFIVHG
KPDEVYVKISNSHVFNHKRLQMHTTVAERFQSHLNLVVVELVVYNGK"
CDS 11346..11804
/codon_start=1
/transl_table=11
/product="lysozyme"

/translation="MASKVQFKPRTVTDAIFVHCSATQPSQDIGVDTIRMWHKQQGWL

DVGYHFVIKRDGTVESGRPVDVVGSHVKDWNSRSVGVCLVGGIDAKGKFEANFTPAQM
SALRNKLAELKVLYPQADIKAHHDVAPKACPSFDLQRWLNTNEMVTSDRG"
CDS 11819..12031
/codon_start=1
/transl_table=11
/product="gp3.7"

/translation="MIKFIEFLGRLVVRGYSRAASVERKVASAAAKGAEAAAAEADSL
RIKSLDAGLRARGLDKKAEQLKGFFS"
CDS 12100..13803
/codon_start=1
/transl_table=11
/product="T7-like phage primase/helicase protein"

/translation="MSYEDQEQDDSVFLYHTECPDCGSSDANGVYSDGHMYCFACDPS

VAWKKGDMELTEGYTPSGGKKQVSNLLTWGENSGRYVPLPARGLSADICKKYSYWVGM

MQGKMVQIADYYDRSGTKVGQKVRDADKNFTAIGSVKNDLLFGSQLWSGGKKIVITEG

EIDCLSVAQVQEGKYPVVSLPLGAKSAKKTLAANLEYLDQFEEIILMFDMDEPGREAV

EQGAPVLPAGKVKVAFINGYKDANAALQAKDFRAIQDAIWNAVPFVPAGVVSAKSLKD

RTREAMLKAETEGLLFSSCKTLNAMTLGARAGELIMVTSGSGMGKSTFVRQLLLEWGR

IGKRVGMAMLEEAVEETVQDLMGLDNNVRLRQCADLKEAILKDGRFDEWYDKLFNDDK

FHLYDSFAESEEDTLFAKLAYMVDGLDCDVILLDHISIVVSGMEDNSDERKTIDRIMT

RLKKFAKTKGVVVVVICHLKNPEKGKSHEEGRPVSITDLRGSGALRQLSDTIIALERN

QQGDTPNVVQLRLLKCRFTGDTGIAGHLEYNKLTGWLEPISAPSGGGEEDSSSWENQD
F"
CDS 13532..13855
/codon_start=1
/transl_table=11
/product="gp4.2 protein"

/translation="MKKVVQLVSLTCVGLVLYVSYLIPSLPWREISKVILLTLFSSVC

```
LSVALLAIQALRDILNTTSSLAGSNRLAHLAEAERKIAARGKTKTSKCQCPECDWKSP
                     YGGHH"
     CDS             complement(13800..13991)
                     /codon_start=1
                     /transl_table=11
                     /product="cyclic nucleotide-gated channel rod
                     photoreceptor subunit alpha-like"

/translation="MNFWFARFHALESNRWSPRIRDRQLASSFHHLYMNVWYRALSFL
                     NTNGVLHMVTSSRTQGIDI"
     CDS             complement(13852..14049)
                     /codon_start=1
                     /transl_table=11
                     /product="hypothetical protein"

/translation="MTLSLSYSDSLVVDTQEVIHELLVRSLPRLRVKPLVSKDSGQTV
                     GLLVPPLVHERVVSGFKFLKH"
     CDS             complement(14046..14291)
                     /codon_start=1
                     /transl_table=11
                     /product="hypothetical protein"

/translation="MRCGCNLFDLLHILFEATDDTYHRSYPILQGLIRLGVNQDPTVE
                     LRHGIKKTVGLDVRTPDVLQVNHNVSYSLIIKATRSE"
     CDS             14091..16262
                     /codon_start=1
                     /transl_table=11
                     /product="T7-like phage DNA Polymerase"

/translation="MIDLQNIWGSDIETNGLLDAVSQFHCGVLINAESNETLKYGVAP

MVGIVGGFKEYVQKVEEIAATPHGLLVFHNGINYDVPAIDILKRKYFGKRFNFPKQKM

IDTLVMGRLMYPNIKFSDIGAVKAGRLPPKMMGRQSLEAWGYRLGEMKGEYKTDYIAK

CRTEGIEYKAGDEWLFPSQEMLDYNVQDVVVTLALFKKFLTDKFYFGTPEAGLEAVYA

LRLEHDAAWTCAKMERNGYPMNSEIVEGLYRELSIKRAELLDKLRSTFGSWYSPKGGK

EFFRHPRSGVDLPKYPRVIYPKVGSIFKKPKNKAQRLGLEPCERDSRDTMEGAPFTPI

SFVEFNPGSGDHLAKVLMDRGWEPTEFTETGKPKCDDEVLENVHLADPEAQACVELVR

EYLVVQKRIGQAAEGKNAWLRLVGPDGRMHGSINPCGAVTGRATHSSPNMAQVPANGA

PWGEICRSAFGAQWNQNAGKPDPWIQVGVDASGLELRCLGNRASPFDGGEYAKTVVEG

DIHWANAVNAGLAPNVPRDKSSHDHDAFRNNAKTFIYAFLYGAGAAKIGLIVGGGKKE

GAALMKKFIEGTPAIKDLREAVQNTLISESKWVDGENIVKWKRRWLKGLDGRRIHIRS

PHSALNALLQGDGAVVCKHWIVETERMLEEAGYVHGWEGDFAYMAWVHDELQIAARTV
                     EIAEDIRRIAQLAMRKVGEFYNFKCVLDTEGKIGPTWKECH"
```

```
     CDS             16060..16275
                     /codon_start=1
                     /transl_table=11
                     /product="hypothetical protein JL11_08760"

/translation="MSTDGKGTLRTWHGYTTNCRLRLGPWRLQKIYVGLLNLLCVRWV
                     SSITLNASLIPKVRLDQRGRSVTSGYY"
     CDS             complement(16204..16470)
                     /codon_start=1
                     /transl_table=11
                     /product="hypothetical protein"

/translation="MDFLKASSRAASGPDSAASLIRANWMPSSFSPSVNILDSFAKLR
                     QEISSPLEHTTSLRSKLTRIRLVIATSDTPSTLVQSYLRYQGRI"
     CDS             16364..16546
                     /codon_start=1
                     /transl_table=11
                     /product="HNS binding protein"

/translation="MFTEGEKLDGIQLALIKEAAESGPEAALELAFKKSIKESVVEAL
                     DDEPFTASNFRFEVKR"
     CDS             16500..16637
                     /codon_start=1
                     /transl_table=11
                     /product="hypothetical protein"

/translation="MSPSLLQTSASRLNDERIPAGPCGPQVLPEDLSVQLCAQQRCTS
                     G"
     CDS             16543..16752
                     /codon_start=1
                     /transl_table=11
                     /product="conserved hypotetical phage protein"

/translation="MSEYLRVLAALKSCPKTFQSNYVRNNAALVAEAASRGHLSCLSM
                     DGRNNGAWEITAAGTKFLNQHGGCL"
     CDS             16736..16816
                     /codon_start=1
                     /transl_table=11
                     /product="hypothetical protein V12B01_13545"
                     /translation="MEAVYESTTFGLSGSTYRSWRQVRLR"
     CDS             16749..17024
                     /codon_start=1
                     /transl_table=11
                     /product="hypothetical protein sh4_0026"

/translation="MSQQPLDFLGQPIEVGDKFVYAEAGGRGGTMALHSGVVTRMTEK
                     QVLVEKTRWGQTWRPFNCIVIVEKCGPDEEPLLGATLAMARGGLRDE"
     CDS             complement(16989..17153)
                     /codon_start=1
                     /transl_table=11
                     /product="hypothetical protein"

/translation="MRLAFSWSQISVQMSSPQSVSSSAAAIEEKTRKSPSSTRAIFLS
                     LIPETSSSHS"
```

```
     CDS             17017..17883
                     /codon_start=1
                     /transl_table=11
                     /product="exonuclease"

/translation="MSDKKIALVLDGDFLVFSSMAAAEDETDWGDDIWTLICDHEKAK

RILMNTISEIVKKRKAWKDAKIVMCFTDDVNWRKSILPTYKANRKGSRKPVGYKKFVA

DIMADPQFNSFLRSTLEGDDCMGIIGTRPQIVGCDHAVLVSCDKDFKTIPNCEFYWLT

TGEILSHTTAEADYWHMEQTIKGDMTDGYGGIPGYGEDTTRAFLDEPYYFVQETRELK

TGKNKGQLKVEWKKYPKREDQTLWDCMVTLAAKAGMTEEELLVQAQVARICRASDYDP
                     KSKEVILWTPYI"
     CDS             complement(17880..17993)
                     /codon_start=1
                     /transl_table=11
                     /product="S26-RNase, partial"
                     /translation="MASRTIGYDYRLDSVLRSVRPTDCVHRVKGQKSGPVD"
     CDS             18092..18364
                     /codon_start=1
                     /transl_table=11
                     /product="Phage protein"

/translation="MGIKAIDDVVNQLVEERLKVPAFSESAVQFLHILFNASYASKLG
                     VINDLKAAGHSEGYIAGFIGGLQYCSDTLDSAVAKRQSLKDNIQFD"
     CDS             complement(18299..18589)
                     /codon_start=1
                     /transl_table=11
                     /product="related to allantoate permease"

/translation="MLTPVLRIARPTDDLVAVDSFLTFRSLIPLVSLVSAPSSAPMST

LATSSLRGAGSGAAVDGFGVLIFGLKHIVPPLVELNVVLQALTLSDRRVQSV"
     CDS             18375..18596
                     /codon_start=1
                     /transl_table=11
                     /product="gp6.7"

/translation="MCFSPKISTPKPSTAAPEPAPLSEEVASVDIGAEEGADTNETKG
                     IKDLKVKKESTATKSSVGRAMRNTGVNMG"
     CDS             complement(18599..18685)
                     /codon_start=1
                     /transl_table=11
                     /product="hypothetical protein X743_33460"
                     /translation="MRRSQTVRLPRGVQQPCGGHRLSFGSGA"
     CDS             18601..19002
                     /codon_start=1
                     /transl_table=11
                     /product="gp6.8 protein"

/translation="MLHYRNSSDGRRMAAERLWEDGQSDFASFSEFKQHTFRMADELE
```

GEEYTIYDDECKPVAYLYMLASSSWHRKTPGLDLSILAIRSDSQSSRKVLETVRHIID
GECRRWGLNWWSRVKHNGPVDIVTTKEINRG"
CDS complement(18796..19239)
/codon_start=1
/transl_table=11
/product="hypothetical protein"

/translation="MPPPEVRETLRLRLPTRLPFFEPSAVTSVSTSVTVSFGTSTGAA

ATCWSAALGTEAAEGPRPTVFNAPPTTFLNALLMDLPTVNLLGCNDVYWPIVLDTGPP
VQTPSSALSVNNVSDSLKNLAGGLRVTTDSENGKVKTGSLSVPRR"
CDS 19263..20831
/codon_start=1
/transl_table=11
/product="head to tail connector"

/translation="MAERQGFAAEGAKAVYDRLKNGRQPYETRAQNCAAVTIPSLFPK

ESDNSSTEYTTPWQAVGARCLNNLAAKLMLALFPQAPWMRLTVSEYEAKTLSQDTEAA

ARVDEGLAMVERVLMAYMETNSFRVPLFEALKQLIVSGNCLLYIPPPEQGQYSPMRMY

RLVSYVVQRDAFGNVLQIVTLDRIAFSALPEDVQSQLNADDYEPDTELDVYTHIYREG

DEYLRYEEVEGVEVNGTDGSYPLDACPYIPVRMVRLDGEDYGRSYAEEYLGDLNSLET

ITEAITKMAKVAAKVVGLVNPNGITQPRRLNKAATGEFVAGRIEDINFLQLTKGQDFT

IAKSVADAIEQRLGWAFLLNSAVQRNAERVTAEEIRYVAGELEATLGGVYSVQSQEMQ

LPLVRVLLNQLQSAGMIPDLPKEAVEPTVSTGLEALGRGQDLEKLTQAVNMMTGLQPL

AQDPDINLPTLKLRLLNALGIDTAGLLLTQDEKLKRMAEQSSQQAVVQGAGAAGANMG
AAVGQGAGEDMAQA"
CDS 20901..21776
/codon_start=1
/transl_table=11
/product="Phage capsid and scaffold"

/translation="MSQSVYAEFGVSPNAITGSVEDLNEHQQSMLEKDVAVRDGDDAI

TFKQLEAEQEEATEEDENVEETEEEEEVEETEDSEGQDQEFIELGDTPKELTESVSAL

DENEAAFDDMVSAAVEAGKVTADDIATIKAEYAANGELSEASYAKLAEAGYTKRFVDS

FVRGQEALAEQYAAGVIRYAGGAEQFNRILSHLEANDKSTKEALESAIIRKDLVTAKA

ILNLAGRNLGKARGVQPQRTITTQGKPAVSAPKVETEGFSSKADMVKAMSDPRYLRDA
KYTMDVRAKVAASSL"
CDS complement(21884..22051)
/codon_start=1
/transl_table=11
/product="hypothetical protein"

/translation="MLVSSNGTTSESGQDFTSEDLQEQCELVGIGIAFTLVLTNFLTR
YVCHMIILLFE"
CDS 21908..22957
/codon_start=1
/transl_table=11
/product="major capsid protein"

/translation="MANVPGQKIGQNQGKGNTNTDQLALFLKVFAGEVLTAFTRRSVT

ADKHIVRTIQNGKSAQFPVMGRTSGVYLAPGERLSDKRKGIKHTEKVITIDGLLTADV

MIFDIEDAMNHYDVAGEYSNQLGEALAIAADGAVLAEMAILCNLPAASDENIAGLGKA

SVLEVGTKADLNTPAKLGEAIIGQLTIARARLTSNYVPAGDRYFYTTPDNYSAILAAL

MPNAANYAALIDPETGNIRNVMGFVVVEVPHLTQGGAGETRGDDGISIASGQKHAFPA

TSSTTVKVALDNVVGLFSHRSAVGTVKLRDLALERDRDVDAQGDLIVGKYAMGHGGLR
PEAAGALVFTAASAG"
CDS complement(23050..23280)
/codon_start=1
/transl_table=11
/product="transcriptional regulator, XRE family"

/translation="MSPPLNEKPLKHPKAPKGFQLVCERYVEGTHRRTVIPNNHQTFH
ARGGLEVETVGSTGVTNTIRRSHLKQRPIGNV"
CDS 23279..23845
/codon_start=1
/transl_table=11
/product="phage tail protein"

/translation="MAVYIPLNTNDDLDAINDMLAAIGEPAVLQLDEGNADVSNAMRI

LQRVNRQVQAKGWNFNINEAAVLTPDISDNQIRWLPSYLRVMTTGATSYYSNMGGYVY

DLSTQSTTFTAAITVELVELKPYGEMPVVFRDYIVTKASREFNAKFFGSPEAEMYLRE
QEAELYQQVMEYEMDTGRYNMMSSIGRD"
CDS complement(23600..23920)
/codon_start=1
/transl_table=11
/product="hypothetical protein"

/translation="MSGCWLMPPLRFFIDCVISGIAPPLLVSTDGRHHVVATSVHLVF

HHLLIQLCFLLTEIHLSLRATEELSVELSACLGHDIVPEDNRHLTIGFQLYQLDCDCS
CEGC"
CDS 23859..26213
/codon_start=1
/transl_table=11
/product="gp12"

/translation="MPLITQSIKNLKGGISQQPDILRFSDQGEEQVNCWSSESDGLQK

RPPTVWKKRLQIDVGSAPKFHLINRDEVEQYYIVFNGSNIQVVDLEGNPYAVSGDMGY

VQTSNPRDDIRVITVADYTFIVNRKMVVRAGTDKSHPGYNTKNRALINLRGGQYGRTL

KVGINGGVKVEHKLPNGANAEEDPPKVDAQAIGAALRDLLVAAYPNYTLALGSGYLTI

TAPVGTEITSVETEDGYANQLINPVLDTVQTVSKLPLAAPNGYIIKIQGETNSSADEY

FVVYDSNTKTWKETVEPGTVIGFDNQSMPHALVRQADGSFDFKEMEWSGRGAGNDDTN

PMPSFVDSTINDVFFYRNRLGFLSGENVIMSRSAGYFAFFPKSVATLSDDDPIDVAVS

HPRISILKYAVPFSEQLLLWSDEVQFVMTSSGVLTAKSIQLDVGSEFALGDNARPFAV

GRSVFFSAPRGSFTSINRYFAVADVTDVKDADDTTGHVLSYIPNGVFDIQGTGTENFI

VVNTSGAYNRMYIYKFLFRDGVQLQASWSHWEFEPNERVLASAAIGSTVYIVKQHSQG

VDIEHLKFIKEATDFRTEPYRLHVDSKATMQIPTDSYNPETFVTLVNIGNAYGGNAPP

RGNYFLIDSQGAYTSLGLLDGQTLVNLKGDWSGREVFIGRSYNMRYRFSRFLIKKEDD

SGVQTEDTGRLQLRRAWVNYQDTGALRLTVSNGQREFVNILNGYTLGQQTLGTSNIGD
GQFRFAMNGNAMTTSLVLESDYPTPVSIVGCGWEASYAKKAQRV"
CDS 26300..26770
/codon_start=1
/transl_table=11
/product="internal virion protein"

/translation="MYIRKATESDVHYFLWHLSADDVNECKANYGSTVGLSERLLKHL

SPSSVVLTNGVGEVFAYGGNQGDNVWFLTSGQVYKLRPKEKREFIKRISEYRDLMLDQ

YGTIWNYVWSGNKSHIKFLKLLGAKFHPEVTISPVTGERFQLFTISKEDVCANP"
CDS 26755..27342
/codon_start=1
/transl_table=11
/product="gp14"

/translation="MCEPVSIGMGIMAVAGAAMSASSQAKAEGAAIDAQNRQAQEMIK

QMNYSDVNLKMQERDLKEQQVAELTETTLNGIRNQGMVRAAVAESGLEGNSMDRIERD

VEGQTVKERAGITESYGRDYAAIFGNRISNIENTKSAIKGQGKILKTSPLAHALNVAN
AGMQGYAMGNSIAGASPSQGAAPISAAKGTPTGHS"
CDS 27107..27361
/codon_start=1
/transl_table=11
/product="hypothetical protein"

/translation="MAVTMRLSSGTVFPTLRIPSPLSKVRVRFSRPAHWLMRLMWRTP
GCKGTQWVTQSLVPLRLRAQPQLVLLKAHLQVIANRRTNG"
CDS 27354..29636

/codon_start=1
/transl_table=11
/product="Phage protein inside capsid C"

/translation="MASNIESALANRTMGRGRAPGKAISLDYQAANVQGQTGDSGLAK

ALTDFVSTGAGVYKQFQEKAKSLGDERSNEIIRKLTPQQRRDAIHNGTLLYQDDPYAM

EALRVKTGRNAAYAVDDEINVKIQNGEFRTRQDMEEYRHQRLQDAAKSYAEEAGINPA

DEHFQRGFNADITDRNVAIYGSFNKYFSKQSENTAMLNTRVELNSFLNDGDLMRSPEA

GKSFMAYMRDGLTTAAIPSDQRATEVITQTVRDAIQKSGGSNFLQQVRNERITLNGVD

ATVEEIVGPEVFNAAMIEAQGTEYKLVAKYQEDLSLGVQSAMLQDDPTIGLAQIQKLK

AQNNQLQPGEEMTPQRQMLINAEASLLESVKRKSAEQAKENTKLIQTQNKQLVIDQVY

QRRLNGDNVSTNYEDLPVSEATGEFKRSDMNNYAMGKLQQIDQMDIPQAAKDAQKVAL

LRADTNNGPFRNAFQTLTQDAAGEWQAAVIRGQYDPDKMQRFESLRKVYTQDPSSFAA

LYPDQASLFTTFEQMDKMGLDPQTMIDADKQAASQSREMRIESDKAWQELKNDSKNKD

LSRLPTSLDASARKVWDSWYYRTGNADAATQNTQKWLNENTVTFSDEGQDGKSIGMVS

KHQLMVGDNPESWQVGRDIIDTARQQLIKTNPWVVNSQLSVVEQNGSVFLQDATGTIR

IRYDKELVGKLYREQQKQAEDKAYAEAERKANTRARIVETKAAGDKRRNEREANIQKR
GGMYKDVSLEGIANTLIGKE"
CDS complement(29557..29682)
/codon_start=1
/transl_table=11
/product="hypothetical protein"

/translation="MFPMLLLRIPRVAIVITPYRLRCLRYPPAIRLCTYRPVSGY"
CDS 29642..33532
/codon_start=1
/transl_table=11
/product="internal virion protein D"

/translation="MATRGIRNNNIGNIRVSKDQWEGATGDDGSFVTFDTPDSGVRAL

AKNLMSYGRQGYDSIEKIITRWAPPSENDTQSYIQSVASATGIPATQSLDLTDPDVLA

SLSEAIGYHETGSRYDKSVYQTGVGRALGNGITPKSPPVSANVFDALTEGLKAKPKVA

LGENLPGVTGLNIEGQEPEAPNESFGEMFYKSTGETLDQRADRSTWFGFGGAAEAEVN

NSMVGVAIRAGQTEDSLDVIGDVFNPTRWNNHKWSREELDQIRNAGVLPQYYGVITGG

SPQNLTELINLALENQKLDAEKAKAGTGAQLAAGVIGAGVDPLTYVPIAGQVGKGGKL

INKMFTVAAQSGALAGASELARTSVAGGDAHVAEAIMGGALFGGGMTAIGDAIGKALG

KSTNEFAGPATRLEARETARNVDGQDLSRLPIREGEETFSHQGVKFADVPNEPGSVRL

EDGSILIGENPLNPKTRQVFDEVIEPERAAAGVNLGGLTEIGLKLLRSENPEIRGLAA

DLVRSPTGMQSGASGKIGTTASDVFERLRAVDHRFYNDIDDAVTQALKDPYFQTNFNR

DTGAFRQDIYQRVALAIEDGSGNLKAELTPGELKVYGLLKNQFDAKREMMENPAMFGR

VDAQSIFPGSRFKGTYVPHVYSNQMKQLFINELGSPEALQEAIKKSWLTSYASRPEVK

ARVDEALLEANPNLKPEELAAAVDKYANDKAYGISHTDLFERSSVMEENINGLVGLEN

NNFLEARNLFDSDMSIILPNGQPFNVNSLREWDMDKIVPAYNRRVNGDIAIMAGTGKT

TKEMKDTVETMMNRAGDDGKLKGEVATLRDTLKILTGRARRDGADDAAFSTVMRTMTD

LSFFAKNAYMGVQNLTEIGGMLARGNVRAMLHGIPMFRDLAFRNKKMGASEIKDLHNV

VFGKELDDSIRPSKQDVIDRLRAYSDLSKPVATALGSAKYYTGELAVRSPFTKVLNGT

TNYLLDAGRQGFLSDIVEHSLTGSKRKFDDRWLKTAGISADQWKGIKSLIRESVTRGP

DGKYTIKDKKAFSQDPRAMDLWRMGDTIADETLLRPHKLSNMDAKAYGPLAKTVLQFK

NFVIKSINGRTMRTFYNATKNNRAMDAALSTVMSMGLAGMYYMAQAHIKAYAMQDGRD

REYLKQALNPTMIGYAALSRSSHLGGPLGVANILGGIAGYEDTKLLRSSILPRSPTEK

PERAITYGAAKSDPVMNVVGNFLEQVPAFGYAANVGASAYNLAGYLKSDTRVNERDYM
TGMYNTFRELVPNDPITQKLLLGTFEEQGIHIKN"

```
     CDS             33592..36390
                     /codon_start=1
                     /transl_table=11
                     /product="Phage tail fiber"
```

/translation="MAKTTITQFPAGQSQYRIEFDYLARPFVVVTLVNSADQTQNRVL

RAGADYRFLNPTLIEVMIPQTGFDTLQIHRQTDTELIVGFRDGSVLTAKDLTNAELQA

IHISEEGRDQTVDLAKEYADAAAKARNDAEDARDSIEQVMKSGLYGYTLVEDFQKGAT

LSHPAEALRWTLPDGTGEYYRWDGVFPKVVPAGSTPTSAGGVGVGAWVSIGDASLRGN

LANPAYGDGLVASQLPNSPVARTVHDKMLEAVSIADYMVGGDVAGAIIGALSSTAGRV

SVPAGNHIATPSAAQVAGVLSALSRLNINGSLTIRLPKGRVNLSSPVLVELDGGNNLS

IEGQPNVPVTITGQASVSGSAGNYQVTLNVSSTAGVSVGDFLHTNQATGTGACDLHRG

VWEITAVGAGSLTVRNTCQLSSFPANTITSSSSRVLTSVLMFDRCDGFIVPSSEVGNM

SNFVIAGNSDSYWRASAVGTTELGTHGLAVGSNTVAVNGKSDNVNPQGKTGGSVTFGQ

YMGVSGFDQQGIVTELGGSFWGDFTCCCNNKRRGFYSSTASGIRAKQITANGNYLDGV

IADIGGDIYSSSSSCAAGNGSSGISAAHNGSVIWDAGKASYNKLNGANGVAGGFLQMT

GSTMQGNLATGANLAYGAILYCDNSQISLNGTYGINCQLGSVVRGPNCTYIGNNNQGI

RGSYNATVTFTGSTFSGNSGGDFLFTAMSLGIFGNTNYGGDIVATDIKLVNQSTGKGV

RLTGTSGGDNIVMSYDVTGNGSFVEGYNFRSGDVGIYPSDDAVRNIGRPANRFNIGFF

AGGTQSTSDARLKDPIRDFSEAELKAAVACSKSLGFWTWLDDDSKRLHAGTTVQRVLE

ILEDNGLDWREYGFIGFDSWEDEYKPVVAEIDGMEYETGEVVKVVEAGSLWQLRDQEF
DRFLIRGLSARLSAIEKD"

```
     CDS             36445..36639
                     /codon_start=1
                     /transl_table=11
                     /product="Phage holin, class II #T7-like gp17.5"
```

/translation="MIEFDFKNEVLKASPIVGTAAADGASRFFFGLTLNEWFYVAAIA
YTVVQIGVLIYKTIKSGGKT"

```
     CDS             complement(36617..36697)
                     /codon_start=1
                     /transl_table=11
                     /product="hypothetical protein"
                     /translation="MSRARSVSSINRNFSKSICVMSYLRS"
     CDS             36636..36899
                     /codon_start=1
                     /transl_table=11
                     /product="DNA packaging protein, small subunit"
```

/translation="MTQMDLEKFLLMLDTERARLMLQDLRDDSKRSPQLYNAIEKLLA
RHNFVLSKVSVDEKQLADMEALNREYDKVLSAAEDNDTGYGVQ"

```
     CDS             complement(36721..36918)
                     /codon_start=1
                     /transl_table=11
                     /product="hypothetical protein"
```

/translation="MTLSLTLIGHHTRCHYLRQLIAPCRTLCSEPPCQPTASRPPTPC
STQSCDGPTASRWRCKAGASA"

```
     CDS             37002..37451
                     /codon_start=1
                     /transl_table=11
                     /product="conserved hypotethical phage protein"
```

/translation="MLKLLRQAVPWLVAGLLFCSGYWVADNKWEAKVNNEYITKLEAT

EQSRRSVQSEVNKVSAEWQDKMSELEGSTDRVIADLNRDNKRLRVKVNTTGITESDYS
RCFPDGRVELHPETSKSLIRITQEADLKEKALQDTIRKLQQEKEAKP"

```
     CDS             37481..39211
                     /codon_start=1
                     /transl_table=11
                     /product="Phage DNA packaging"
```

/translation="MTARMKADFVFFLFVLWKALSLPVPTRCQIDMAKKLSAGDNRRF

ILQAFRGIGKSFITCAFVVWKLWNNPDLKFMIVSASKERADANSIFIKRIIDLMPQLQ

ELKPKQGQRDAVISFDVGPAKPDHSPSVKSVGITGQLTGSRADILIADDVEVPGNSAT

QAARDRLSELVKEFDAILKPGGTVIYLGTPQTEMTLYRQLEGRGYSTTIWPARYPRDE

KDWKSYGDRLAPMLQAELESDPEGYYWLPTDEVRFDDEDLKERELSYGKAGFALQFML

NPNLGDAEKYPLKLRDLIVADLDPESSPMVYQWLPNLQNKRDDVPNVGLMGDTYHTYQ

TVGSAFSSYTQKILVIDPSGRGKDETGYAVLYQLNGYIFVMEAGGMRGGYEDSTLEAL

AKIGRKWKVNEYVIEGNFGDGMYLELFKPVAARIHPAAVTEVKSKGQKELRICDVLEP

IMGSHRLIVNSSTIVSDYQTAADKDGVRNPIYSLFYQMTRISRERGALAHDDRLDALA

IGVQFFVESMAKDAVKGQREVTEEWLEEQMEDPKKGFKSIETDYWANGVRVQFNTDDD
LGLGSYVQFH"

```
     CDS             complement(39337..39483)
                     /codon_start=1
                     /transl_table=11
                     /product="restriction modification system DNA
specificity
                     subunit, partial"
```

/translation="MVYFKLWRDYDYYHPPLSVRTLGKNSGVCLLRSCIKCAYAYSLS
LYYK"

```
     CDS             complement(39417..39506)
                     /codon_start=1
                     /transl_table=11
                     /product="ATPase 8"
                     /translation="MLHTSCQRWSTLSYGGIMIIITLLYRCEP"
     CDS             39505..39663
                     /codon_start=1
                     /transl_table=11
                     /product="gp19.5"
```

/translation="MGKTKAVLKALATNRATYRFLAAVLLAAGVTAGSQWVGWVETLV
CTLVSECH"

```
     CDS             complement(39577..39669)
                     /codon_start=1
                     /transl_table=11
                     /product="hypothetical protein"
                     /translation="MALMTLRHQSTYESLDPPDPLTSSSNASSK"
     CDS             39819..39905
                     /codon_start=1
                     /transl_table=11
                     /product="50S ribosomal protein L31, partial"
                     /translation="MGLLKRDDEFWTKSSRVHLTVQEPQVSP"
     CDS             39841..39954
                     /codon_start=1
```

/transl_table=11
/product="AC4 protein"
/translation="MSFGPKVRESISQFKNLKSPHRVTLSPTQGPTHRPSV"
BASE COUNT 9783 a 10037 c 11271 g 8969 t
ORIGIN
1 tctcacagtt caagaacctc aagtctcccc atagggtcac tctcagtccg actcaaggcc
61 ctacccatag accttcggtc taaacctcaa gtcattgacc cgaagtcgcc ccgaagtctg
121 gactgatagt gcctatatct tgtggttggg acgattggtc gggactatat gtagtgcctc
181 tgagtctcta tctgtatcga cctaatgtca atcacctaca gtcaaccagc ctacagtctc
241 actacctaca gtcatctacc atcggtaaag ccatcaccta cagccaatag acctacagtc
301 accagacact atgtcgacct tcagtttatc agagtcctaa ggtcttagac tcataggtta
361 tcacatccgg tccgtttagc agtcagagac catagctgac tagggttatc gttgttggtg
421 tctgccaacc ggactcttat tatccatagt acagacttaa agtcaatcat ctgtagtcgt
481 ccttatatag tcactgtact actgtataaa ttaccagtgt tgacagtggg tctaactttt
541 ggtttaatag cttccgtcaa cacgacacgg caaacaccgg atagtagaga caccgggtca
601 cacggttaag tagacagcct gataagtcat acgaacaaca ggcagtgaca aatagcttga
661 caagatgtag cgacttaacg tagtatgcac cacatcgaca cggcaccaac ggtcgataag
721 cagtaccaga ggcaccgcct cgccgctctt taacaatctg gataaactct taatgtgcgc
781 cgatagtaag cgattaacta cagggccttg agtctaccgc gagacgggct tgacttgagc
841 gactcagggt cctgactgat agtcactaac ttaaaggata cacatcatga tttacactaa
901 cgaaccagcc aacgtcttct atgtgctcgt gtctgccgct cgtgctaacc tgtctgacac
961 ggtgaacatg ggccgtcagt taggccttaa ggcgctgctg caagccggac cagaatacta
1021 cggcgtgctc gaatgcaccg ggcttactgg gtgctaccgc gaggaagggc aggaagtcgc
1081 cactgaagag aggacctacc gtgtgcgctg cgagtcaaag gctcaagcta tcaacgttgc
1141 acgactggcc tgcaatgact ttgagcaaga ctgcgtgatg gtctacaagt ctcagactca
1201 cacggctgga ctgatgttct gcaagggctg ggacggctac aagaccacaa gtctgaacgg
1261 gtcattccag caagtcgatg aaccaaaggg cgaatgcttc acgaaagacg ctgaaggtcg
1321 cttctgggag gtggtgtaat gattgcctat aaaatcttcc gaatccgcaa ggatggaacg
1381 ctgggagcac tattcatcaa tcgtaaggcg gtcattgagg taggagaatg gtacgaggca

1441 gagagccatc cgaccaaagg gtttgcctat cgtccgggct ggcactgcac ctttgagcag
1501 aacgcgccac atcttaagct ggacctgaag tcaggtgagc gtagggcttg gtttaaggta
1561 gaggttgaga acataacgac ctacgacagg ccagaatctc agggtggtgc atgggtatta
1621 gcacagcgtc tgcgggtatt agcacaggtc tcagagtgat aaactacagg tcatccacat
1681 gggtggcctg actgattgtc acttaaccat aaggaaacaa caagatgaac gcacacgata
1741 aagtaatcat gaataacctg atgtctcagc ttcacgctat aatctgtaag gccggttata
1801 aggtggagtc gttggggtcg ggcatcgctg cgcaggaccg ctggggagcc gttgaggtcc
1861 tagcccgtga gcatggctac aagctgttag gtctggggca cttcgccgct gcctttgagc
1921 atgaggacct gccggggtat gccatcaagg taggctttaa gaaggacgac tcaggcgctg
1981 cgtatgctgc cttctgccga gagaatgagg gcatggctgg actaccagtc atccatctgg
2041 tcaagcgctt cagcagggct tacatggtcg ctatggacaa gtaccgctcg cttgaggaca
2101 tcggtgggaa ctactgccgg gagggcaaga cctacgagca gcgagtctta aacgtgtcgt
2161 ggcgtgtagt caacgcgata atagaccagt gtgaacagcc atccgaagca ctgagctggt
2221 ggcttgacca agacgctcgc atagagtttg cacacgtcga gactcagtac atcaaagacc
2281 ttgcgcagac tgctaagaag attaacagct tcttctacgg gctggcatcg ttcgacactc
2341 atcgcgctaa cgtgatggtt gacaataacg ggcgcttgat aatcacggac ccggtgtctt
2401 ggactgcaag cgaccatcaa gatgagctgc aaggcaggct gaacgcaata gctgagtgat
2461 aggcgagaga ccgctacatc tagtggtctc aaagattatc tctcacaaca tataggcagg
2521 tgtacatggg ctggatactt ctggtcatcg gctacgggtt aatactcgca gtcatcacga
2581 aagacatcat caaggcacgt aaagtctacc gcttccagta tgtgtctctg ggtcgctgga
2641 ctgtaagaca accaaacgga cgctttatgc gtaacctcgc aaacgtctgg gacatcgcaa
2701 ccttagggag caaactgtaa tgtgtaagac ctgcgctaag tgtggcgaga ccaagagtct
2761 tgaccagttc ggcagcaata agcgcaacaa ggatggtctg gactatcgct gcaagacttg
2821 cagaaagcag gacgcccaag acaggaggca gaaggcacga gaggccggga cctacaagac
2881 gtcaaaggct gaagagaacc gaaagctgcg ctacggtctg gacctagagg agtacgagag
2941 cctcctacag cagcagggtg gagtctgcgc aatctgcaag ggtcagtgtc ctaccgggtg
3001 gagactggcg gtggaccact cacacgagac cgggaaggtg cgcggactgc tctgcctaca

3061 atgtaataca gcgctgggcc agttcaaaga ctcaccggaa ctactggcaa acgcaatcaa
3121 atatcttcag gagaatcaat aatgtacacg cacaagacct cacgtcacac cgttcgtcaa
3181 cagcaggagc gcgaccgggt actgcaagca agactccggg ccgagggcaa taaggccgag
3241 ctgatgtgcc tggcctacgg tggacgacca cgcactggcg atggtaaact tctggtctca
3301 ggctggaggg cgatagacca gcggtcatac atcaagcaca cgaccaaagg tgacttcagt
3361 catctccacg ctaacccact gatatgcaag tagtttggac taaactatca ctaaaggact
3421 caaggtcgaa agactctaag tcaggactga agtgaaagac tttagtgata agacaacagt
3481 tataaggact ttaagtatga gcgtcatctc aattgacaag cacgacttct ctgatgtgtc
3541 gaatgcaatc gaaccattca acgtgctggc tgaccactac ggtcaggacc tagcagtaaa
3601 acagcttcag cttgagcatg aggcgtacac tgaaggcgag cgacgtttca tcaagaacct
3661 tgagcgccaa gctgagcgcg gggagctggc agacaatcag gtcgccaagc cactgatgca
3721 aactctggtc ccgaccatcg ccaaggctgt tcgtgagtgg cacgaaggcc cggacggtaa
3781 gtcctcgacc tctcgcccaa gcgtagcgtt taccatgctg agcactgacg agaaggctgt
3841 caaagaccgc tctctgcgaa tctctgctga gtctgctgcg gtcatcatac tgaaagtcat
3901 cctcagcaag ctggtcaagc ctgagggaat cccgataacg ccgatggcct ccgcgattgg
3961 tcgcacactt gaagatgaaa tccgcttcgg tcgcatccgt gagcaggagc aggagcactt
4021 caagaagacc atagctgaga atctgaagaa acgtgcagga gcaagctaca agaaagccta
4081 catgcaagcg gtcgaagcct ccatgctgga gcaagggcaa ctggctgacg cttgggggac
4141 ttggagtccg accgaggcgg tacacgtagg catcaagatg ctggagctgg tcatccagtc
4201 ctcgcaactg gtcgaactga agcgctacgg tgcgggtaac gctgcggcag acgtcgagat
4261 ggtccacctg tcagacttct gggtcaagaa gatgtcgcaa cgtggcttct cactggctgg
4321 catcgctccg gtctaccaac cttgcgtcgt tccacctaag ccgtggaccg gggtcgtggg
4381 tggtgggtac tgggctaagg gccgaagacc tctcccgctg attcgcttag ggtccaaggc
4441 tgcggtccag cgctacgaag acgtgtacat gcctgaagtc tacgaggcag tgaatatcat
4501 ccagcagaca ccttggaagg tgaacaagaa ggtgctggac gtggtgaaca tggtcgagaa
4561 gctgaacaac acgcctatcg ctgacatccc tcagatggaa ccgatgaagc ctgaggacta
4621 tgcaggtgag accgaggaag aactcaaagc gtggaagaaa gccgctgctg gtatctatcg

4681 acgcgagaag gcccgacagt cccgccgttt gtccctgagc ttcatcgtcg gacaggcgaa
4741 caagttctct cagttcaagg ccatctggtt cccatacaac atggactggc gcggtcgagt
4801 ctatgcggtc ccgatgttca accctcaggg taacgacatg cagaagggcc tgctgactct
4861 ggcagtaggc aagccaatcg gtgcggacgg cttcaagtgg ctgaaggtcc acggtgcaaa
4921 ctgcgctggt gtcgataaag tcaccttcga ggagcgcatc aagtgggtcg aagacaacca
4981 cgacaacatc ctcgcggctg ctaagagtcc gatggatagc attgactggt ggggtcagtt
5041 agactctccg ttctgcttcc tcgcgttctg cttcgagtat gctggagtca tgcaccacgg
5101 gctgagctac tcctgctcac tgcctatcgc gttcgatggg tcctgctctg ggattcagca
5161 cttcagcgcg atgcttcgtg accacgttgg tggacatgcg gtcaacctga cgccaagcgg
5221 gaaggtccaa gacatctacc gcattgtgtc agaccgggtg gaagaacagc ttaaggagct
5281 gctggtcaac ggaagtgaca acgaggtgaa gaccttcgag gacaagaaga caggcgagat
5341 tactgaacgt ctggtccttg gaactcgcga gctggcccgt cagtggctga cctacgggat
5401 gtcacgctcg gtcactaaac gctcggtcat gactctggcc tatgggtcga aggaatacgg
5461 gttcgcggac caagtgtttg aggacaccgt gatgccagcg attgacagcg gcaagggcgc
5521 gatgttcacg gacccaagcc aagcgtcacg cttcatggct aagatgattt gggatgcggt
5581 aagcgtgacc gtagttgctg cggtagacgc gatgaagtgg ctgcaaggcg ctgctaaact
5641 gctggctgct gaggtcaagg acaagaagac aaaagaggtc cttaagcctt gcctcccggt
5701 tcactgggta acgcctgatg gcttcccggt ctggcaggaa taccgaaaga aggacaccac
5761 ccgtctgaac ctcctgttcc ttgggtcgtt caacctgcaa ccgacagtca acaaagggtc
5821 taagaaagag ctggacaagc acaagcagga gtctggtatc agccctaact ttgtacactc
5881 acaggacggc agtcacctga ggaagactgt ggtccacacc caccgcaagt atggcgtgat
5941 gtccttcgca gtgattcacg atagcttcgg gaccatcccg gctgacgctg agttcctgtt
6001 caagggtgtc cgtgagacga tggttgagac ctaccgcgac aacgatgtgc tgcaagactt
6061 ctacgagcag ttcgcagacc agcttcatga gagccagcgc gacaagttgc ccgagctgcc
6121 gaagcgcggt aaactgaaca tcgaagacat tctgttatct gactttgcat tcgcctaagg
6181 aggcatctac atgaaattcg cacacaagca aactggcgtt aaaggcggta ctcaaatcgt
6241 gaccgtgacc gagcacaatg gcaaaggtct ggtgaagacc acggtcatcc caaccgagat

6301 gtcaaagcag cttaacgtcc cattcaagtg gctggtgaag ctggttcaag agcgacacga
6361 gtcagaactc cgcgaggtgg taacgaaatg acagacctta agtggctggc cctatggctg
6421 gccttcttag cagtgtacat cttaatccaa cgcagaagag gctaaacacc atgttcaacc
6481 gtaaactgaa ggcacgtatc gcagcacttg aagcaaccat acagcgtcag gaccacaacc
6541 ttgagctggc taatcgagag caagcacggc tgagggccaa actacaaaaa gctgagacca
6601 agcgtgaagt cgagcccttta cgtgtgaagc gcatggtcgg cctgaagttc aaggcgctgt
6661 tcaacggtat ctaccagccg actgagttca agaccgggcc gggaccatgt ggaaagacag
6721 taacgcactt caccacgagc cgagacgagc ggacgctgac catccaccag caccatacgg
6781 acgagtccag caagagcttt gagtatcggc tgagcgacat cgacggtcgc atccagtatg
6841 actacgagct ggtgcaggtt tacggtgagg aggcagagcg ggaacgtatg gtccaagccc
6901 tccgcaacag accgcgcttc atagcctaaa ctatcactat aggattagac tcaaggtcat
6961 gactcaaagt cgcggccttc atgattaacc ctgtagcaaa gctactacct aactcatcca
7021 atcaggagct acaccatgta tcagaccatt atcaacttcg agcgcatccg tgaacgtcag
7081 cagactgaag gctacattcc gaagggccgt aagctgaaca agacaaagcg tggcggtggc
7141 gtgaagggtg ctttccgtaa cgctgaaggt aaggactctc tggtcaacca agagaagtat
7201 ttcgtaggag cgtaaccatg agcagacgtt ggttattcga tgcgtccacg agccagtggt
7261 cctcgttagg ccggacacac cgcgagatgg gtgaatcagg acttgaggtc cgaatggatg
7321 agctgccttg ctcccggaca actctggtca gaatcactga agcagatggg aagcctctgg
7381 tatcccgcga gtttaccgaa catgaaatct ttagctgtga gcaatggtgc ttgcaggtgc
7441 tgaacgacta taggagctgg aaataatgac tactacactc aagaccaacc cgcatcgcgc
7501 tgtagattac tctgagtccg gcgttaagaa agcactggac gcagccgggt cgctggaagc
7561 tgaagtgaag tacgacggtg tgcgtctcaa cattccggtc cttcgtgaag gtgagaccta
7621 ctggctgagc cgtgagtcga aacctctgcc agccttggag tggatgaact ctgagttagg
7681 caatgcttgg acccaagctg actggcgctg gttcctgcgt caagctggct acgaaggtgt
7741 gggcctgatg attgacggcg aggtcatggt caaaggtgtg gacttcaaca catcctcagg
7801 cctcatcaga acgaagtggc tgaagcctaa caacgaggcg ttcgctgaat gttatccggg
7861 ccgtggcaag aaggtcccgt tctgggtcgc ccggtcccga cttcaggttg tggtctacgg

7921 tgtcgtcgac atgacgacca tagccgaccc gaaagcagaa ggtcctatcc
atagcgtcac
7981 ccgcctgaag gccgaagcta tcgtccctct cctccagaaa tacttcccgg
aaatcgactg
8041 ggttctgtct gagtcacaca cggtctttga ccttgagtcg ctcaactccc
tgtacgaaga
8101 gaagcgtctg gaaggacacg agggtctggt aatcaaggac ccgttgggta
actacaagcg
8161 tggcaagaag tcaggcatgt ggaagatgaa gcctgaggac accatcgacg
ggactgtatg
8221 tggcctcgtg tgggggactc agggtaaggc caacgaaggc aaggtcatcg
gctttgaggt
8281 gctgctggaa gatggcatgg tggtcaacgc ctgtggtctg accgaggagc
agaaggatga
8341 gttcaccgcc aaggttaaag aggacactct cgccgctgtg ggcatatttg
ctccgtatgg
8401 tattggcggt aacgaaccct gcttcgataa cccatacgaa ggctggcagg
ttgaagtcct
8461 cttcatggag cgcttcccgg acggctccct gcgccaccca agtttcaacc
agtggcgtgg
8521 cacggaagac aatccgacca ttaagagcta actgaacgtc aacccagtgg
tcttcgcgga
8581 ctgctgggtt tctttgtgtc tggactccgg gccgctggac ttaactatca
ctgtaggacc
8641 acaccaggtc caagacttta agaggagaaa cctatgtcta agaacttaat
gttcaaccgt
8701 ttcagtcaga ccttccacct gtcacacaat ccgttcgctt gcattaagcg
taacgagaag
8761 gtgggcttct tcgggaaggc cgttaagctg gcacctacgg tctacgctct
gattgttccg
8821 agcaaagtag aagaggctcg ccagaagaaa gagaccagtg taccagtggt
ctacaccaag
8881 tggcctcgcg ttcgtctggt gctggagttc atcaaggagg agctctaatg
gctcattatt
8941 gtgtggactg tcagaacgac cataccgaat gcacctgcca cctgaagtat
cagacaccag
9001 cgccaagctg ttcgccaacg tacactggtg ggtctgccga ttactaccaa
gtgcctatca
9061 ccaagactac gactcccggt cgaccggact acatcgctga atgcaacgac
attatcgagg
9121 ctctcggtat gaactttgca gaaggcaatg cgttcaaggc catctggcgt
cgggctgctc
9181 aacggacctt aggtctgcgt aaggctggtg ctaaggacga tggactgtat
gatgctgaga
9241 aggtcgagtt cttcggcgct cgtctggtag cacaaagcaa ggcggcaggt
catgactcca
9301 agtgaatggt gcgagatgat gtttgagaag acgggtaacg tcgactatct
cgaaatgtat
9361 aacctctgga aagggagagg actatgacca tctataaggg catcttcggg
tctctgtacg
9421 cagtgaacca acacggagtc gtctgcgtta agccggacgg tggcgagtgg
tgcgcatcac
9481 attacagtgt cgagggattc caagacgcta ttgataaagg ctatgtgacg
gaggtggtcg

9541 atgaccaagg agtcaactga gaagaagtat atcgtggaac ttgaagggcg cgttcagtcc
9601 tttgacgtcc cggtctacgc taagtctctg gacgaggcga ccctgaaggc tcaggaatat
9661 gaagatgctg gtttcgtggt aggccgcatc cgacctgagc tgcacgacta aactatcact
9721 ttaggaacac cttaacttta actaggagat ttaccgatgg ctaaagagca actgaaaact
9781 ttcaccactc cggtcgctgg tctggtcgag ccgtatagct ggctgaacaa gtcagacacc
9841 aagttcaacg agcgtggtga gcacaaagtc aacctgacgt tcgacctgag cgacccgaaa
9901 gttcgcaaga tgattgacgt cttgcagaag attcacgacg atgcgtatgc gaaagcactc
9961 gcagaccacg agaagaaccc ccctcaggtt cagcgtggca agaagcctat cgaaccgcgt
10021 gaaggcgata tgccgtggat tgagaacggt gatggtactg ttaccctgaa gttcaaatgc
10081 tttgcgtctt acttgaaaga cggcaagtcc gagcctatcg tattacggtt ctacgacacc
10141 gatgctaaac tcatccgtga cgtcccgaat attggcaccg ggtctaagct gaaagtcaag
10201 ttcaaagtcc tgccgttcaa gtggaacgct gcgactggtg caagcgttaa gctgcaactc
10261 gaatccgttc tgctggtcga actcaaagag tggtccggcg atggtactgg tggtgatggt
10321 ggctggggtg acgatgaaga catcggctcc ggtggctaca aagcgtctac tgacggtgac
10381 ttcgggtctg atgacttcgg tgaagatgcc gatggtggcg atgactctgc gtccggtggc
10441 gactacgact tctaatcgtg gctgcatgga caccaaaacg ggggcactct gtgggtgcct
10501 accgctctgg acttgaggcc aagaaccagc agtggctgga acagaacggc gtcaaagcgg
10561 agtacgaaag ccattatatc aactatgtga ttccggcttc cgaccacaag tatacaccag
10621 acttcatcct tccgaatggc atcatcgtcg agaccaaagg tatcttcgac agtgaggacc
10681 gcaagaagca tatgctggtg cgcgagcagc accctgagct ggacatccgg ttcgtgttct
10741 cgtcctcccg ctccaagctg tacaaagggt cgccgaccac gtatggcgca tggtgcgaaa
10801 agaacggctt taagtttgcc gacaagttta tcccggttga gtggctgaaa gaggcgactg
10861 tacgtctgcc ttccggtata ctcatcccga agaaaggagc taagaaatga ctaagcattt
10921 aacgtgtgtt gatttaccat ttggtgctca gtacaaattc caccctgacg tcattgaggc
10981 gcacccagga ctgagcactg acacgtatga gcgtcgggat aatcactcgg ttgttgtaac
11041 gggtccattc aaggggaaca ctgtacaatg gtacggacat gagaagactc gcattgtcga
11101 agttggaaaa cctcaagtcc tgccagcctc aagcgtaccg cctgtgacag tgaaacgtga

11161 agtcctgacc atcgacaaga ttggtgtggg tcagacgttc atcgttcacg
gtaagcctga
11221 cgaagtgtac gtgaagatta gcaactcgca cgtcttcaac cataagcgtc
tccagatgca
11281 cacgactgta gcagagcgct tccagtcaca cctgaatctg gtcgttgttg
agctggtggt
11341 gtacaatggc aagtaaggta cagttcaaac cacgtacagt cactgacgca
atcttcgtcc
11401 actgtagtgc tacacaaccg tctcaggaca tcggggtaga cactatccgc
atgtggcaca
11461 agcagcaggg ctggcttgac gtaggctatc acttcgtcat caaacgcgat
ggcaccgtgg
11521 agtctggacg tccggtcgat gtcgtagggt cacacgttaa ggattggaac
tcccggtccg
11581 taggcgtctg ccttgtaggt ggaatcgacg ctaagggcaa gtttgaagct
aacttcactc
11641 ctgctcagat gagcgcccta cgcaacaagc tggctgaact gaaggtcctg
taccctcagg
11701 cagacatcaa ggcccatcac gatgtggcac caaaggcttg cccaagtttc
gacttgcagc
11761 gctggctgaa cactaacgaa atggtcactt ccgaccgagg ctaacaagga
gtaataacat
11821 gattaagttt atcgaatttc tgggtcgtct ggttgtgcgt ggttattctc
gtgctgcgag
11881 tgtggaacga aaagtcgcat ctgctgcggc taaaggtgcg gaagcggctg
ctgcggaagc
11941 ggatagtctg cgcatcaagt ctctggacgc tggcctccgt gcgcgtggtc
tggacaagaa
12001 ggccgagcaa ctgaaaggct tcttcagcta aactatcacc ttagggatga
gaccatgagt
12061 cttgtccctt tgttcgcact attgattaag gagtgaccaa tgtcatatga
agaccaagag
12121 caggacgata gcgtcttcct ctaccacacc gagtgtccag actgcgggtc
ttcggatgcc
12181 aatggtgttt actcagacgg gcatatgtac tgctttgcgt gtgacccgtc
agtcgcttgg
12241 aagaagggcg atatggaatt gaccgaaggt tacacacctt caggaggcaa
gaagcaagtg
12301 agcaacctgt taacgtgggg tgagaactca ggacgttatg tcccactccc
tgctcgtgga
12361 ctatcggctg acatctgcaa gaagtacagc tactgggtag gcatgatgca
gggtaagatg
12421 gtccagattg cggactacta cgacaggtcc gggaccaagg taggacagaa
ggtccgagac
12481 gctgacaaga acttcacggc tatcggtagc gtcaagaatg acttactgtt
cggctctcag
12541 ctctggtcag gcggaaagaa gattgtcatc accgaaggcg agattgactg
tctgtcagtc
12601 gctcaggtgc aggaaggcaa gtacccggtt gtctctcttc cgctaggcgc
gaagtctgcg
12661 aagaaaacac tggcagctaa ccttgagtac ctcgaccagt tcgaagagat
tatcctgatg
12721 ttcgacatgg acgagccggg gcgagaggct gtagagcaag gtgctccggt
cctccctgct

12781 ggtaaagtca aggtcgcatt catcaatggg tataaagacg ccaacgctgc
actttcaggcc
12841 aaggacttca gggcgattca ggatgcaatc tggaacgctg tacctttcgt
cccggctggg
12901 gtggttagcg ccaagtctct gaaggacagg acccgcgagg caatgctgaa
ggctgagact
12961 gaaggtctcc tcttctcgtc ttgcaaaaca cttaacgcga tgaccctcgg
tgcgcgagct
13021 ggtgagctta tcatggtgac ttcagggtca ggcatgggta agtccacctt
cgtccgtcag
13081 ctcctcttag agtggggcag aatcgggaag cgtgtgggta tggcgatgct
ggaggaagca
13141 gtcgaagaga ctgttcagga ccttatgggt ctggacaata acgtcaggct
gcgtcagtgt
13201 gccgacctga aggaagcaat cctgaaggat ggacggttcg acgaatggta
tgacaagctg
13261 ttcaacgacg ataagttcca cctatacgat tcattcgctg agtcggaaga
agacacgctg
13321 ttcgctaagc tggcctacat ggttgacggt ctggactgcg atgttattct
gctggaccac
13381 atctcaatcg tggtgtctgg catggaagat aactcagatg agcgtaagac
catcgaccgc
13441 atcatgaccc gtctcaagaa gtttgcgaag acgaagggtg tggtcgtcgt
ggtcatctgc
13501 cacctgaaga acccggagaa aggtaagtca catgaagaag gtcgtccagt
tagtatcact
13561 gacctgcgtg ggtctggtgc tttacgtcag ctatctgata ccatcattgc
cttggagaga
13621 aatcagcaag gtgatactcc taacgttgtt cagctccgtt tgcttaagtg
tcgctttact
13681 ggcgatacag gcattgcggg acatcttgaa tacaacaagc tcactggctg
gctcgaaccg
13741 attagcgcac ctagcggagg cggagaggaa gatagcagct cgtgggaaaa
ccaagacttc
13801 taaatgtcaa tgccctgagt gcgactggaa gtcaccatat ggaggacacc
attagtgttt
13861 aagaaactta aagcccgata ccacacgttc atgtacaagt ggtggaacga
ggaggccaac
13921 tgtctgtccc gaatccttgg agaccagcgg tttgactcta aggcgtggaa
gcgagcgaac
13981 cagaagttca tgtatcactt cctgcgtatc gactactaag ctatcactgt
aggatagact
14041 caaggtcatt cacttcgagt ggcctttatg attagactat aggagacatt
atgattgact
14101 tgcagaacat ctggggttcg gacatcgaga ccaacggtct tcttgatgcc
gtgtctcagt
14161 tccactgtgg ggtcctgatt aacgccgagt cgaatgagac cctgaagtat
ggggtagctc
14221 cgatggtagg tatcgtcggt ggcttcaaag agtatgtgca gaaggtcgaa
gagattgcag
14281 ccacaccgca tgggcttctg gtcttccaca acgggattaa ctatgacgtc
ccggctattg
14341 acattctgaa gcgtaagtat ttcggtaaac gtttcaactt cccgaaacag
aagatgattg

14401 acacgctggt catgggccga ctgatgtacc cgaacattaa attctcggac atcggggctg
14461 tgaaagctgg tcgtctccca cctaagatga tgggccgaca gtcgcttgaa gcgtggggct
14521 atcgtctcgg tgagatgaag ggtgagtaca agaccgatta cattgccaag tgcaggacag
14581 aaggcatcga atataaggct ggcgacgagt ggctattccc gagtcaagag atgctggact
14641 ataacgtcca agacgttgtg gttactctgg ccctgttcaa gaagttcctg accgataagt
14701 tctacttcgg gacacctgaa gctggacttg aagcagtgta tgcgttacgt ctggaacatg
14761 acgctgcgtg gacctgcgcg aagatggagc gtaacggcta cccgatgaac tcggagatag
14821 tcgaaggctt gtatcgcgag ctgtctatca aacgtgctga gcttttggat aagctgcggt
14881 cgactttcgg tagctggtat tcaccgaagg gtggcaagga gttcttcagg caccctcgct
14941 ctggtgtgga cctgccgaag tacccacgag tcatctaccc taaggtcggg tccatattca
15001 agaagccgaa gaacaaagca cagcgcttag gtctggagcc ctgcgaacga gactcccgcg
15061 atactatgga gggcgcaccg ttcacaccga ttagcttcgt tgagtttaat ccgggaagtg
15121 gcgaccactt ggctaaggtt cttatggacc gaggctggga gccgactgag ttcacggaga
15181 ccgggaagcc aaagtgtgat gacgaggtgc tggagaacgt acatctggca gaccctgagg
15241 ctcaggcttg cgtagagctg gtccgtgagt atctggtggt ccagaagcgt atcggtcagg
15301 ccgctgaagg taagaacgca tggcttcgac tggtgggccc tgacggaagg atgcacgggt
15361 cgattaaccc ttgtggtgct gtaactggac gcgcgactca tagttcaccg aacatggctc
15421 aagtcccggc taacggtgct ccttggggtg aaatctgccg tagtgctttc ggtgctcagt
15481 ggaaccagaa tgctggtaag ccagaccctt ggattcaagt tggggtcgat gcctcaggtc
15541 ttgaactccg ctgtctgggg aaccgagcgt ctccgttcga tggtggcgaa tatgcgaaga
15601 ccgtggtcga aggtgacatc cactgggcca atgcggtcaa cgccgggtta gctcctaacg
15661 taccacgcga taagtccagc cacgaccacg atgctttccg taacaacgct aagacgttca
15721 tctatgcgtt cctgtatggt gcagggggccg cgaagattgg actgattgtg ggcggtggta
15781 agaaggaagg cgcggccctg atgaagaaat tcattgaggg tacaccagct atcaaggacc
15841 tgcgagaggc ggtgcaaaac acgctcatct ccgagtccaa gtgggtagac ggtgagaaca
15901 tcgtgaagtg gaaacgccgc tggcttaagg gtctggacgg tcgacgcatt cacatacggt
15961 cgccacactc agcactcaac gccctacttc agggtgacgg tgcggtggtc tgtaagcact

16021 ggattgttga gaccgagcgt atgctcgaag aggccgggta tgtccacgga
tgggaagggg
16081 actttgcgta catggcatgg gtacacgacg aattgcagat tgcggctcgg
accgtggaga
16141 ttgcagaaga tatacgtagg attgctcaac ttgctatgcg taaggtgggt
gagttctata
16201 actttaaatg cgtccttgat accgaaggta agattggacc aacgtggaag
gagtgtcact
16261 agtggctatt actaaacgta ttcgtgtgag ttttgacctt aagctggttg
tgtgttccaa
16321 gggcgaggaa atctcctgcc gcaacttagc gaagctgtcc aagatgttca
ctgagggtga
16381 gaagctggat ggcatccagt tggccctgat taaggaagca gcagagtctg
gaccggaagc
16441 agcccttgag ctggccttta agaagtccat caaggagtca gtcgtcgagg
cgttagacga
16501 tgagcccttc actgcttcaa acttccgctt cgaggttaaa cgatgagcga
atacctgcgg
16561 gtccttgcgg ccctcaagtc ctgcccgaag acctttcagt ccaactatgt
gcgcaacaac
16621 gctgcactag tggctgaggc tgcgagtcgt ggacatctga gctgcctgtc
tatggacggg
16681 cgtaacaacg gtgcgtggga gattaccgct gctggcacca agttcctgaa
ccaacatgga
16741 ggctgtctat gagtcaacaa cctttggact ttctgggtca acctatcgaa
gttggcgaca
16801 agttcgtcta cgctgaggct ggtggacgtg gcggcacaat ggcgctgcac
tcaggtgtcg
16861 ttacccgcat gactgagaag caggtgctgg tggagaagac ccgctggggc
cagacttggc
16921 gtccgttcaa ctgcatcgtc attgtcgaga agtgtggacc agacgaagaa
cctcttctgg
16981 gtgcaacctt agctatggct agaggaggtc tccgggatga gtgacaagaa
gattgctctg
17041 gtgctggatg gtgactttct tgtcttctcc tctatggctg cggctgaaga
cgagactgac
17101 tggggcgacg acatctggac gctaatctgc gaccacgaga aagctaagcg
catcctgatg
17161 aacactatca gtgaaatcgt taagaagcgt aaggcttgga aggacgctaa
gattgtgatg
17221 tgcttcaccg atgatgtgaa ctggcgcaag tcaatcctgc cgacctataa
ggccaaccgt
17281 aagggttccc gtaagcctgt aggctacaag aagtttgtgg ctgacatcat
ggcggaccct
17341 cagttcaaca gcttcctgcg ttccacgctg gagggtgatg attgtatggg
tatcatcggg
17401 acacgacctc agattgtagg ttgcgaccat gcggtgctgg tgtcctgcga
taaggacttc
17461 aagaccatcc cgaactgtga gttctactgg ctgaccactg gtgaaatcct
gagtcacacg
17521 actgctgagg cagactactg gcatatggag cagaccatca agggtgacat
gacggatggc
17581 tatggtggta ttccgggcta cggagaggac acgacccgtg cgttccttga
cgaaccgtac

17641 tacttcgtgc aggagacccg cgagctgaag actggcaaga acaaaggcca
gctcaaggtc
17701 gagtggaaga aataccctaa gcgtgaagac caaacgttgt gggactgcat
ggtgactctg
17761 gctgctaaag ccgggatgac cgaggaggaa cttctggtcc aagctcaggt
cgctcgcatc
17821 tgccgagcct ccgactacga ccctaagtcc aaggaggtca tcttgtggac
accttacatc
17881 taatctactg ggccggactt ctggcccttt acgcgatgta cgcaatcagt
gggtctaacc
17941 gaccgaagca ctgagtctaa ccgatagtca taacctatcg ttctactggc
catctatagg
18001 tgaaacacta aactatcact atagggactt taggacctaa gttatgacta
taagatagac
18061 ttaagttaat actttaagag gagactttaa gatgggcatt aaggccattg
atgatgttgt
18121 taatcaactc gtagaggaga gacttaaggt ccctgcattc tccgagtctg
ccgtccagtt
18181 cctgcacatc ctgttcaacg cgagctatgc gtctaagctg ggtgtcatca
acgacctcaa
18241 ggctgctggc cacagtgaag gctatatcgc tgggttcatc ggcggtcttc
agtattgttc
18301 agacactctg gactctgcgg tcgctaagcg tcaaagcctg aaggacaaca
ttcagttcga
18361 ctaaaggagg gactatgtgt ttcagtccga agattagcac tccgaagcca
tccaccgctg
18421 cacctgagcc agcccctctg agtgaggaag tagctagtgt cgacatcggg
gctgaggaag
18481 gtgcggacac caacgagacc aagggtatca aagacctgaa agtcaagaag
gagtcaacag
18541 ccactaaatc gtccgtgggt cgcgctatgc gtaacactgg cgtcaacatg
gggtaagact
18601 atgctccact accgaaactc aagcgatggc cgccgcatgg ctgctgaacg
cctctgggaa
18661 gacggacagt ctgacttcgc ctcattctct gagttcaaac aacatacctt
ccgtatggct
18721 gatgagcttg agggtgagga gtacacaatc tacgatgacg agtgtaagcc
tgtggcctac
18781 ctctacatgc tggcgtcatc gtcttggcac cgaaagactc ccggtcttga
cctttccatt
18841 ctcgctatcc gtagtgactc gcagtcctcc cgcaaggttc ttgagactgt
cagacacatt
18901 attgacggag agtgcagacg atggggtctg aactggtggt cccgtgtcaa
gcacaatggg
18961 ccagtagaca tcgttacaac caaggagatt aaccgtgggt aaatccatta
gtaaagcatt
19021 caagaaggta gtgggcggtg cgttgaatac tgtaggtctt ggaccttctg
ctgcttcagt
19081 tcctaaggct gctgaccaac aggtagccgc tgctccggtc gaagtaccga
aagatactgt
19141 gactgacgtg gaaaccgatg tgaccgcaga gggctcaaag aaaggtaagc
gtgtaggtaa
19201 gcgcagcctg agcgtctctc gcacttccgg tggcggcatc tcaatctaag
cgggggtgat

19261 taatggctga acgtcaaggc ttcgctgctg aaggagccaa agcggtttat
gaccgattaa
19321 agaacggtcg ccagccgtat gagacacgag ctcagaactg tgcggctgtc
accatcccgt
19381 ccctgttccc gaaagagtcc gacaactcct caacagagta cacgactccg
tggcaagctg
19441 taggcgctcg ctgtctgaat aacctagcgg ctaagctaat gcttgccctg
ttccctcagg
19501 caccgtggat gagactcacc gtctctgagt atgaggccaa aaccttgagt
caagacactg
19561 aggctgctgc ccgtgttgac gaggggctgg ctatggtcga gcgtgtgctg
atggcctata
19621 tggagaccaa tagtttccgt gtgccactgt tcgaggccct gaagcaactt
atcgtctcag
19681 gtaactgtct gctctacatc ccgcctccag aacagggtca gtatagccca
atgcgaatgt
19741 accgtctggt ctcctatgtg gtccagcgcg atgcgttcgg taacgtgcta
cagattgtca
19801 ccctcgacag gattgcgttt agtgctctgc cggaagacgt tcagtctcag
ctcaacgctg
19861 atgactatga gccggacacc gagttggacg tctacacgca catctaccgg
gaaggcgatg
19921 agtacctgcg ttatgaggaa gtggaaggcg tagaggttaa cgggaccgat
ggttcctatc
19981 cacttgatgc ctgcccgtac atcccggtac gcatggtcag actggatggt
gaagactatg
20041 gtcgttccta cgctgaagag tatcttgggg acctgaactc tctggagaca
attaccgagg
20101 ctatcaccaa gatggctaag gtcgccgcta aggtggtggg tctggtcaat
ccgaacggta
20161 tcacccagcc tcgtcgcctg aacaaggcag ctactggtga gtttgtggcc
ggacgcattg
20221 aggacatcaa cttcctgcaa ttgacgaaag gtcaggactt tacgattgcc
aagtcggtgg
20281 ctgacgctat cgagcaacgt ttaggctggg ccttcctgct taactctgct
gtccagcgta
20341 atgctgagcg agtgactgct gaagagattc gttatgttgc tggtgagctt
gaggcgactc
20401 taggcggtgt gtactccgtg cagtctcagg agatgcagct acctctggtc
cgtgtgctac
20461 tgaaccagct tcagtccgct ggcatgattc ctgaccttcc gaaagaagcg
gtagagccta
20521 cggtctccac tggtctggaa gctctgggtc gtggtcagga tttggagaag
ctgactcagg
20581 cggtgaacat gatgactgga cttcagcctc tggctcagga cccggacatc
aacttgccga
20641 cccttaagct gcgactcctg aacgctctgg gtatcgacac cgctggtctg
ctcctgacgc
20701 aagacgagaa gctgaaacgc atggctgagc agtcgtctca acaggcagtc
gtccaaggtg
20761 ctggtgctgc tggtgctaac atgggtgctg cggtcggtca gggtgctggt
gaggacatgg
20821 ctcaggctta actatcacta taggaacaac aggtcagact cttggcctgt
tacctattaa

20881 ctttaagaca ggaggactta atgtctcaat cagtttatgc cgagttcggc gttagcccta
20941 atgcaatcac tggctccgtt gaggacctga acgaacatca acagtctatg ctggagaagg
21001 acgtagctgt ccgtgatggc gatgatgcta ttaccttcaa gcaactggaa gccgagcagg
21061 aagaggcgac cgaagaagac gagaacgtcg aagagactga agaggaagaa gaggttgaag
21121 aaaccgaaga ctccgaaggt caagaccaag agttcatcga actgggtgac actccgaaag
21181 agctgaccga aagtgtctct gctctggacg agaacgaagc tgcattcgat gacatggtgt
21241 ctgctgctgt agaagctggc aaggtcactg ctgatgacat cgccactatc aaggctgaat
21301 acgctgcgaa cggtgagctg tccgaggcat cctacgctaa gctggctgaa gctggctaca
21361 ccaagcgttt cgtcgattcg ttcgtccgtg gtcaggaagc tctggctgag cagtatgctg
21421 ctggtgtgat tcgctacgct ggtggtgctg agcagttcaa ccgcatcctg tctcaccttg
21481 aggccaacga caagtccacc aaggaggccc ttgagtctgc catcatccgt aaggacttgg
21541 tgacagctaa ggctatcctg aatctggctg gtcgtaacct cggtaaggct cgtggtgtcc
21601 agcctcagcg taccatcacc actcagggta aaccagcggt ctctgcaccg aaggtcgaga
21661 ctgaaggctt cagctccaag gctgatatgg tcaaggccat gagtgacccg cgctatctgc
21721 gtgacgctaa gtacacgatg gatgtacgcg ctaaggttgc tgcgtctagc ctgtaagctg
21781 gactaaacta tcactatagg gagaccaaga gtagagactc aaggtttccc tgtaacttca
21841 gtccatacgg attgggcata acgaagtcac taaactttat ctttcattcg aataggagaa
21901 ttatcatatg gcaaacgtac cgggtcagaa aattggtcag aaccaaggta aaggcaatac
21961 caataccgac cagctcgcac tgttcctgaa ggtcttcgct ggtgaagtcc tgaccgcttt
22021 cactcgtcgt tccgttactg ctgacaagca tattgtccgc accattcaga acggtaagtc
22081 cgcacagttc ccggtcatgg gtcgcacctc tggtgtgtat ctggctccgg gtgagcgcct
22141 gagcgataag cgcaagggca tcaaacacac cgagaaggtt atcaccattg atggtctgct
22201 gaccgctgac gtgatgattt tcgacatcga agacgctatg aaccactatg acgtggctgg
22261 cgagtattcc aaccagctcg gtgaagctct ggccatcgct gctgacggtg cggtactggc
22321 tgagatggcg attctgtgta acctcccggc tgcatcggat gagaacatcg ctggtctggg
22381 caaagcgtcc gtactggaag ttggcacaaa agctgacttg aacactccgg ccaaacttgg
22441 tgaagcaatc atcggtcaat tgaccatcgc tcgtgctcgt ctgacttcca actatgttcc

22501 cgctggcgac cgttacttct acaccacgcc tgacaactac tctgcgattc
tggctgctct
22561 gatgcctaac gctgctaact acgctgcgct gattgaccct gagactggca
acatccgtaa
22621 cgtaatgggc ttcgttgtgg ttgaagttcc gcacctgact cagggtggcg
ctggtgagac
22681 ccgtggtgat gacggtatct ccattgcttc aggtcagaaa cacgcattcc
cagcgacttc
22741 cagcactacc gttaaggtcg ctctggacaa cgttgtgggc ctgttctctc
accgttctgc
22801 tgtgggtact gttaagctgc gtgacttggc gctggaacgt gaccgtgacg
tcgatgctca
22861 gggcgacctg attgtcggta agtacgcgat gggtcacggt ggtctgcgtc
ctgaagcagc
22921 aggcgcactg gttttcacag cggcgtcagc gggttaacga cctttaaggc
cctctctaca
22981 gagggtcctt ccttagagac ttacagttct acggtgggga ccagcttgaa
ggttgacttc
23041 cctgagcttt cagacgttac cgattggtcg ctgcttgagg tgactacgcc
tgatggtgtt
23101 agttactccc gtagaaccaa cagtctctac ttcaaggcca cctctggcgt
ggaaggtctg
23161 gtgattgttg ggtatgacgg ttcgcctgtg cgttccttca acgtatcttt
cacaaactaa
23221 ttgaaacccc ttgggtgcct tcgggtgctt gaggggtttt tcgttaagag
gaggactcat
23281 ggcagtctat atcccactaa atactaacga cgacttagac gctataaatg
acatgctggc
23341 ggctatcggt gaacctgcgg tccttcaact ggacgaaggg aacgctgacg
tctcaaacgc
23401 aatgcgcatt ctacagagag tcaaccgtca ggtacaagct aagggctgga
actttaacat
23461 caacgaggct gcggtcttga ctccagacat atctgacaac cagattcgct
ggctaccgtc
23521 ctaccttcgg gtaatgacta ccggagccac cagctattac agtaacatgg
gtggttacgt
23581 ttatgaccta agcactcagt caacaacctt cacagctgca atcacagtcg
agctggtaga
23641 gctgaaaccc tatggtgaga tgcctgttgt cttccgggac tatatcgtga
ccaaggcaag
23701 ccgagagttc aacgctaagt tcttcggtag ccctgaggct gagatgtatc
tccgtgagca
23761 ggaagcagag ctgtatcagc aggtgatgga atacgagatg gacactggtc
gctacaacat
23821 gatgtcttcc atcggtcgag actaatagag gaggtgctat gccacttatt
acgcaatcaa
23881 taaagaacct taagggtggc attagccaac agcctgacat cctgcggttc
tctgaccaag
23941 gtgaggagca ggtcaactgc tggtcatccg agagtgatgg cctacagaag
cgacctccga
24001 ccgtctggaa gaagagactc cagattgacg tagggtcagc ccctaagttc
cacctgatta
24061 accgagacga agtggagcaa tactacatcg tcttcaatgg gtcgaacatt
caggtcgtgg

24121 accttgaggg gaacccctat gctgtctccg gtgatatggg ctacgtgcag accagcaacc
24181 caagggatga catccgggtt atcacggtag cagactatac gttcatcgtt aaccgcaaga
24241 tggtcgttcg ggcagggacc gataagtctc atccgggcta caacacgaag aaccgggcgc
24301 tgattaacct gcgtggtggt cagtatggtc gaacactgaa ggtaggaatc aacggtggag
24361 tcaaggtgga gcacaagctg ccgaatggag ctaacgccga ggaggaccct cctaaggtcg
24421 acgcacaggc cattggtgcg gccctgaggg acttactggt agcggcttac ccgaactata
24481 cgttagccct tgggtccggc tacctgacta ttacagcacc tgttgggacg gaaatcacct
24541 cagtcgagac cgaggatgga tacgctaacc agctcatcaa cccggtgctg gatacagtcc
24601 agacagtttc taagctgcct ctggctgcac ctaacggcta catcatcaag attcagggag
24661 agaccaacag tagcgccgat gagtatttcg tggtgtatga ctccaacaca aagacgtgga
24721 aggagacggt agagccgggg accgtgattg gatttgacaa tcagtccatg ccacacgctc
24781 tggtccgtca ggccgatggg tcatttgact tcaaggagat ggagtggtca ggacgtgggg
24841 ctggcaacga tgacaccaac ccgatgccga gctttgttga ctcaaccatc aacgacgtgt
24901 tcttctaccg aaaccgtctg ggattcctgt ctggtgagaa cgtcatcatg tcccgctctg
24961 ccggatactt tgcgttcttc cctaagtcgg tggcgaccct cagtgatgat gacccgattg
25021 acgtggctgt gagtcaccct agaatctcca tcctgaagta tgctgtgccg ttcagcgagc
25081 agttgctact gtggtccgac gaggtgcagt tcgtgatgac cagctctgga gtcctgaccg
25141 ctaagtccat ccagcttgac gttggctctg agttcgctct gggggataac gctcgaccat
25201 tcgctgtggg acgctcagtc ttcttctcag caccgcgcgg gtcgttcacc agcattaatc
25261 gttactttgc ggtggctgac gtcactgacg tgaaggatgc tgacgacacc actggtcatg
25321 tattgtccta catccctaac ggagtgtttg acattcaggg gaccgggacg gagaacttca
25381 tagtagtgaa tacctctggt gcctacaacc ggatgtacat ctacaagttc ctgttcaggg
25441 atggagtaca gcttcaggct tcatggtctc actgggagtt tgagcctaac gagcgagtat
25501 tggcgtccgc tgctatcggg tcaaccgtct acatcgttaa gcaacactct caaggtgtgg
25561 acattgagca cctgaagttc atcaaggagg ccacggactt cagaaccgag ccgtatcgac
25621 tccacgtcga ttctaaggct accatgcaga ttcctacgga cagctataac cctgagacgt
25681 tcgtcactct ggtgaacatt gggaacgcct acggtggcaa tgctccacca cggggtaact

25741 acttcctgat tgattctcag ggtgcctata cgtctcttgg tcttctggac ggtcagactc
25801 tggtcaacct gaaaggggat tggtcaggac gtgaggtatt cattggccgc tcctataaca
25861 tgcgctacag gttctctcgc ttcttgatta agaaagagga cgactctggg gtgcagactg
25921 aggacaccgg gcgtctacag cttcgtcgag cttgggtgaa ctatcaggac actggcgctc
25981 tgcgactgac ggtctccaac ggtcagcgtg agttcgtcaa catcctgaac gggtacacct
26041 tgggtcagca gactttaggc accagcaaca tcggtgacgg tcagttccgc ttcgcaatga
26101 acggtaacgc gatgaccaca agtcttgtcc ttgagtccga ctatccgacc ccagtgtcca
26161 tcgtggggtg cggctgggag gcttcatacg ctaagaaagc gcaacgagtc taacttattg
26221 attggcctat agattcacct taactatcac tatagggact ataggccctt taagttataa
26281 tactttaaga ggagacttta tgtatattcg taaagctacg gaatcagatg tccactactt
26341 tctctggcat ctttcagcag atgatgttaa tgaatgcaaa gcaaactacg ggtcaaccgt
26401 aggtctctct gaaagactgc ttaagcattt atctccgtca tctgtggttt taacgaacgg
26461 tgtaggcgaa gtgtttgcct atggcgggaa ccaaggggac aacgtatggt tcttgacttc
26521 aggtcaggtc tacaagctga gacctaaaga gaagagagag ttcataaagc gtatctctga
26581 gtacagggac ttaatgttag accaatacgg gaccatctgg aactacgtgt ggtcaggcaa
26641 taagtctcac attaaattct tgaagttgct gggcgctaag tttcaccctg aggtgactat
26701 cagtccggta actggtgagc gttttcaact attcacaatc tctaaggagg acgtatgtgc
26761 gaacccgtga gcattggtat gggcatcatg gcagtagccg gggccgctat gtccgcttcg
26821 agtcaggcga aagctgaggg tgccgccatt gacgctcaga accgacaggc tcaggagatg
26881 attaagcaga tgaactactc tgatgtcaac ctgaagatgc aggagcgaga cctcaaggaa
26941 caacaggtcg ctgagctaac cgagacgact ctgaacggta tccgtaatca gggtatggta
27001 cgagctgctg tagctgagtc tggacttgaa ggcaactcca tggaccgcat tgagcgggac
27061 gtagaggggc agacagtcaa agagcgtgca ggtatcaccg agagctatgg ccgtgactat
27121 gcggctatct tcgggaaccg tatttccaac attgagaata ccaagtccgc tatcaaaggt
27181 cagggtaaga ttctcaagac cagcccactg gctcatgcgc ttaatgtggc gaacgccggg
27241 atgcaagggt acgcaatggg taactcaatc gctggtgcct ctccgtctca gggcgcagcc
27301 ccaattagtg ctgctaaagg cacacctaca ggtcatagct aacaggagga ccaatggcta

27361 gtaatatcga atcagcatta gctaaccgga ctatgggccg tggtcgcgct
ccgggtaaag
27421 ctatcagtct ggactatcag gctgcaaacg tacaggggca gaccggggac
tctggtcttg
27481 ccaaggcgct cacggacttc gtgtccactg gcgctggcgt ctacaagcag
tttcaggaga
27541 aggccaagag tctcggagac gaaaggtcca acgagattat tcgtaagctg
actcctcagc
27601 agagacgtga cgccatccat aatgggacac tcctgtatca ggacgacccg
tatgcgatgg
27661 aggctctgag agtcaagact ggtcgcaacg ctgcctatgc ggtagacgat
gagattaacg
27721 tcaagattca gaacggtgag tttcgcactc gtcaggacat ggaggagtac
cgccaccagc
27781 gactgcaaga tgccgctaag tcttatgcgg aagaggctgg gattaacccg
gcagatgaac
27841 acttccagcg tggatttaac gcagacatca ccgaccgtaa cgtagccatc
tacgggtcgt
27901 tcaataagta tttctctaag cagtccgaga ataccgctat gctgaacacc
cgtgtcgagc
27961 tgaactcgtt cctgaatgac ggggacctga tgcggtcgcc cgaggctggc
aagtcgttca
28021 tggcttacat gcgtgatggc ctgaccactg ctgccatccc gtctgaccaa
agggcgactg
28081 aagtcatcac ccagacggta cgtgacgcaa tccagaagtc cggtggctcc
aacttcctac
28141 agcaggtacg aaacgaacgc atcacgctta acggtgtgga cgcgacagtc
gaggagattg
28201 ttggacctga ggtattcaat gctgcaatga ttgaggccca aggcaccgag
tacaagctgg
28261 tagccaagta tcaggaagac ttgtcgcttg gcgttcagtc tgcgatgctt
caggatgacc
28321 cgaccatcgg tctggctcaa atccagaaac tcaaggctca gaacaaccag
cttcagccgg
28381 gtgaggagat gacgccccaa cgtcagatgc ttatcaacgc cgaggccagc
ctcttagagt
28441 ccgtgaagcg taagtctgct gaacaggcga aggagaacac taagctcatc
cagacgcaga
28501 acaagcaact ggtcattgac caagtgtacc agcgtcgact gaatggcgac
aacgtgtcca
28561 ccaactatga ggacctcccg gtctctgagg ctactggtga gttcaagcgt
tcggacatga
28621 acaactacgc tatgggtaaa ctccagcaaa tcgaccagat ggacatcccg
caagctgcga
28681 aggatgctca gaaggtggcg ctgctgcgag ctgacaccaa taacggtccg
ttccgtaacg
28741 ccttccagac gctgactcaa gatgctgctg gtgagtggca agctgccgtc
atccgtggtc
28801 agtatgaccc agacaagatg cagcgcttcg agtctctgcg taaggtctat
actcaggacc
28861 cttccagctt cgcggctctc taccctgacc aagcctctct gttcactact
ttcgagcaga
28921 tggacaagat gggtctggac cctcagacga tgattgacgc tgacaaacag
gctgcaagcc

28981 agtcccgtga gatgcgcatt gagtctgaca aggcgtggca agagctgaag aacgactcga
29041 agaacaagga cctctcgcgg ctcccgacga gtctggacgc aagtgctcgt aaggtctggg
29101 actcatggta ttaccgtaca ggtaacgctg atgcggcgac ccagaacacc cagaagtggc
29161 tgaacgagaa caccgtgact ttcagtgacg aaggtcagga cggtaagtcc atcggcatgg
29221 tgtccaaaca tcaacttatg gtaggggata accctgagtc atggcaggtg ggtcgagaca
29281 ttatcgacac cgctcgtcaa cagctcatca agactaaccc ttgggtcgtg aactctcagc
29341 tctccgtagt tgagcagaac ggctcggtct tccttcagga cgctaccggg actattcgta
29401 ttcgatacga taaagagctg gtaggcaaac tgtaccgtga acagcagaag caagccgagg
29461 ataaagccta cgctgaggcc gaacgtaaag ccaacacccg cgctcgcatt gtcgagacta
29521 aagcggctgg cgataagcgt cgtaacgagc gtgaagctaa tatccagaaa cggggcggta
29581 tgtacaaaga cgtatcgctg gagggtatcg caaacacctt aatcggtaag gagtaataac
29641 aatggcgact cgtggtattc gcaacaataa catcgggaac attcgtgttt ccaaagacca
29701 gtgggaagga gctacaggag acgatgggtc tttcgtaacg ttcgacactc ctgactctgg
29761 tgtccgtgct ctggcgaaga acctgatgtc ctacggtcgc caaggttatg actcaatcga
29821 gaagattatc actcgttggg cacctcctag tgagaacgac acccagtcct acattcagtc
29881 cgtggcttcg gctacgggca tcccggctac tcaaagtctc gacctgacgg acccggacgt
29941 cctcgcctct ctgtctgagg ctatcggcta ccatgagact ggctcccgct acgataagtc
30001 tgtctaccag actggcgtgg gccgtgctct tgggaacggt attaccccaa agtctccacc
30061 agtaagcgct aacgtcttcg acgctctcac ggagggcttg aaggcgaaac ctaaagtcgc
30121 tctgggagag aaccttccgg gcgttactgg tctgaacatc gaaggtcaag aacctgaggc
30181 tcccaatgag tctttcggag agatgttcta taagtcaact ggcgagacgc tggaccaacg
30241 ggctgaccgc tctacgtggt tcggcttcgg tggagctgct gaggctgaag tgaataactc
30301 tatggtcggc gtggctatcc gcgctggtca gaccgaggac tcactggatg tcattggcga
30361 cgtattcaac ccgacccgct ggaacaacca caagtggtcc cgcgaggagt tagaccagat
30421 tcgtaacgct ggggtcctgc ctcagtatta cggagtcatc actggtggtt cacctcagaa
30481 cctgaccgag ctgattaact tggcgctgga gaaccagaag ttagacgctg agaaggctaa
30541 ggccgggact ggcgctcaac tggcggctgg tgtgattggt gctggtgtgg accctctgac

30601 ctacgttcct atcgctggac aggtaggcaa gggcggtaag ctcatcaaca agatgttcac
30661 cgtggctgct cagtctggtg cgcttgctgg ggcctccgag ctggctcgta cttccgttgc
30721 tggtggcgat gctcatgtgg ctgaggctat tatgggcggt gctctcttcg gtggcgggat
30781 gactgctatc ggggacgcta tcggcaaggc cctcggtaag tcgaccaatg agttcgctgg
30841 tccagctacc cgtctggagg cccgtgagac tgcccgtaac gtcgatggtc aggacctgtc
30901 ccgtctgcca atccgcgaag gggaggagac cttcagtcac caaggcgtta agttcgctga
30961 cgtgccgaat gagccgggaa gtgttcgact ggaagacggt tcaatcctga ttggtgagaa
31021 tcctctgaac cctaagacac gtcaagtctt cgacgaggtg attgagcctg aacgtgcggc
31081 tgctggtgtc aaccttggtg gtctcaccga gattggcctg aagctcctga ggtctgagaa
31141 ccctgagatt cgcggacttg ccgctgactt ggtgcgctca cctacaggta tgcagtcagg
31201 tgcgagcggt aagattggta ctacagcgtc agacgtattc gagagacttc gtgcagtgga
31261 ccatcggttc tacaacgaca tcgacgatgc ggtgactcag gccctgaagg acccgtactt
31321 ccagaccaac tttaaccgtg acactggcgc attccgtcag gacatctacc agcgtgtagc
31381 tctggcgatt gaagatggaa gtggaaacct gaaggctgag ctgactccgg gtgaacttaa
31441 ggtctatggc ctgctgaaga accagttcga cgctaagcgt gagatgatgg agaacccggc
31501 gatgttcggt cgagtggacg ctcagtctat cttccccggt agccgcttca agggtactta
31561 cgtgcctcac gtctacagca accagatgaa gcaactgttc atcaacgagc tgggaagccc
31621 tgaggctcta caggaggcca tcaagaagtc gtggctcacg agctatgctt ccagacctga
31681 ggtcaaggct cgcgtcgatg aggccctgct ggaggctaac ccgaacctga agcctgagga
31741 acttgcggct gcggtcgata agtatgccaa cgacaaggct tacggtatct ctcacaccga
31801 cctgtttgaa cgctcgtctg tcatggagga gaacatcaac ggtctggtgg gtctggagaa
31861 caacaacttc cttgaggccc gtaacctgtt cgatagcgat atgtctatca tcctgcctaa
31921 tggtcagccg ttcaacgtga acagcctccg tgagtgggac atggacaaga ttgtcccggc
31981 gtacaaccgt cgagtcaatg gcgatattgc tataatggct gggaccggga agaccactaa
32041 ggagatgaag gacactgtag agaccatgat gaaccgtgct ggcgatgacg gtaagctgaa
32101 aggcgaagtg gcgactctgc gtgataccct gaagattctg actggtcgtg ctcgacgcga
32161 tggtgctgac gatgcggctt tctctaccgt gatgcggacc atgacagacc tgtccttctt

32221 cgccaagaat gcctacatgg gcgtacagaa cttgacggag attggcggga tgctggcccg
32281 tggtaacgtc cgtgcgatgc tgcatggtat tccgatgttc cgtgacctcg ccttcagaaa
32341 caagaagatg ggagcctcgg agattaagga cctgcacaac gtggtcttcg gtaaggagct
32401 tgacgactca atccgcccgt ccaaacagga tgtcatcgac cgtctgcgag cgtacagtga
32461 cctgagtaaa cctgtagcta ctgctttagg ttctgccaag tattacactg gtgaacttgc
32521 ggtccgctct ccgtttacga aagtcctgaa cgggacgacg aactacctgt tagacgccgg
32581 acgtcaaggc ttcttgtctg acatcgtgga acatagtctg actggcagta agcgtaagtt
32641 cgatgaccgt tggttgaaga ctgctggtat ctccgctgac cagtggaagg gcattaagtc
32701 cctcatccgt gagtcggtga ctcgtggtcc agacgggaag tacaccatca aggacaagaa
32761 ggcgttcagt caggacccaa gggctatgga cctctggcgt atgggtgaca ccatcgctga
32821 tgagaccctg cttcgtccgc acaagctgag caacatggac gccaaggctt atggtccgct
32881 ggcgaagact gtcttgcagt ttaagaactt cgtcatcaag tccatcaacg gtcgaaccat
32941 gcgtaccttc tacaacgcca ccaagaacaa ccgagctatg gatgctgcac tatcaacggt
33001 tatgtctatg ggtctggctg ggatgtacta catggctcag gcgcacatca aggcttacgc
33061 tatgcaggac ggtcgtgacc gtgagtacct gaagcaagcc ttgaacccga cgatgattgg
33121 ttatgctgct ctatcccgta gctcccactt gggtggacct cttggggtcg ccaacattct
33181 gggtggtatc gctgggtatg aggacactaa gttactccgc tcgtctatcc ttccgcgctc
33241 acctaccgag aagcctgaac gtgccattac ctatggtgct gcgaagagtg acccggtgat
33301 gaatgtggtc ggcaacttcc ttgagcaggt cccggctttc ggttatgctg ctaacgttgg
33361 cgcttcggct tacaacttgg ctggctacct gaagtccgac acccgtgtga acgaacgaga
33421 ctacatgacc gggatgtata acaccttccg tgagctggtc ccgaacgacc ctatcaccca
33481 gaagctgctg ttaggcacct ttgaggagca aggtattcac atcaagaact aaactatcac
33541 tataggaacg gaggcttcgg tctccaccta ttttgaataa caggaggtat aatggctaaa
33601 acaactatca ctcagttccc tgctggtcaa tcgcagtatc gaattgagtt cgactatctg
33661 gctcgacctt tcgtcgtcgt aacgttggtg aactccgccg accagacgca gaacagggtg
33721 ctgagggctg gagctgacta taggttcctg aaccccacct taatcgaggt tatgattcca
33781 cagacaggtt tcgatacctt gcaaattcac cgtcagacgg acaccgagct gattgttggt

33841 ttcagggacg gctcagttct tacagctaag gacttgacaa atgcagagct
tcaggccatt
33901 cacatctcgg aagagggtag ggaccagact gtagacttag cgaaggaata
cgctgacgct
33961 gccgccaaag cgcgaaacga tgccgaagac gcccgtgata gcatcgagca
ggtcatgaag
34021 tctggactgt acggatacac tctggttgag gacttccaaa aaggtgccac
attgtctcac
34081 ccggctgagg cacttcgctg gacactgcct gatgggacgg gagaatacta
ccgctgggac
34141 ggtgtgtttc caaaggtggt tccagcaggg tcaacgccca catcagctgg
aggggtcgga
34201 gttggtgctt gggtatctat tggtgacgca tctcttcgag gaaacctagc
taacccagcc
34261 tacggtgacg gccttgttgc ttcgcagctt cctaactccc cggtggctag
aacagtccat
34321 gataaaatgc tagaagctgt gagcattgcg gactatatgg ttggcggtga
tgtggctggt
34381 gcgataattg gtgccctgtc ctccactgct ggtcgggtta gtgttcctgc
cgggaatcac
34441 atagccacac cttccgcagc gcaagtggct ggcgtactct ctgccctaag
taggctgaac
34501 attaacgggt ccttaacgat tcgtctccct aagggaaggg ttaatctatc
ctctccggta
34561 ttggtggagc tggacggagg gaataaccta agcattgaag gacagcctaa
cgtcccagta
34621 accattacag gacaagcgtc tgtctctggg tctgctggaa actaccaagt
aacccttaac
34681 gtgtcctcga cagccggggt ctctgtgggt gacttcctgc ataccaacca
agctacaggc
34741 acaggagcat gcgaccttca tcgcggtgtt tgggagatta ccgctgttgg
ggcagggtct
34801 ctgactgtca ggaatacgtg ccagctctca tccttcccgg ctaacaccat
aacctccagc
34861 tctagccgag tgctgacctc agtgctcatg tttgaccgct gcgatgggtt
catagtgccg
34921 tcctctgaag ttggtaacat gagtaacttt gttatcgcag gtaactcaga
ctcatactgg
34981 agggcctcgg ctgtggggac taccgagctg gggactcatg gtctggctgt
aggctcaaac
35041 actgtagccg tgaacggtaa gtcagataac gttaaccctc aaggtaagac
gggcggtagc
35101 gtgactttcg gtcaatacat gggcgtctcc gggtttgacc agcagggcat
tgttaccgag
35161 cttggtggta gcttctgggg cgactttact tgctgctgta acaacaagag
acgcgggttc
35221 tactcgtcaa ctgcatctgg cattcgtgct aagcagatta cggccaatgg
taactatctt
35281 gatggggtta ttgcggatat tggcggagac atctactcga gctcttctag
ctgcgccgca
35341 ggtaacgggt cctcaggcat aagcgctgcc cataacggtt ctgtgatatg
ggacgcaggc
35401 aaggccagct acaataagct gaatggtgcc aatggggtgg cgggggcctt
cctacagatg

35461 actggctcaa cgatgcaggg taacttggcc actggcgcta acctagccta
tggtgccatc
35521 ctctactgcg acaactccca gatatcttta aacggaacct atggcattaa
ctgtcagctg
35581 ggctcggttg ttcgtggtcc taactgcacg tatataggga ataacaacca
aggcattaga
35641 ggctcttaca atgcgaccgt aacctttaca ggctctacat tctctggtaa
ctctggcggg
35701 gacttcctct ttaccgctat gtctcttggc atcttcggga acactaatta
cggtggggat
35761 attgttgcca cggacattaa gttagtgaac cagtcaactg gcaaaggtgt
ccgtcttact
35821 gggacctctg gtggggataa cattgttatg tcctatgatg tcacgggtaa
tgggtccttt
35881 gtcgagggtt ataacttcag gtctggtgac gttggtatct acccatcgga
tgatgcggtt
35941 cgcaatattg gacgaccagc aaacaggttt aacatcggct tctttgcggg
aggaacacag
36001 tctacgtcag acgctaggct taaggaccct atcagggact tttctgaggc
agaactcaag
36061 gcggctgtcg cctgtagtaa atccttagga ttctggactt ggctggacga
cgacagcaag
36121 cgccttcatg cagggactac tgttcaaaga gttctagaaa tcctagagga
taacgggctg
36181 gattggcgtg agtatgggtt catcgggttt gactcgtggg aagatgagta
taaacctgtc
36241 gttgcggaga ttgatggtat ggaatacgag actggcgagg ttgtcaaggt
ggttgaggca
36301 gggtcactgt ggcagctgcg tgaccaagag tttgacagat tcctaatccg
tggactctcc
36361 gcccgactct cggctatcga gaaggactga ccagtcgctt aacaactttg
ggtcaaggac
36421 ggcccgttta acaatggagg tagtatgatt gagttcgact tcaagaatga
ggtcctcaaa
36481 gcctcgccta tcgtcgggac cgctgcggct gatggtgcca gtcggttctt
ctttgggtta
36541 acgctcaacg aatggttcta cgtcgctgct atcgcgtaca ccgtggtgca
gattggcgtc
36601 ctaatttaca agacgattaa gagcggaggt aagacatgac gcagatggac
ttagagaagt
36661 tcctgttaat gctggacact gaacgtgctc gactcatgct gcaagacctg
cgggatgact
36721 ctaagcgctc gccccagctt tacaacgcca tcgagaagct gttggcccgt
cacaactttg
36781 tgttgagcaa ggtgtcggtg gacgagaagc agttggctga catggaggct
ctgaacagag
36841 agtacgacaa ggtgctatca gctgccgaag ataatgacac cgggtatggt
gtccaataag
36901 tgttagactc aaggtcatct actacatata gtgggtggcc tttatgatta
acactatgcg
36961 agatgcgctc tcgtgaaatc tgagaaacaa ggagggcaac tatgctcaaa
cttttacgac
37021 aggccgtccc ttggctggtg gcaggactcc tgttctgttc tggctactgg
gtggcagaca

37081 ataagtggga ggcgaaggta aacaatgagt acatcacgaa acttgaagca actgagcagt
37141 cgaggcgttc agtgcagtcc gaagtcaaca aagtctcagc cgagtggcag gacaagatgt
37201 ccgagctgga aggctctact gatagggtta ttgctgacct taaccgcgat aacaagcggc
37261 tgcgcgtcaa agtcaacacc acaggtatca ccgagtcaga ttacagtcga tgcttccctg
37321 atggtcgagt cgaactacac ccagagactt ctaaaagtct tatccgaata acgcaggaag
37381 cagaccttaa ggagaaagcc cttcaggata ccatacgtaa gcttcagcaa gagaaggagg
37441 cgaaaccttg agtcaagact tagcggcgcg tcaggcgctt atgactgccc gtatgaaggc
37501 agacttcgtg ttcttcctgt tcgtcctgtg gaaagctctg tccctaccgg tcccgactcg
37561 ctgtcagatt gacatggcga agaaactatc ggctggggac aataggcgct tcatcctaca
37621 ggcgttccgt ggtatcggga agtccttcat tacgtgtgcc ttcgtggtct ggaagctatg
37681 gaataacccg gacttgaagt tcatgattgt gtcggcctca aaggaacgag ccgatgcgaa
37741 ctccatcttc atcaagcgta tcattgacct gatgcctcag ctccaagagt tgaagcctaa
37801 gcaggggcag cgagacgcag taatcagctt cgacgtaggg cctgccaagc ctgaccactc
37861 accttcggtt aagtccgttg gtatcactgg tcagttaact ggtagccgtg cggacatcct
37921 gattgccgat gacgtagagg tccccggtaa ctcagcgact caagctgcaa gggaccgact
37981 gtctgagctg gtgaaagagt tcgacgcaat cctgaagccg ggtggtacgg ttatctatct
38041 gggtactcct cagaccgaga tgaccctgta tcgtcagctt gagggtcgtg gttactcgac
38101 taccatctgg ccagctcgtt acccgcgtga tgagaaggac tggaagtctt acggcgaccg
38161 tctggctccg atgcttcagg ctgagcttga gtccgaccct gagggctact actggctccc
38221 aacggatgag gtccgcttcg acgatgagga cttgaaggaa cgtgagctgt cctatggcaa
38281 ggctggcttc gctctccaat tcatgctcaa cccgaacctt ggcgatgccg agaagtaccc
38341 gctgaagctg cgtgacctta tcgtagcgga cttggaccct gagtctagcc ctatggtcta
38401 ccagtggctc ccgaacctcc agaacaagcg tgatgacgtt cctaacgtgg gactcatggg
38461 tgacacctac cacacgtatc agactgtagg ttctgctttc agttcgtaca cccagaagat
38521 tctggtcatt gaccctagtg gtcgtggtaa ggatgagacg ggttatgcgg tcctgtatca
38581 gctcaacggc tacatcttcg tgatggaagc tggtggtatg cgcggtggct atgaggactc
38641 aacgctggag gctctggcga agattggtcg caagtggaag gtgaacgagt acgtcattga

38701 gggtaacttc ggtgatggta tgtacctcga actcttcaag cctgtagcgg cccgtattca
38761 cccggcagca gtgactgagg tcaagagcaa gggtcagaag gaactccgca tctgcgacgt
38821 tctggagcct atcatggggt ctcaccgact catcgtgaac tcctcgacca ttgtgtctga
38881 ctaccagact gctgctgaca aggatggagt ccgtaaccct atctactctc tgttctacca
38941 gatgacccgt atcagccgtg aacgtggagc cttggcacac gatgaccgac tggatgctct
39001 ggctatcggt gtacagttct tcgtagagtc tatggctaag gatgccgtga agggccagcg
39061 tgaagttacc gaggagtggc tggaggaaca gatggaagac ccgaagaagg gctttaagtc
39121 catcgagacg gactactggg ccaatggggt ccgggtacag ttcaatacag atgatgactt
39181 gggcttaggg tcatacgttc agttccactg acctaatgaa taactatggg tgaatactgc
39241 atgaataagc ggttagtaac ccatagttac tagtggtcta actctatgat tctaaaggct
39301 tttggactta actatcacta tagggaagac ccccggttac ttatagtaaa gactcaatga
39361 ataagcatat gcacacttta tgcaagacct taggaggcag actcctgagt tcttacctaa
39421 ggttcgcacc gatagaggag ggtgataata atcataatcc ctccataact taaagtagac
39481 catcgctgac aggaggtatg tagcatgggt aagaccaaag ctgttctcaa agctctggcg
39541 accaatagag ctacgtacag gtttcttgct gctgttctac ttgctgctgg cgttactgct
39601 ggaagtcagt gggtcgggtg ggtcgagact ctcgtatgta ctctggtgtc tgagtgtcat
39661 taacgccata atggtaacaa tcaacgaaag caagacctaa ggtcagttca tagctgacgc
39721 tactctactg accttagcta ctgaagtcaa gacctacagt caacactata gaagctatgg
39781 tctctcaggg tcgttaggac ctacggtctg acctacggat gggcctcctt aagagggacg
39841 atgagttttg gaccaaaagt tcgagagtcc atctcacagt tcaagaacct caagtctccc
39901 catagggtca ctctcagtcc gactcaaggc cctacccata gaccttcggt ctaaacctca
39961 agtcattgac ccgaagtcgc cccgaagtct ggactgatag tgcctatatc ttgtggttgg
40021 gacgattggt cgggactata tgtagtgcct ctgagtctct
//

## Supplementary GeneBank File S2: Genebank file for IMM-001_Rtp_phage

```
LOCUS       IMM-001_Rtp_phage      32486 bp    DNA     linear   PHG 10-
AUG-2017
DEFINITION  Siphoviridae isolate IMM-001.
ACCESSION   IMM-001_Rtp_phage
VERSION
KEYWORDS    .
SOURCE      Siphoviridae (phages with long non-contractile tails)
  ORGANISM  Siphoviridae
            Viruses; dsDNA viruses, no RNA stage; Caudovirales.
REFERENCE   1  (bases 1 to 32486)
  AUTHORS   Chakraborty,S., Begum,Y.A., Qadri,F. and Camilli,A.
  TITLE     Genomic analysis reveals CRISPR-Cas mediated host-pathogen
            interaction between enterotoxigenic Escherichia coli and
phages
  JOURNAL   Unpublished
REFERENCE   2  (bases 1 to 32486)
  AUTHORS   Chakraborty,S., Begum,Y.A., Qadri,F. and Camilli,A.
  TITLE     Direct Submission
  JOURNAL   Submitted (10-AUG-2017) Department of Biochemistry and
Molecular
            Biology, University of Dhaka, Dhaka-1000, Dhaka Dhaka-1000,
            Bangladesh
COMMENT     Bankit Comment: TOTAL # OF SEQS:1.

            ##Assembly-Data-START##
            Assembly Method       :: Cap3 v. January 2014
            Sequencing Technology :: Illumina
            ##Assembly-Data-END##
FEATURES             Location/Qualifiers
     source          1..32486
                     /organism="Siphoviridae"
                     /mol_type="genomic DNA"
                     /isolate="IMM-001"
                     /isolation_source="Environmental water"
                     /host="Enterotoxigenic E. coli (ETEC)"
                     /specimen_voucher="icddr,b_IMM-001"
                     /db_xref="taxon:10699"
                     /country="Bangladesh"
                     /collection_date="2010"
     CDS             25..105
                     /codon_start=1
                     /transl_table=11
                     /product="hypothetical protein J132_04738"
                     /translation="MVFLIVAEFAIIDVLVNECWKNYHTS"
     CDS             complement(67..159)
                     /codon_start=1
                     /transl_table=11
                     /product="hypothetical protein"
                     /translation="MNIQKLHVYRKMAQPESKSTCMVVLPTFIH"
     CDS             78..164
```

```
                     /codon_start=1
                     /transl_table=11
                     /product="Uncharacterised protein"
                     /translation="MLEELPYKLILILAEPFYGIRVTSVYSL"
     CDS             359..496
                     /codon_start=1
                     /transl_table=11
                     /product="glycosyl hydrolase family 9"

/translation="MTLVRIKRHIYRKSHQKTVHSGYGKVTSSNVVALETEKCINDKI
                     Y"
     CDS             complement(398..505)
                     /codon_start=1
                     /transl_table=11
                     /product="potassium transporter TrkA, partial"
                     /translation="MYILVYFIIDTLFSLQSHDITGCNLAVSAVYSFLM"
     CDS             447..545
                     /codon_start=1
                     /transl_table=11
                     /product="PREDICTED: cyclin-Y-like protein 2"
                     /translation="MSWLWRLKSVSMIKYTKIYIDRHVNLWHCGNH"
     CDS             complement(493..603)
                     /codon_start=1
                     /transl_table=11
                     /product="hypothetical protein"
                     /translation="MTSPSVTFCISMPRCCVLRFSDYHSARDLRVGQCIF"
     CDS             575..1123
                     /codon_start=1
                     /transl_table=11
                     /product="hypothetical protein"

/translation="MQKVTLGEVIREMVRKAIESESGEFKVPVSQIFKMIRGKPYPEM

EYDGETDEILNLADRSLPELKNSYIYNTVSRMTELRDANKRARYKFIWIDDEGEQTSA

RQFDGDGADKHLVIYIENGAHWTGNREKKKQEAEKEAATIEKFKKRLLAVTPSIIHLS
                     GEKLEGALFALSAYQEMIKEAK"
     CDS             complement(781..1146)
                     /codon_start=1
                     /transl_table=11
                     /product="hypothetical protein"

/translation="MVVYCHTCYLASLIISWYADSANRAPSSFSPLRWIMLGVTAKSR

FLNFSMVAASFSASCFFFSRLPVQCAPFSIYITRCLSAPSPSNCLALVCSPSSSIQIN
                     LYRALLFASRNSVIRDTVL"
     CDS             1125..1211
                     /codon_start=1
                     /transl_table=11
                     /product="hypothetical protein"
                     /translation="MYGSRLPYHLRYGEEEVEVAMEFYRKAK"
     CDS             1129..1218
                     /codon_start=1
                     /transl_table=11
```

```
                     /product="hypothetical protein [Bacteroides
fragilis]"
                     /translation="MAVDYHTTCGTVRKRLRWRWSSIGKLNNS"
     CDS             1256..1543
                     /codon_start=1
                     /transl_table=11
                     /product="hypothetical protein"

/translation="MDGIITTSTARWSTKIKMAHQIEVMSEINDLLLTLVKIIDRPVV
                     SYLVGFEEIFIEFYKQPNSPCFGVVSETYRFKSCSELRDCVNSLTSSTTDC"
     CDS             1603..1833
                     /codon_start=1
                     /transl_table=11
                     /product="phosphodiesterase"

/translation="MERNKYEREIIGINGERAIVDVYRTLSAFSVTDPACQHAIKKLL
                     CMGLRGHKDVITDLDDAIDSLNKMKTYLEQTK"
     CDS             1830..2132
                     /codon_start=1
                     /transl_table=11
                     /product="hypothetical protein Ec3a_06"

/translation="MITINLSDKQAATLKQLLSSQPMRCSAVEFSGMVKDVCTQISNQ

QFDVSDFESSRQYESLSIESVGLTQKTELYKSIGWIIKESSQNEGYLTNWDDETNK"
     CDS             complement(2084..2179)
                     /codon_start=1
                     /transl_table=11
                     /product="hypothetical protein LY90DRAFT_513601"
                     /translation="MLNAIKLTCVISVNVNHLLVSSSQLVRYPSF"
     CDS             2129..2275
                     /codon_start=1
                     /transl_table=11
                     /product="gp65"

/translation="MIYIHTYYTGKFNSVKHVRVYEDIDEVLGQCRVLGGDIKICRLI
                     QVIL"
     CDS             complement(2260..2379)
                     /codon_start=1
                     /transl_table=11
                     /product="hypothetical protein"

/translation="MPTSIILTFISLLHLVVDVLIMPDCSGDCLAIRAIYKIT"
     CDS             2315..2560
                     /codon_start=1
                     /transl_table=11
                     /product="methyltransferase type 11"

/translation="MINTSTTRWSREMKVKIIEVGIAVDDELSLADCGFQVGDVVEVS
                     GQYKDGNLSVKAIRETNFVSIGNEVSIQEGEYEVIEE"
     CDS             2557..2736
                     /codon_start=1
                     /transl_table=11
                     /product="hypothetical protein [Escherichia phage
```

```
                     vB_EcoS-IME253]"

/translation="MKFKQSNPKLQANHFDGYSTFIIAQACGMRDEPQVHTIHLTEEQ
                     ARELAYEILEQLNKI"
     CDS             2746..3111
                     /codon_start=1
                     /transl_table=11
                     /product="HNH endonuclease"

/translation="MKLTKLQRDELKFKLGGFCAYCGCELGDKWHADHMKPVIRYDGK

MVHDELDNISNLVPACHACNLHKHCNSVEDYRRIIDDGRREFLASGKGKALVRMGLVD
                     MKMDDIVFWFEKFEKLNKI"
     CDS             3197..3325
                     /codon_start=1
                     /transl_table=11
                     /product="hypothetical protein"

/translation="MALLKHGDVFCSWTVSSDGNYYIIQNKNGEEIYLSKDEVALY"
     CDS             3325..3456
                     /codon_start=1
                     /transl_table=11
                     /product="gp68"

/translation="MDNYESPSQWAKRMMDEAKTGEESMQYFELMNLWKEREDKKNA"
     CDS             complement(3377..3628)
                     /codon_start=1
                     /transl_table=11
                     /product="hypothetical protein"

/translation="MTSLPLLFSNLCYLHRSLYQKLLKLRCYHRRTQTCRRIYRESKL
                     NPQQTPDSSEKARVKHFSYPPALSIDSLARSIALTLRQS"
     CDS             3663..3902
                     /codon_start=1
                     /transl_table=11
                     /product="hypothetical protein Ec3a_12"

/translation="MFGLNEAHFNAVKKQAKKLNDDYSKLSPKERKNDKLVAALISGL
                     WEPVFTIISRDRFVWVAGYLKGRVGHDENGNSLYE"
     CDS             complement(3792..3968)
                     /codon_start=1
                     /transl_table=11
                     /product="hypothetical protein Ec3a_12"

/translation="MRRNNLYLCLCRIPTLNINDLILFIKAIPILIMANATLQISSHP
                     NEAVSTDNSKYWLP"
     CDS             3871..4134
                     /codon_start=1
                     /transl_table=11
                     /product="transcriptional repressor"

/translation="MMRMGIAFMNKIKSLMFKVGILHKHKYRLFRRINWYEDEYQDGC
                     RTKLICTVCGKIFNSKVDLMVCKSTLDFIRYEKEIASLRGFNG"
     CDS             4094..4225
```

```
                     /codon_start=1
                     /transl_table=11
                     /product="30S ribosomal protein S4"

/translation="MKKRLHHLGVLMDKFIECVVYTLCITLVFCSLMISFGVLFRII"
     CDS             complement(4241..4327)
                     /codon_start=1
                     /transl_table=11
                     /product="hypothetical protein"
                     /translation="MDFVFRSVRRCHLVAFCATRYILILSGQ"
     CDS             4347..4472
                     /codon_start=1
                     /transl_table=11
                     /product="hypothetical protein"

/translation="MAKSVEKLDAGCVTSNLSIAKSLLKDLFCWRNRITQRPPKS"
     CDS             4519..4701
                     /codon_start=1
                     /transl_table=11
                     /product="DUF3667 domain-containing protein"

/translation="MWDVKLSIRLMGRSCKNCQQEFIASVAAESSEEAVAKVKVMSGA
                     DPDYHKFLVAYVRERK"
     CDS             complement(4631..4816)
                     /codon_start=1
                     /transl_table=11
                     /product="BnaA08g27020D"

/translation="MLMHPLIIVNTNTKKALKKRAKCRTTTREMKQPESRTSFTFAHE
                     RKLLGICGNQGRLPTLP"
     CDS             4858..5061
                     /codon_start=1
                     /transl_table=11
                     /product="hypothetical protein"

/translation="MMTLKAVYKDGSEQIIEVKRVGYVQGSRLVDFVDCDGPGQFFME
                     DRVLSVYVMNSAGSTVGKYQLAD"
     CDS             complement(5109..5282)
                     /codon_start=1
                     /transl_table=11
                     /product="hypothetical protein"

/translation="MSNSCSGVIIRFLASVVMSEYFLLYMALKFPELTFSCAVLLKFS
                     SFIFVLLLFRLAL"
     CDS             5143..5667
                     /codon_start=1
                     /transl_table=11
                     /product="terminase small subunit"

/translation="MKDENFSSTAQEKVSSGNFKAMYNKKYSDITTLAKNRIMTPEQL

FDIAVKYFSWAEDQAIKAIETASFQGVVTENLVHKPRVFTLTGFCLYCGVTHSAIEKW

RKSAGYDEVIAFIDSVIREQKYQLAASGIVNPGMISKELGIDKPQEINITSTSSANDV
```

DTMKEALESVISKL"
CDS 5684..7252
/codon_start=1
/transl_table=11
/product="terminase large subunit"

/translation="MSNLVWEEMTSAEKMAVKAISTHSFEGFLRVWFQLTQGERYIPN

WHHKYLCRIIDEIIAGERKDTIINVAPGSGKTEIASIHFPAYSMVKLKKVRNLNISFA

DSLVKRNSKRVRDLIKSVEFQELFPCKFGTCKDDELQVLDESGKVRFESISKAAGGQI

TGARGGYITDSYSGAVLLDDFDKPADMLSAVFRANNHVMLKNTIRSRRASSVKGKATP

IISIQQRLHVNDSTWFMMNGGMGISFDLIKIPALVTEDYVDTLPDWIKQQFIDDVLSS

EYIERDGVKYYSYFPEKESVNDLVAMWDSDSYTFLSQYQQEPVALGGNLINVEWLQRI

SDTLRPPAKYDYRFMTLDTAMTTKSYSDFSVLQLWGYKDGKIYLLDQRRGKFEAPELE

AELLDFEKIARASNQTDGILRKIIIEKKASGIGLIQSVGRVMRTPIEPYVPDNDKLTR

VMSALPQIKAGNVILPESATWLNGLLTEIAAFTADDSHVHDDQIDCLTMAVNLVLNIA
EDPKSRMMRLAGIK"
CDS 7428..7532
/codon_start=1
/transl_table=11
/product="hypothetical protein"
/translation="MTASVLLMMMVVIFTSITEHSTSFESTMRMATSI"
CDS 7754..9025
/codon_start=1
/transl_table=11
/product="gp56"

/translation="MVKADSYATQFLGGASDGSEIYGHFGNTYLTPPFADSLYVSNSI

VRRIVDTIPETAIAAGFHIDGIDDEGEFWSRWDYLKVNESISDAWSWARLFGGSAIVA

IVKDNRALTSPVREGAELESLRVYERSQVKVQTKEENPRNVRFGKPLTYRITPEGSTM

FYDVHYSRVHIMDGEKIPSSLRRGNDGWGASVLTPDLLESIKDYQTCEKLATQLLRRK

QQAVWKAKGLAELCDDAEGFGAARLRLAQVDDNSGVGRAIGIDAEAEEYSVLNSDIGG

IDGFLDKKFDRIVALSGIHEIILKGRNVGGLSSSQNTALETFHKLIDRKRNEELLPVL

GFLIPFITTEQEWSVEFNPLAQESKKDRAEILKNNVDSISALITNGVMDTDEARDTLR
ALAPEVKIGEGSIVTEQSAQEDLPKEEVVIE"
CDS 9070..9192
/codon_start=1
/transl_table=11
/product="beta-ketoacyl synthase, C-terminal domain
protein"

/translation="MDSSSTLQWWQGLACRFITWRMVLQFASSGQQRKYSKRNR"
     CDS             9126..10124
                     /codon_start=1
                     /transl_table=11
                     /product="capsid and scaffold protein"

/translation="MEDGSTVREFRPAEEVFKEESLASYQGKPMTLNHVFVNSDNAKD

VVVGSVTGKAEALGSTVIAPIVVYSQQAIDEARTGNAKELSVGYSAVLDETPGWGDPA

TGEYILRSDAKESDVPPDWQEFDAIQRDIKVNHLAMVSRGRAGVAKLNMDGQQENPYT

DNVDSIKEDVKEMVKIKLNGTQEFEIAPEVAAHIATIKKDADDAKAKADTIEAERDSL

KAKVDAIPAEIEKAVKAAKADAEALAALVTVAAEVGVKTDCLDAKGIKVAYVKEVSGL

DVADKSDAYIDAAFDIAKDSDKMAEVRKATAASDKSDKSDEPKKLDPRARLAKIKK"
     CDS             complement(9668..10345)
                     /codon_start=1
                     /transl_table=11
                     /product="hypothetical protein"

/translation="MRSNNNTKNVSGGNTSCSYYLVPINRLSGRNTNNSTNRNCSRAT

NYTSAIDIIQGGISYHTCQCYRTNCHYLSPYFLILARRARGSNFLGSSDLSDLSEAAV

ALRTSAILSESLAMSNAASMYASDLSATSRPETSLTYATLMPLASRQSVLTPTSAATV

TRAASASASALAALTAFSISAGIASTLAFSESRSASMVSALALASSASFLIVAMCAAT
                     SGAISNS"
     CDS             10136..10609
                     /codon_start=1
                     /transl_table=11
                     /product="gp54"

/translation="MAIRSVALAGMVADTSLYNIDGACVVGGSAAIPVGTVVGVTSAQ

PVDGHKVVAATGITAANVLGVVVRSHYETPDGTARVNEAVNVMTSGRIWVRTTLDAAP

AFGAPVLVSATGVVEEAGAVATGWTFAGGFIPNTGTKPQDLSVDGALVEVQVKQK"
     CDS             10572..10730
                     /codon_start=1
                     /transl_table=11
                     /product="hypothetical protein VDG1235_1290"

/translation="MALWLKFRLNRSNKYSSVNKPPSGGFFVIKCCLCYIQLSSKKNE
                     IKHGLHRS"
     CDS             10699..10926
                     /codon_start=1
                     /transl_table=11
                     /product="gp53"

/translation="MRLNMAYTEVKAISADSGRFNTLLQAAIDGGFQPVSKITVNGSQ

```
                     SYSIVVAKGADSSITEANDHLHQYAQAVRSC"
     CDS             10883..11398
                     /codon_start=1
                     /transl_table=11
                     /product="gp53"

/translation="MITSTNMRKLFEAVEAASDDGFTIDTSSLSLSSGQFFAYAYKGS

AGGSVSVAWGDVTGKPTTFAPIVGTGATQAMAGNKTLANIGGVVPLSGLPIASTTAAG

IIQIGTGATNAMPGNKFVAGAAVANVTAQTVTGADAEAVATSATTAVNEVATKLNDLL
                     AQLRVAKIIAG"
     CDS             complement(11348..11464)
                     /codon_start=1
                     /transl_table=11
                     /product="hypothetical protein"

/translation="MYESIIQHGDHKSTGELSASLALARDDLGDSQLCEKVV"
     CDS             complement(11418..11498)
                     /codon_start=1
                     /transl_table=11
                     /product="hypothetical protein"
                     /translation="MAILVYSFSVMYVRVNYTAWRPQINR"
     CDS             11491..12432
                     /codon_start=1
                     /transl_table=11
                     /product="gp52"

/translation="MAIKFDAEQAKITAHLEQMGVDKADAAGIWTVKQLTANLARAYE

AEYSENSVVNIFPVTNEIPSHAKYFEYPEFDGAAIAQIIAEYSDDLPLVDAFMTEKQG

KVFRFGNAFLISIDEIKAGAATGQSLSSRKQALAFEAHDNLLDKLVWSGSAPHGIPSV

FNAPNVNQVVAGTWNSAAAAMTDITALIDAIETATNGAHTATDILLPASARRLMQELV

PNTSISYAQLFATNNSGITLRYLQFLDNYDGANGKAALAFEKNPLNLSIEIPEVTNVL
                     PGQPKDLHFKYPVTSKATGLILYRPLTVSVLKGITYA"
     CDS             12508..12981
                     /codon_start=1
                     /transl_table=11
                     /product="gp40"

/translation="MEFSDYIVYDNGKLRWKKKTGRSTQIGKEIGINSGSGYLTFKFY

GKRYKVHRVVWMIMVGPIPDGMETDHINHVRDDNRIENLRLVSRIENMKNKSVYRSNS

SGFCGVSVKSSGMYHAYIQFNGKQVNLGLFKEKQDAVTARLEAEMVYGFHDNHGL"
     CDS             12953..13081
                     /codon_start=1
                     /transl_table=11
                     /product="hypothetical protein"

/translation="MDFMIIMGYNQPSINKSGATNDSFRTHWRVHDYLSWCELYAR"
```

```
     CDS             13014..13259
                     /codon_start=1
                     /transl_table=11
                     /product="Halo28"

/translation="MIRLEHTGACMITFRGVNYMPGEFIEVEEINAGLKSLIAEGRLQ
                     IDGDTKATKEIAAEVKAKSKRKEPKTIDEAQTGQEYK"
     CDS             13150..13353
                     /codon_start=1
                     /transl_table=11
                     /product="hypothetical protein"

/translation="MATPKQLKKLPQRLKQSLSAKNRRPLTKLRLVRNINNQMGASAS
                     VFAWGFYERTSIGIYEITGSSFA"
     CDS             13301..13696
                     /codon_start=1
                     /transl_table=11
                     /product="Halo29"

/translation="MNEQALAFMRSLVPALRNVPDETLDAWLELAKLYICASKFGDDA

YKALALYTLHIAFLDGALKQNGSLDDYGKKIASYSLSGEYSIRHESTSQTQSSMTATP
                     WGRLYWNLLRKKGGGFGFITSAGRGCGCR"
     CDS             complement(13589..13717)
                     /codon_start=1
                     /transl_table=11
                     /product="T9SS C-terminal target domain-containing
                     protein"

/translation="MILLGSNSSATAPTPSGRNKAKTATFLTQEVPVKPTPRGGCH"
     CDS             13693..14064
                     /codon_start=1
                     /transl_table=11
                     /product="Halo30"

/translation="MNYSQVESLMRTGINIFSDGDGTFTMETGKGGVEIVDGVEVPVA

GGTALIKGLVREIKTRDIDGEYIQFGDKRGIFTSEVPILQGYRIIVDDETYTVVDPRP
                     VKPTGTVVAYRPILRRIATYG"
     CDS             complement(14038..14178)
                     /codon_start=1
                     /transl_table=11
                     /product="hypothetical protein"

/translation="MALAALITVPDTTSDTLESALSIQSAILFVNVATPPMFLAIGSN
                     PS"
     CDS             14057..14494
                     /codon_start=1
                     /transl_table=11
                     /product="conserved phage-related protein"

/translation="MARNIGGVATFTNSIADWIDRADSNVSEVVSGTVIKAANAIVDL

SPVDTGRFKANWQITANSPAAQSLNEYDKTGGDTKRYLARQAQAVANSPATKVIYITN
```

```
                     RLDYASDLEYGASQQAPAGVLGVVNIRLGRYFEEAVAETRSKR"
     CDS             14494..14892
                     /codon_start=1
                     /transl_table=11
                     /product="minor tail protein"

/translation="MQYELSLAARRFVTDLVKTFPVRYPISYENVAFSPPSNGGMWLK

YDYTEADTVTYSLSRKCKYYVGMVQVSVFFSPGDGVDSARRIANQLAESMLDGTMLDT
                     GYIYEGGVVHPVVKSKSGWFIPVRFYVRLD"
     CDS             14905..15564
                     /codon_start=1
                     /transl_table=11
                     /product="major tail protein"

/translation="MAHLSNGTQVFVEGSRGEAINVTAISNAANPVFTVAASSGLAVG

DYILVTSASSTFMADKQLRVTAVSDTSVTVEGIDTSNVTKFPAGGTAEIVKILSWFEI

PCVQDVSTDGGEQQFVNFQCLSDDREQQIPTYKSAVTNTYTFAHEYTNPVYPILRNYD

EAGTVVAIRMFVPRASEMRLQSGTIAFNDTPSIGVNEIETVSIAVSIRGRLSSVASEL
                     S"
     CDS             complement(15609..15695)
                     /codon_start=1
                     /transl_table=11
                     /product="hypothetical protein"
                     /translation="MIFFTLFISLIYHFVSMNELYSNQHNSL"
     CDS             15673..15924
                     /codon_start=1
                     /transl_table=11
                     /product="hypothetical protein"

/translation="MNNVKNIIKLAAALGAFYLAIQIVNPTSGATANSDAIKYETKFN
                     SAYSYCEKVAGDKVNQIMTHGREVCTEVAHKIAKEDESK"
     CDS             15863..15955
                     /codon_start=1
                     /transl_table=11
                     /product="phosphoribosyl-ATP diphosphatase"
                     /translation="MVAKCAQKLLTKLQKKTNQSNIIIQHKQRR"
     CDS             15955..16050
                     /codon_start=1
                     /transl_table=11
                     /product="hypothetical protein"
                     /translation="MRWQNLNFHLRRFLTLNFQLSFSWLMVKMLK"
     CDS             15960..16274
                     /codon_start=1
                     /transl_table=11
                     /product="tail assembly chaperone"

/translation="MAKFKLSLKALPDFKLPVKFQLANGEDAEVVLTVKHKKSSELQE

ILNRENFSTKELISEICSDWDLEEPFNDENVDEFANLFPGAVPSLMDSYMKALAGQRV
                     KN"
```

CDS 16337..16588
/codon_start=1
/transl_table=11
/product="TfmS"

/translation="MKRSDYEGEEPEELFFDETMMQSWDVYCAMSTQWRVGACGATGL
DYNCLPFLFKVYKIDDEEMALNDIRILEAKALDMMNAKK"
CDS 16627..19599
/codon_start=1
/transl_table=11
/product="tail length tape-measure protein 1"

/translation="MATNEIAGITLAVDVAQVDRGTQSLQKFRQANEQAATGINSFVN

AELVAKTQARDTARQLEEQRKSFASLQQVIDPTAAKFQKLQKAATELDKAFESGLVPD

DEFFRLGSVIETQINKLNRSRAALSEEGRAALQASIDKEKATATAQKFIQSLENEANA

ATLTRTELLQLKAAQLGVSDQAAPIIDRIGKAAEASAVQLQKQSSAFVKSGLSAGQYK

QAIRQLPAQITDIGTSLAGGIPIWLIAIQQGGQIKDSFGGIANTARFALAAINPLTAV

VGTLGLALGALGVAAYQADKNTRGLASTITLVGNSTITSTAQINQVVDGIEKTTLATR

GLIQEIANSLVANGNLTLTQIDKITKATAQWATVTSTDSKTILGYFDQITKDPIKGLT

DLDKQFNFLQYGQLKYIENIRKTAGETEAVTAATELFAEVMENRLADVALSLNPLESA

WNSFRKFVSEILDDISKRTLGALNLITDVVAGTIEQIRSLINSGDILLGEFAIAATKT

LQSIPGLGDIGNEAIAEQQKIVDAAKKQNEELAKSISERDARIRKGELGYVERNNNTN

LNGNNASTDKEFGARKKALADELDAIMKARRERNKAVKEERDLTLSYESGVLALQAQL

KVLQEHRTINDVISNERKQLFQEEAKFAILEQRYADGSLTKSQAKLLSQKDIILEQAK

QKAELGDQIVLQERSNKLLDDNLKKTVQITNEANNVSLAANLSDREKQRAKELQALQS

NQLNKGGSVDDADFQALLEARRNFYAQEDALRENWLAGVQSSFANWAESATDAFSIAG

ELTTSVFTGITEQITSLVTTGTADFRSFTVEILKQIAKIATQLLIVKAIESSLSSFGG

TGGAIGSIGGALGFAGGGYTGDGGKYQPAGTVHKGEFVFTKEATSRIGVKNLYKLMRG

YANGGAVGGPSGYANGGAVGGTNVSVGGVTVNVNSGIGGGNDAKAIESGVKVIVAEEI
SKSFQQGGRAYQFLRGYN"
CDS 19631..19981
/codon_start=1
/transl_table=11
/product="minor tail protein"

/translation="MALQTFNWCLNVLNGGSSFTNANNIRVVSFGNGYQQRGTGGYRT

NKRTYTVSYVGREWEAVMNFCLDHIVKPFAWTTPQGELKMFVIQQDSISVSPSTKEVQ

```
                     TVSMQFTEVFTSMS"
     CDS             20021..20776
                     /codon_start=1
                     /transl_table=11
                     /product="gp38"

/translation="MASVKFENQLQSLFPGEVVTLIEVDGSKFGARVYRFHGEDISYT

PEEIMQAQTSGQPLPSKTITFRGEEYGARPFGISGINLTSDGKANKLTLILSNVDQAV

SALIRQYNGMVQAKVTIWVTIRDNINEDGSIADGDYRKMVYFIERPKQVDYKTASFEL

SSPIDMDGIYIPARLVQSVCYWASRGWYRSGNGCAYAGNRYFDKDNNPVDDPGLDFCA
                     GTVDACKLRFGENNQLDFGGCASASLQAKNNQN"
     CDS             complement(20741..20818)
                     /codon_start=1
                     /transl_table=11
                     /product="hypothetical protein"
                     /translation="MISSLTFGVSILLPLVLIILSLQTC"
     CDS             20787..21533
                     /codon_start=1
                     /transl_table=11
                     /product="minor tail protein"

/translation="MLTPKVKLEIMRHAKEDFPNECCGVVTQKGMAQKYHRITNASKD

TENEFILDAGEYMKISDEEQVVYIVHSHTGDGATTRPSPADICSCNECEIPYVIVSYP

EGDMRILEPEEMPLAGRPWGLGSFDCWGLIMSFHAKHGVKLTDYRVNYPWWNKEYDEN

IYDDNWIKEGFELVETDDIPAGSMIMMQIQSETTNHAGIYVGDNKFLHHLYGKMSKVD
                     TYSDYWRERTVRIVRHKDLPEGASYNAETD"
     CDS             21517..22083
                     /codon_start=1
                     /transl_table=11
                     /product="tail assembly protein"

/translation="MQRLIKVKLGLSLGRRYGKIHELCVESVPEALRALSVNIPAFKE

FMQSHVGQNTRFAIFADGKNLNEHRIKDFKAVKEIRIMPIPQGRKSGGLFQTVLGAAL

ITIGAFTGQVYLVAAGVSLAAGGVAQLLSPQATGLKSQESAQNRASYAFGSAVNTVAA
                     GYPVCLPYGYRSVGGSVFSAGSYAQDIA"
     CDS             complement(22096..22176)
                     /codon_start=1
                     /transl_table=11
                     /product="Glycerophosphocholine phosphodiesterase"
                     /translation="MSCAISHYPVLFLTIGAIVHQIKNPH"
     CDS             22163..25549
                     /codon_start=1
                     /transl_table=11
                     /product="tail fiber protein"

/translation="MAQDIKIYGSKGGSSKQKQPVEQPDNLISVNKVKVLLAVSDGEV
```

DNSFSLKDLYLADVPVQNQNGTYNYEGVTAEFRPGTQSQDYIAGLEGASSEVQVNREI

TNSTPYIIAVNNTQLSAIRVKLYWPRLINQESNGDLNGTTCEYAIDLSVDGSTYSEYT

RGVANGKTTSGYDRSIRVNLPAEFSSALVRVRKITADSESSTLVNGMQITTYQEVIDA

KFRYPLTALVYVEFSSDLFPNGIPAISIKKKWKLIRVPTNYNPETRTYSGTWNGTFKM

AYSNNPAWVLYDLVTNQRYGLDQRELGITIDKWGLYEAAQFCDQMVPDGKGGVEPRYL

CDVVIQQKVEAYQVIRDICSIFRGLTFYDGEQIGIVVDKPRQPSYVFTNDNVVDGLFN

RTFGSDKSLYTVANVQFDDVENSYQQDVEPVFELEATRRFGYNPVDLTAIGCTRRSEA

NRRGRWLLKTNLRSETISFTTGLEGMIPMIGEVIAVNDQAWSSNYTLNLSGRIESVSG

LQVFVPFKIDAAAGDRILINKPDGMPEYRTIASVSEDGMTLQLNTAFSFNPQPDTVFA

IDKENLALQQYVVTGIQKADSDGDDAFGYSITAVEYDPNKYDEIDYGVNIQDRPTSIV

EPDTLLAPENVQVSSYSKVVQGISVETMVITWDKVQYAKTYTVQWRKENGNWINVPRT

ANTEVDIEGIYAGIYQVRVRAVSDTENVSAWSEIVSASLTGKIGTPSAPTVITASDDE

VFGIMVKWGFPDNSTDTSYTELQQVADNGDGTYTPENASLLTLVPYPSYEYWHTTLPA

GKVIWYRARLIDKIGNVSPWTDFVMGMASDDVSAIIGEIKVDIENSEGFKYLQQNAIE

SNGAIQAQAESIIENTLANDGDVRRMTKENSKRKAEFIQAVNLIADEERARVEALTQL

KAQIDDEIVASLTRIDTALAEETQARTTADTALSARLGENEAALNEKLDTYVTADSIG

AQYGIKLGLTYNGVQYSSGMSMELTGSDGNVRSQVIFDANRFAISNGVSSGSGQWSLP

FVVENNQVFIQSAVIQDGSITNAKIGNRIQSNNYVAGSQGWAIDKAGSAEFSNATVRG
NLYANNGNFAFNGTNNTVQINNNGITVNLPNGGRVVVGVW"
CDS complement(25580..26542)
/codon_start=1
/transl_table=11
/product="hypothetical protein"

/translation="MPQGILIDLNDGRPAMEITAGLRAPAVTGSINTNGLSTPASSWD

FGLTMSPGSTAFCLPTKAVHVDAYDVVPEVYYINGFQKVNDGVGRITLSNFNGARGRI

IDFAGNCFEILDASSSSSPGILVENSTDFLAISSNSRLMSAAWVGGIQVNGAASLPVS

GIPFGKWDNPNVSLESDGSTIWCRDINYGGIDDVAASTYVQLVIFRNEPPIAGHGLTM

SNSSGQIVFSSVRRPFVLSGFIQITNGFQSINGGFFPLLRCGATTRVTGGYNNLRYKG
VCMSGGAVMAVPGTVIGNYSTQTGAQFPFDTNISMALPFIPNFY"
CDS 26397..26558
/codon_start=1

```
                     /transl_table=11
                     /product="hypothetical protein"

/translation="MVKPKSHDDAGVESPLVFIDPVTAGALSPAVISIAGLPSFRSIN
                     IPCGIISTP"
     CDS             complement(26520..26627)
                     /codon_start=1
                     /transl_table=11
                     /product="hypothetical protein"
                     /translation="MCATLVLRARLAAVTVVQLLTTDLRSGNNAARYID"
     CDS             complement(26542..26787)
                     /codon_start=1
                     /transl_table=11
                     /product="lipoprotein"

/translation="MTNLIKLSIIAAAAIMMVGCSSAPRPDGWCATQANGVCVAKWKN
                     GVVVPAGEVDVRYAGIESKVGGGYGGSVADHGSKEWK"
     CDS             26741..26896
                     /codon_start=1
                     /transl_table=11
                     /product="carbohydrate ABC transporter permease"

/translation="MIAAAAMIESLIKLVIYVFLMRFVSFRWSNYISFLRFVFSYSCC
                     LFYFLRH"
     CDS             complement(26856..27578)
                     /codon_start=1
                     /transl_table=11
                     /product="site-specific DNA-methyltransferase"

/translation="MEMIKLMNGDCLELMKEIPDNSIDMILCDLPYGTTRNKWDSVID

LDLLWTQYRRVCKGAIVLTAQTPFDKVLGCSNLEMLKHEWIWEKTHATGHLNAKRAPM

KAHENILVFYKKPYKYNPQMTEGHKRKTATKRNDNGTNYGDQNFESLSYDSTMRYPRD

VIKMASDKQKSSLHPTQKPVSLMEYLIKTYTDEGDLVLDNCMGSGTTGVACVNTNRSF
                     IGIELDTEYFNVAKNRINSTNS"
     CDS             27505..27660
                     /codon_start=1
                     /transl_table=11
                     /product="Uncharacterised protein"

/translation="MSIELSGISFISSRQSPFINLIISISHCVFVVMIIHNQARVSLA
                     IRATGYA"
     CDS             27889..27978
                     /codon_start=1
                     /transl_table=11
                     /product="hypothetical protein"
                     /translation="MHHPLKSVYTVYALFTLSYSGPAGAVKRN"
     CDS             complement(28028..28120)
                     /codon_start=1
                     /transl_table=11
                     /product="hypothetical protein"
                     /translation="MAFLYLSMVVCFRCDNYAERLSPRLIGIPD"
```

```
     CDS             28114..28308
                     /codon_start=1
                     /transl_table=11
                     /product="hypothetical protein"

/translation="MPYFIIKEKKNALFGDCYFGGEAGYTIHKSEADRFDTKEEAKSI
                     LTGYFTEEDLKAVKIVKIKD"
     CDS             complement(28194..28313)
                     /codon_start=1
                     /transl_table=11
                     /product="putative lipoprotein"

/translation="MVQSLILTILTAFKSSSVKYPVRIDLASSLVSNRSASDL"
     CDS             28312..29283
                     /codon_start=1
                     /transl_table=11
                     /product="exonuclease"

/translation="MTFTVYTNDELTNESYHEEQSHISGSSLAEILATSPAKWKFKQR

DNSSAALKFGTLSHCMMLEREAFDSTYMRATDFDEVEGLITSQTALSAALKKVGVSGT

SGKGYNELVEMMYKCGEDWPVKWLIEQQESAEALISGKELVSAKDYDKVIAMREVLTN

IPAYDAVINSPTAQKELSIFGEILGCGVKVRLDHVDVVDGAVYITDYKTTADASPRGF

GKSAYDHGYLCKMALQRDLFVKAFSEKRKVVVRLLAQEKTEPYLPMCYTLTDEQLHIG
                     RLQYMEALATYKQCKELDVWPGYSNGITEQDLMIPEWVAKQYGEILK"
     CDS             29247..29744
                     /codon_start=1
                     /transl_table=11
                     /product="DNA N-6-adenine methyltransferase"

/translation="MGSKTIRRDIKMNKDCWQTPLELFNQLNDKYNFQVDACASQENA

LCEHFWTEDDDCLQQDWGDENDFVWCNPPYSNPLPFVEKCQEYGYAVMLLNQDTSTKW

AKIVFEKSSKVILLSDRVRFINPSTLEIGKSNPKCQMIVVFDNSVEHGPVIRWCSIKD
                     IHKNI"
     CDS             29784..29963
                     /codon_start=1
                     /transl_table=11
                     /product="transcriptional regulator"

/translation="MITTSTTRWFKQMKVFKSKICSYIKMAINRNDDIIKTIDIKPGD
                     EVFVIIEKSIALELA"
     CDS             complement(29849..30019)
                     /codon_start=1
                     /transl_table=11
                     /product="hypothetical protein"

/translation="MILKCSYQSSSFSEFFKMYYASSNAIDFSIMTKTSSPGFISIVF
                     IISSFRLMAILM"
     CDS             30001..30651
```

```
                     /codon_start=1
                     /transl_table=11
                     /product="recombinase"

/translation="MNISESCKSILNALHTAKSLFAKAEKSKQNSHLKNKYATLEDVL

AAVEPGLDECGLVMFQSVIDDESTNRIKVETKLFHAESGEWVSFLMIVPISKQDAQGY

GSALTYARRYGITAALGLSQADDDGNLAAKGVKDFKRELEKCNTLDELRNVWKEAKQS

LDAAGWKVFEPHIIERKAEIQANEMNGFNPATPKKVAEKSSDKEKIEVESQQIDTF"
     CDS             30694..31131
                     /codon_start=1
                     /transl_table=11
                     /product="single-stranded DNA binding protein"

/translation="MHVITGQIRRAPFTKEGQNANGTWKMFAVDLSERYKDKDGQTQY

SNYRAVFFAKESILDWYNEAFQEGKIVSVSAESLSVDVRDKDGKTYVTINMNNPRLEF
                     SQRGDSQPQQNQRQQQQQQQNNQQKRQPQPQQQQQFDDSIPF"
     CDS             complement(31161..31691)
                     /codon_start=1
                     /transl_table=11
                     /product="hypothetical protein"

/translation="MDMPGPTPREWGQLAISYGARRCYIANSTYNGGLAVMELLHGLN

TTVDPSGFVKVASPIIKVFRDGSFETNEESEGASVERVSTGVYKVSGVLGLNSDTSWG

GVNGGFEVPVDLNKQPRIWLDYEVEADGSIVVKTYHRTHDSSPSFARNKIDGFSNGDP
                     IDIPSDSFVSIRVNMP"
     CDS             complement(31616..31795)
                     /codon_start=1
                     /transl_table=11
                     /product="hypothetical protein"

/translation="MDQGFFQRIGQQKHGLMDMSLTVPVLTYTKILLYSWICLAQLHA
                     SGVSLPYLMGQGVVI"
     CDS             31754..31834
                     /codon_start=1
                     /transl_table=11
                     /product="hypothetical protein"
                     /translation="MFLLSYSLKETLVHKLAGPALHRLHD"
     CDS             complement(31799..31894)
                     /codon_start=1
                     /transl_table=11
                     /product="hypothetical protein"
                     /translation="MTYKTSYKQGPILDLEPLRLLIMETMQGRSS"
     CDS             complement(31939..32070)
                     /codon_start=1
                     /transl_table=11
                     /product="hypothetical protein"

/translation="MMQSLGLRFLKKGGLKMKIKGEALEQLKKDCSLSISAASGRLR"
```

```
     CDS             31967..32092
                     /codon_start=1
                     /transl_table=11
                     /product="hypothetical protein"

/translation="MLREQSFFSCSSASPLIFIFKPPFFKNLSPRLCIIFCHEET"
     CDS             complement(32012..32182)
                     /codon_start=1
                     /transl_table=11
                     /product="hypothetical protein"

/translation="MVIMKEKTVSYSHNNGHWVVELKGFHKYPKLSFFMTEDDAKSWA
                     QVLKERWFKNED"
     CDS             32132..32296
                     /codon_start=1
                     /transl_table=11
                     /product="hypothetical protein"

/translation="MTIIVTVRNCFFFHYYHLSIFACYRNLFNVLATTRDFKPGPASW
                     QKDPQGALVG"
     CDS             complement(32241..32387)
                     /codon_start=1
                     /transl_table=11
                     /product="hypothetical protein"

/translation="MYSSKRCWTLSHFRKEPKRVNDTAQCEEALPNPPKRLAGPFARM
                     PGRV"
BASE COUNT     9543 a   6567 c   7830 g   8546 t
ORIGIN
        1 tatagcaata ttgcatcgaa tgtaatggtg tttttgattg ttgcagaatt
tgcaattatt
       61 gatgtgttag tgaatgaatg ttggaagaac taccatacaa gttgattttg
attctggctg
      121 agccatttta cggtatacgt gtaacttctg tatattcatt gtagtcacaa
aagtaagtga
      181 ccactaacat acgtttttac gcgaaatata cacgccatat acaatataat
atctatatat
      241 atcaaccttt tactatatta ttattattat tattatatat atgtatactg
tgtataccgc
      301 tcatagatta aatagttgat atattcctct gtgatattct gacacttgtc
agaattggat
      361 gacgcttgtc agaataaaaa ggcacatata taggaaatca catcaaaaaa
ctgtacacag
      421 cggatacggc aaggttacat ccagtaatgt cgtggctttg gagactgaaa
agtgtatcaa
      481 tgataaaata tactaaaata tacattgacc gacacgtaaa tctctggcac
tgtggtaatc
      541 actgaaacgc agtacacaac aacgaggcat tgaaatgcag aaagtaacac
ttggtgaagt
      601 catccgtgag atggtgcgaa aggcaatcga atcggaaagt ggtgagttta
aggttccggt
      661 cagccagata ttcaaaatga ttcgtgggaa accgtacccg gaaatggagt
atgacggcga
```

721 gaccgatgag atattgaatc tggcagacag gtcattaccg gagcttaaga
actcatatat
781 ctacaatact gtatcgcgaa tgacagagtt acgagacgca aataagaggg
cgcgatacaa
841 gtttatctgg attgatgacg aaggggagca gacaagcgca aggcaatttg
atggcgacgg
901 cgctgacaag cacctggtta tatatattga gaacggtgcg cactggactg
gtaatcgaga
961 gaagaagaaa caggaagctg agaaggaagc ggctaccatt gagaaattca
agaaacgact
1021 tttggcagtc actccgagca ttatccacct tagcggcgaa aagcttgaag
gtgctctgtt
1081 cgcgctatca gcataccagg aaatgattaa ggaagccaaa tagcatgtat
ggcagtagac
1141 taccatacca cttgcggtac ggtgaggaag aggttgaggt ggcgatggag
ttctatcgga
1201 aagctaaata actcctgagt ggaaaatagc actttttgcc taacgctata
acgccatgga
1261 tggcataatc accacatcaa cagcgagatg gagtacaaag ataaaaatgg
cacaccaaat
1321 tgaagttatg agtgaaatta atgacctatt acttacattg gttaagatta
ttgacagacc
1381 agtagtgagt tacttagttg gttttgaaga gatatttatc gagttttaca
agcaaccaaa
1441 tagcccttgc tttggtgtag ttagtgagac ttacagattc aaaagttgca
gcgaactaag
1501 agattgcgtc aacagtttaa ccagtagcac cactgattgc taaacctgcc
gcaaggccat
1561 ttgatatagt ggccttattg aagcaaagca aaagagggtg tgatggaaag
gaacaaatac
1621 gaaagagaga ttatcggcat taatggcgag agggctattg ttgatgtgta
tcgcacgcta
1681 tcagcattca gtgttacaga cccagcatgt cagcatgcta tcaaaaagct
gctctgcatg
1741 ggtttgcgtg gccacaagga tgtcattact gaccttgatg acgcgataga
ctcgctaaat
1801 aagatgaaaa cttacctgga gcaaacgaaa tgattacaat taacctgtca
gacaagcaag
1861 ccgcaactct taagcagcta ctatcaagcc agccaatgcg gtgctcagcg
gtcgagttct
1921 cagggatggt caaagatgtt tgcactcaaa tcagcaacca gcagttcgat
gtgtcagact
1981 ttgagtcatc aaggcagtat gagtcactgt caatcgagag tgttggcctc
acccaaaaaa
2041 ccgagctata taaaagtata gggtggatta taaaggaaag ctctcagaat
gaaggatacc
2101 taactaactg ggatgatgag actaataaat gatttacatt cacacttatt
acacaggtaa
2161 gtttaatagc gttaagcatg ttcgggttta tgaggacatt gatgaggttt
tgggccagtg
2221 tcgtgttctg ggaggtgaca ttaagatttg caggttgatt caagtgattt
tataaatagc
2281 acgaattgct aaacaatcgc cggagcaatc cggcatgatt aacacatcga
caacgagatg

2341 gagtagagag atgaaggtca agattattga agttggcatt gctgtagatg acgagctaag
2401 ccttgcggat tgcggtttcc aggttggtga tgtggtggag gtttccggtc agtacaaaga
2461 tggaaactta tcagttaagg cgattcgtga aacaaatttc gtatcaatcg gaaatgaagt
2521 cagcatccag gaaggcgaat acgaggtgat tgaggaatga aattcaagca aagtaaccct
2581 aagctgcaag ccaaccactt cgatggctac tcaacattca ttattgcaca ggcttgcgga
2641 atgcgtgatg aacctcaggt tcacaccatc cacttaaccg aggagcaggc tagagaactc
2701 gcttatgaaa ttcttgagca attgaataag atttaaggtg atagaatgaa attaacaaag
2761 ttacaaagag atgaactaaa gtttaagcta ggtggattct gcgcatactg cggctgcgaa
2821 ctcggtgata agtggcatgc tgaccatatg aagccagtaa tcaggtatga tggtaaaatg
2881 gtgcacgatg agcttgataa tatttcgaac cttgtcccgg cgtgccacgc ttgcaatctg
2941 cataagcact gcaatagtgt ggaggattac cggagaatta ttgacgatgg taggcgggag
3001 tttttggcat ccggcaaggg taaggcgctc gttcgcatgg ggctggttga tatgaagatg
3061 gatgatattg tgttctggtt cgagaagttt gagaaattga ataagattta acgcgtatag
3121 aacgcgttct aactaacaat aatttaaatg aggtgattgc ataatcaagg tcgtatacat
3181 cggtgaggat gattatatgg cgcttttaaa gcacggcgac gtcttctgtt catggactgt
3241 tagtagtgat ggtaactatt acattattca aaataagaat ggagaggaaa tttacctctc
3301 gaaagatgag gtagcacttt actaatggat aactacgaat caccaagcca gtgggctaag
3361 cgcatgatgg atgaggctaa gactggcgaa gagtcaatgc aatacttcga gctaatgaat
3421 ctatggaaag agcgggagga taagaaaaat gcttaacacg tgctttttcc gaactatcag
3481 gagtttgttg aggatttaat ttactttcac gataaatacg gcgacacgtt tgagtgcgac
3541 ggtgataaca acgaagtttt agcaactttt gatagaggga tcgatgtaaa tagcacaaat
3601 tgctaaacaa gagtggcaag gatgtcatat aattaattca tcaaacaacg agatggtaga
3661 aaatgttcgg attaaacgag gcgcatttca atgcggtcaa gaagcaggct aaaaagctta
3721 acgatgatta ctctaagtta tcaccaaaag agcgaaagaa tgacaagcta gttgctgcgc
3781 taatatcagg tttatgggag ccagtattta ctattatcag tcgagaccgc ttcgtttggg
3841 tggctggata tttgaagggt cgcgttggcc atgatgagaa tgggaatagc ctttatgaat
3901 aagattaagt cattaatgtt taaggttgga atcctacata agcataaata tagattattt

3961 cgacgcataa attggtatga ggacgaatat caggatgggt gcaggacaaa actaatctgt
4021 acagtttgcg ggaagatttt taactcaaag gttgacctaa tggtttgcaa aagcacgctg
4081 gatttcatta gatatgaaaa agagattgca tcacttaggg gttttaatgg ataagtttat
4141 tgaatgcgta gtatacacct tatgcatcac tctcgttttt tgctcattga tgattagctt
4201 tggtgtgctg tttagaatca tttaggacaa aatccttgca tcactgaccg gaaagtatta
4261 gtatgtacct agtagcacaa aaggccacta agtggcaacg gcgcacggag cgaaacacaa
4321 agtccatagc tcactaagca gcgtacatgg caaaatcggt cgagaaactc gacgctggat
4381 gcgtaaccag caatctttca atagcgaaat cgctacttaa agatttgttc tgctggcgta
4441 acaggataac gcaaagacct cctaagtctt agttactggt tcgagtccag tgcggaacac
4501 caattaagga gattcattat gtgggacgtt aagctttcaa tccgcttaat gggtcgaagc
4561 tgtaagaatt gccagcaaga atttatcgcg tcggttgccg ctgagtcctc agaggaagcg
4621 gtagcgaaag ttaaggtaat gtcgggagcc gaccctgatt accacaaatt cctagtagct
4681 tacgttcgtg agcgaaagta aaagaagttc tactctctgg ttgtttcatc tctctcgttg
4741 ttgttcgaca cttcgctctc ttcttgaggg ctttttagt gtttgtgttg acaataatca
4801 acgggtgcat tagcattaac tcatctgggc gacccactaa ccagaaggag atttaaaatg
4861 atgacattaa aagcagtata caaagacggc agcgagcaga ttattgaagt taaaagagtt
4921 ggctacgtgc aaggttctcg acttgttgac ttcgttgatt gcgatggtcc aggtcagttc
4981 ttcatggagg accgagtatt gtcagtttat gtgatgaatt cagccggcag caccgttggg
5041 aaataccagc tagcggattg agttgctatc aagcccctta attggggctt ttttacgtct
5101 gcaatatgct ataatgctaa cctgaacaaa aggaggacga aaatgaaaga cgaaaacttc
5161 agcagcacag cacaagaaaa ggttagctct ggaaacttta aagccatgta caacaaaaag
5221 tattctgaca ttacaacgct agccaagaac cgaattatga caccagagca gctatttgac
5281 attgctgtga aatacttctc ttgggcagaa gaccaggcaa tcaaggcaat tgaaaccgct
5341 agcttccagg gtgttgtcac tgaaaacctg gtgcacaagc ctagagtatt cacgctaact
5401 ggattctgcc tttactgcgg cgtcacccat agcgccatcg aaaaatggag gaagtctgct
5461 ggttatgacg aggtaattgc gttcattgat tccgttattc gtgagcagaa ataccagcta
5521 gcagcatctg gcattgttaa cccaggcatg atttccaaag aacttggcat cgacaagcca

5581 caggagatta acatcaccag cacatcttca gctaatgacg tcgacacaat gaaagaagcg
5641 ctggagtcgg taattagtaa actgtaagaa tggagagagt agaatgtcta atttagtttg
5701 ggaggaaatg acgtcagccg aaaagatggc ggttaaggca atatcaactc atagctttga
5761 gggttttctg agggtgtggt tccaactgac acagggcgag cggtacatcc caaactggca
5821 tcataaatac ctttgcagga taattgacga gattatcgct ggtgagcgta aggacaccat
5881 cattaacgtc gctcccggta gtggaaagac agaaattgcc tcaatccact ttccagcata
5941 ctcgatggtt aagctgaaga aggtcaggaa ccttaacata tcgtttgccg actcactggt
6001 taagcgtaac tccaagcgag tgcgtgactt aattaagtcg gttgagttcc aggagttatt
6061 cccatgcaag tttggcacat gcaaggatga tgagctacag gttctggatg agtctggcaa
6121 agtacggttt gagtcaatct ccaaggctgc tggaggccaa atcaccggcg cgaggggcgg
6181 ctatataacc gattcatata gtggcgcggt tctgttagat gattttgaca aaccagccga
6241 tatgctttct gccgtgttcc gcgcaaacaa tcacgtgatg ctaaagaaca ccatccgctc
6301 tcgtagggct agctcagtta aaggtaaagc aactccgatt atctctattc agcagcgact
6361 tcacgtcaac gatagcacgt ggttcatgat gaatggaggt atgggtatca gcttcgattt
6421 gattaagata ccagcgctag taactgaaga ttacgtggat acacttcctg actggattaa
6481 gcagcaattc attgatgacg ttctatcaag cgaatatatt gagcgtgacg gcgtgaaata
6541 ttacagctac ttcccagaaa aggagtcggt taacgacctg gtagcaatgt gggattcaga
6601 cagctataca ttccttagcc agtatcagca ggagcctgta gcgcttggtg gtaatctgat
6661 taatgtagaa tggctccagc gcattagcga cactctgcga ccgccagcta agtatgatta
6721 cagattcatg actcttgata ccgccatgac aacaaagtca tacagcgact ttagtgtact
6781 gcaactttgg ggttataagg atgggaagat ttacctgctt gaccagcgac gaggtaagtt
6841 tgaagcgccg gagctagagg ctgagttatt ggatttcgag aagatagcaa gggcatccaa
6901 tcagacagat ggtatccttc gtaaaatcat catcgagaag aaagcctctg gcatcgggct
6961 gattcaatcg gtaggtaggg ttatgcggac accaatcgag ccatatgtgc cagataacga
7021 caagctaact cgcgtaatga gtgcactgcc tcagattaag gctggtaacg ttatcctgcc
7081 tgagtctgca acatggctaa acggattact aacggagatt gcggcattca cggcagatga
7141 ctcgcatgtt catgatgacc agattgactg tttaactatg gctgtcaacc ttgttctaaa

7201 cattgcagag gatcctaaat caaggatgat gagattagca ggaatcaaat agcacttttt
7261 gctaaagaca ctccgccatt ggtggcgtat agttacctca cgcaaacaag aggagaaagt
7321 acaagtggca accaaattaa aagctgtata tcttggcaac gaagatggag agtatcgtgg
7381 tttcaccgta ggaaatgaat atgaggttgg taattacata gctgaaaatg actgcttcgg
7441 ttcttttgat gatgatggtt gttatcttta catcaataac ggagcattcc acaagtttcg
7501 aatccacgat gagaatggct acctcaattt aaaaacacaa gatgaattta tcagcaaaga
7561 aaagcagtta aaatactaca tcaatggcga cgctgtcaca aaatgtaagt tcaatgacgt
7621 gctgctatgc gtaatgggat ttcagtctga tgggattgac gcatcatcaa tcaagttcga
7681 agtaaaattc gagtaaccat aaaagctatg ttaaactaag cccctaacgg ggcttttta
7741 ttggagcaag aaaatggtaa aagcagattc atatgccacg cagttcctcg gtggggctag
7801 tgatggcagc gagatttacg gtcactttgg caacacctac ctaacacctc ctttcgctga
7861 ttcactttac gtatccaact caattgttcg tcgcattgtc gacaccatac cggagactgc
7921 aatcgcagca ggtttccata tcgacggcat tgatgacgaa ggtgagttct ggtccaggtg
7981 ggattacctg aaggttaacg agtcaatatc tgacgcgtgg tcatgggcga gattatttgg
8041 cggctcggca attgtggcta tcgttaagga taatcgtgcg ttaactagtc cagtacgtga
8101 aggcgcagaa cttgaatcac ttcgagtata cgagcgaagt caggtaaagg tgcaaacgaa
8161 ggaggaaaac cctcgcaacg tgcgatttgg taagccgcta acgtaccgca tcacgcctga
8221 aggcagcacc atgttttatg acgttcacta ttccagggtt cacatcatgg atggtgagaa
8281 aatccccagt tcattgcgtc gcggtaatga tgggtggggt gctagcgtac tgactcctga
8341 cttgctggaa tcaatcaagg attatcagac gtgcgagaag ctggcaactc agttattgcg
8401 acgtaagcaa caggcggttt ggaaggctaa aggtctggct gaattgtgtg acgacgctga
8461 aggttttggc gcggccaggt tgagactggc tcaggttgat gataactctg gagttgggcg
8521 tgctattggt atcgatgctg aggctgaaga atacagcgtt ctgaattcgg atattggtgg
8581 tattgatgga ttcctcgata agaagttcga caggattgtt gctctgagcg gtattcatga
8641 aatcatacta aagggccgta acgttggtgg tctatcctcc agccaaaaca cagctctgga
8701 gacatttcat aagctaatcg accgcaagcg caatgaggag ttactacctg tccttgggtt
8761 cttaatccca ttcatcacaa cggagcaaga atggtcggtt gagtttaatc cgctagctca

8821 ggaatcgaag aaagaccgcg ctgaaatact gaagaataac gttgactcaa tctcagccct
8881 tatcactaac ggcgtaatgg atacagatga agcacgagat accttgcgtg cattagcacc
8941 tgaggttaag attggtgaag gttcaattgt taccgagcaa tccgctcagg aagatttacc
9001 aaaagaggag gttgtaattg aataagaatc gctttgatac agcatcattc aaagccactg
9061 tcgacgagaa tggattcctc gtcgacactc cagtggtggc aaggcttggc gtgcaggttt
9121 attacatgga ggatggttct acagttcgcg agttcaggcc agcagaggaa gtattcaaag
9181 aggaatcgct agccagttat cagggaaagc caatgactct gaaccacgta tttgtaaact
9241 ctgataacgc caaagatgta gtggttggtt cggtaacagg caaagccgaa gctcttggca
9301 gtacagttat tgcgccaatt gtcgtttact ctcaacaggc aatagatgaa gctcgcactg
9361 gtaatgctaa ggaattatca gttgggtact cagcagttct tgacgaaact ccaggctggg
9421 gcgatccggc aacaggtgag tatatcctca ggagtgacgc gaaggaaagt gatgtacccc
9481 ctgactggca agaattcgat gcaatccagc gagacattaa ggtcaaccat ctggcaatgg
9541 tatctagagg ccgtgcaggt gtggcgaaat tgaatatgga cggccagcag gaaaaccctt
9601 atactgacaa cgttgattct attaaagagg atgttaaaga aatggttaag attaaactga
9661 atggtactca ggagttcgaa attgctccag aagttgccgc acacattgca actattaaaa
9721 aagacgcaga tgatgctaaa gctaaagccg acaccattga agctgagcgt gattcgctga
9781 aagctaaagt tgatgcgatt cctgctgaaa ttgagaaagc cgttaaagcc gctaaagctg
9841 acgccgaagc acttgctgct ctggttactg tagccgcaga ggttggcgtt aagactgact
9901 gtctggatgc taaaggcatt aaagttgcat acgttaaaga agtttctggc cttgatgttg
9961 ccgataaatc cgacgcatac atcgatgcag cgttcgacat tgccaaagat tctgataaaa
10021 tggctgaagt tcgcaaagct accgcagctt ccgacaaatc tgacaagtct gacgaaccta
10081 agaaattaga cccgcgcgct cgtctggcta aaattaaaaa gtaaggagat agataatggc
10141 aattcgttct gtagcactgg caggtatggt agctgatacc tccctgtata atatcgatgg
10201 cgcttgtgta gttggtggct ctgctgcaat tcctgttggt actgttgttg gtgttacgtc
10261 cgctcagcct gttgatgggc acaaggtagt agctgcaact ggtattaccg ccgctaacgt
10321 tcttggtgtt gttgttcgat ctcattacga gactccagac ggcacagctc gcgtaaatga
10381 agcggttaac gtaatgactt ctggtcgtat ctgggttcgc acaactcttg atgccgcccc

10441 tgcatttggc gctccggttc ttgtttctgc aactggtgtt gttgaggagg ctggtgctgt
10501 agcgactggc tggaccttcg ctggtggctt catccctaac actggcacta agccgcaaga
10561 ccttagcgta gatggcgctc tggttgaagt tcaggttaaa cagaagtaat aaatatagca
10621 gtgtaaataa gcctccttcg ggaggttttt ttgtaatcaa atgctgccta tgttatattc
10681 aactctcatc taaaaaaaat gagattaaac atggcttaca ccgaagttaa ggctattagt
10741 gcagatagcg gtcgattcaa tacactgctt caagccgcca tcgatggtgg ttttcagcca
10801 gtaagtaaaa tcacggtaaa tggctcacag tcatattcca tcgtcgtagc aaaaggcgct
10861 gacagttcaa tcactgaagc gaatgatcac ctccaccaat atgcgcaagc tgttcgaagc
10921 tgttgaagct gcgtctgatg atggcttcac aattgatacg tcaagcctga gtctatcatc
10981 aggtcagttc tttgcttatg catataaagg cagtgctggt ggttctgttt ctgtggcgtg
11041 gggagacgta actggcaaac caaccacctt tgcgccaatt gtcggcactg gtgcgaccca
11101 agcaatggct ggcaataaga cgctggctaa cattggtggc gtagttccct tgtctggcct
11161 tcctattgca agcaccactg ccgctggcat tatccagatt ggcactggcg caactaacgc
11221 aatgccgggt aacaagtttg ttgctggcgc ggcggttgct aacgttactg ctcagaccgt
11281 gactggtgct gatgccgaag cggtggcaac ctcagctacc acagctgtta acgaagtagc
11341 caccaagtta aacgaccttc tcgcacagct gcgagtcgcc aagatcatcg cgggctaacg
11401 ccaagctggc ggataactca cctgttgatt tgtggtcgcc atgctgtata attgactcgt
11461 acatacataa ctgaaaagga ataaacaaga atggccatta aatttgatgc agaacaagca
11521 aaaatcaccg ctcaccttga gcaaatgggt gtagataagg ctgatgctgc tggtatctgg
11581 accgttaagc agttgaccgc gaatctggcg cgcgcttacg aagcagagta ttcggaaaac
11641 tccgtagtta atatcttccc tgtaactaac gaaatcccaa gtcacgcaaa gtattttgag
11701 tatcctgagt ttgacggagc agcaattgca cagattatcg ctgaatactc tgatgacctg
11761 ccgctggttg atgcgttcat gaccgagaaa caaggtaaag tgttccgctt cggtaacgca
11821 ttcctgatct ccatcgacga aatcaaagca ggtgcagcaa ctggtcagtc actgtcatct
11881 cgcaaacagg ctctggcatt cgaagcgcac gataacctgc ttgataaact tgtatggtct
11941 ggctctgcac cgcacggcat tcctagcgta ttcaatgcgc cgaacgttaa ccaggttgtt
12001 gctggtactt ggaactccgc ggctgctgct atgactgaca ttaccgctct tatcgatgct

12061 atcgaaacag cgaccaatgg cgctcacacc gcgactgata tcctgcttcc agcttccgct
12121 cgccgcctga tgcaagaatt ggtgccgaat acctctatca gctacgcgca gttgtttgca
12181 accaataact ccggcatcac tttgcgctat ctgcaattcc tggacaacta cgacggcgca
12241 aacggcaaag ctgcactggc atttgaaaaa aatccgttaa atcttagtat tgagattcca
12301 gaagttacta acgtacttcc gggccaaccg aaggatttac atttcaaata cccggtgact
12361 tcaaaggcta caggcttgat tttatatagg ccgctaactg tttccgtact gaaaggcatt
12421 acctacgcct aatggcacta aatgacaaaa caataccacc gaaaggtggt atttttgttt
12481 taacacaacg ctaatagtgg tgcggttatg gaattttcag attatatagt ttacgacaat
12541 gggaagttac gctggaagaa gaagacaggg aggtcaaccc agataggcaa ggagatagga
12601 ataaattcag ggagcggata ccttacattc aagttttatg gtaaaagata caaagtccac
12661 agggttgtgt ggatgataat ggttgggcca atacctgatg gtatggaaac tgatcacata
12721 aatcatgttc gagacgataa tagaattgag aatctcaggc ttgtatcaag gattgagaac
12781 atgaagaaca aaagcgttta cagaagcaac tcttccggat tttgcggggt tagtgtcaaa
12841 agcagcggca tgtaccacgc ctacatacag tttaatggga agcaagtaaa ccttggttta
12901 tttaaggaaa agcaggatgc agtaacggca agattggagg cagaaatggt ttatggattt
12961 catgataatc atgggttata atcaaccatc aattaataaa agtggagcaa caaatgattc
13021 gtttagaaca cactggcgcg tgcatgatta cctttcgtgg tgtgaattat atgccaggtg
13081 aattcatcga ggtcgaggaa attaacgctg gacttaagag cttgattgca gaaggccgct
13141 tgcagattga tggcgacacc aaagcaacta aagaaattgc cgcagaggtt aaagcaaagt
13201 ctaagcgcaa agaaccgaag accattgacg aagctcagac tggtcaggaa tataaataac
13261 caaatgggcg cttcggcgtc cgtttttgca tgggggtttt atgaacgaac aagcattggc
13321 atttatgaga tcactggttc cagctttgcg taacgttcca gatgaaacgc tggacgcatg
13381 gcttgaactg gctaaacttt acatctgcgc tagtaagttt ggtgacgatg cttacaaggc
13441 gctggcgtta tacactctgc acattgcctt tcttgatggc gcgcttaagc agaatgggtc
13501 gctcgatgat tatgggaaga aaattgcaag ttactccctg agcggtgaat actctatcag
13561 gcatgaatcc acctcgcaaa cacaatcatc aatgacagcc accccgtggg gtaggcttta
13621 ctggaacctc ctgcgtaaga aaggtggcgg ttttggcttt attacgtccg ctgggcgtgg

13681 gtgcggttgc cgatgaatta ctcccaagta gaatcattaa tgcgcacggg
catcaatatc
13741 tttagtgatg gcgatggtac gtttaccatg gaaactggta aaggcggtgt
tgaaattgtt
13801 gacggcgttg aagttcccgt ggctggcgga actgcgttaa ttaaggggct
ggtgagggag
13861 attaaaacca gggatattga tggtgagtat attcaatttg gtgacaagcg
tggaattttc
13921 acttcggaag tcccaatctt gcagggctac aggattatag tggacgatga
aacttacact
13981 gtagtcgacc caaggccagt taaaccaact ggcacagtgg tcgcttacag
gccaattcta
14041 agaaggattg ctacctatgg ctagaaacat tggtggggta gctacattta
cgaatagtat
14101 tgctgattgg attgataggg ctgattctaa tgtgtctgaa gttgtgtctg
gcactgttat
14161 taaagcagct aacgccattg ttgatttatc tcccgttgat actggtaggt
tcaaagccaa
14221 ctggcagatt accgctaact caccagctgc gcagtccctg aatgagtacg
ataaaactgg
14281 tggcgatacg aaaagatatc tggcaaggca agctcaagca gttgctaact
caccagcaac
14341 aaaggttatc tacataacca acaggcttga ttacgcttct gaccttgaat
acggtgcatc
14401 gcagcaagct ccggctggtg ttcttggggt tgtaaatatc aggcttggac
ggtacttcga
14461 agaagctgtt gctgaaacaa ggagtaaacg ataatgcagt atgagctatc
tttagcagca
14521 aggaggtttg tgactgattt ggttaaaacg tttccagtca ggtacccaat
ctcatacgaa
14581 aacgtggcct tctctccacc aagcaatgga ggtatgtggc tgaaatacga
ttacacagag
14641 gccgatacgg taacttatag cttgagtcga aagtgcaaat actatgttgg
catggttcag
14701 gtgtctgtat ttttctcacc aggtgatggt gttgatagtg caaggagaat
tgctaatcaa
14761 ttggcagaat caatgcttga tggtacaatg cttgacacag gctacattta
tgagggtggg
14821 gtagttcacc cagtagtgaa gtcaaaatct ggctggttta ttccagttcg
tttctatgtt
14881 cgtcttgact aaaaaggaaa atatatggcc catctcagca atggcaccca
agtcttcgta
14941 gaaggtagta ggggtgaagc tatcaacgta accgcaattt ctaatgcggc
taaccctgtt
15001 tttaccgtgg ctgccagttc aggcttagcg gttggtgact acattctagt
aaccagcgca
15061 tcatctacat ttatggcaga taagcagctt cgcgtaacgg cagtttcaga
cactagcgtc
15121 acggttgaag gcattgacac ctccaatgtc accaaattcc cagctggcgg
aacggctgaa
15181 atcgttaaga ttcttagttg gtttgagatt ccttgcgttc aggacgtatc
aactgacggt
15241 ggcgaacagc agttcgttaa cttccagtgc ttgtccgatg accgtgagca
acaaattcca

15301 acttacaagt ctgcggtaac aaatacctac acgttcgcgc acgaatacac
caacccggta
15361 tatcctattc tgcgtaacta tgacgaagct ggtactgttg ttgctatccg
tatgtttgta
15421 cctcgcgcta gtgagatgcg tcttcagtct ggtaccatcg cctttaacga
caccccaagc
15481 attggtgtca acgaaatcga aacggtatcc atcgcggtat ctatccgtgg
caggctgagt
15541 tctgtagcat ctgaactgtc ataaaaatta accctcttcg gagggttttt
tattgatagc
15601 actttttgtt aaaggctatt gtgttgattg ctgtatagtt cattcatcga
aacgaaatga
15661 tagattaatg aaatgaacaa cgtaaaaaat atcatcaaac ttgctgctgc
tcttggtgct
15721 ttctacctgg caattcagat tgtcaaccca actagtggcg caacagcaaa
ctctgatgca
15781 ataaagtatg aaactaaatt caatagcgca tattcatact gtgaaaaagt
tgctggagac
15841 aaggttaatc agatcatgac tcatggtcgc gaagtgtgca cagaagttgc
tcacaaaatt
15901 gcaaaagaag acgaatcaaa gtaatataat cattcaacac aaacaaagga
gataatgaga
15961 tggcaaaatt taaactttca cttaaggcgc ttcctgactt taaacttcca
gttaagtttc
16021 agctggctaa tggtgaagat gctgaagtag tactaacagt taagcacaag
aaatcatctg
16081 agttgcagga gattcttaat cgtgaaaact tcagcactaa agaattgatt
agcgaaatct
16141 gctctgactg ggatttagaa gagccattta acgatgaaaa cgtagatgag
tttgcaaacc
16201 tttttcctgg cgctgttcca tcactgatgg atagctacat gaaagcgctt
gcaggtcagc
16261 gagtaaaaaa ctaaaacgcg cagtatacct acaataccag tcgccgccaa
cggattccga
16321 acttgaagct ataggaatga aacgttccga ctacgaaggt gaagagcccg
aggagttatt
16381 cttcgatgaa acaatgatgc aaagctggga tgtgtactgt gctatgtcaa
cacaatggcg
16441 agtaggagct tgcggcgcca caggtcttga ttacaattgc cttccattct
tgttcaaagt
16501 ctataaaata gatgatgaag aaatggcact taacgatatc agaatacttg
aggctaaagc
16561 cttagatatg atgaacgcca agaaatagcc atccttcggg gtggcttttt
ttattatgga
16621 gacaatatgg caactaacga aattgctggt atcacgctag ccgtcgacgt
cgctcaggtc
16681 gatagaggta cgcagtcctt acagaaattc aggcaagcaa acgagcaagc
tgcaacaggc
16741 attaatagct tcgttaatgc tgaacttgta gccaagactc aagcaaggga
tacagcaagg
16801 cagcttgagg agcagcgaaa atctttcgca tcgcttcagc aagtaatcga
cccaactgct
16861 gctaaattcc agaagttgca gaaagcggca accgaattgg acaaagcatt
tgagtctggg

16921 ctggttcctg atgatgagtt cttcaggctt ggctctgtta ttgaaactca aattaataag
16981 ctaaacaggt ccagggctgc gttgagtgaa gagggtaggg ctgcactcca agcttcaata
17041 gataaagaaa aagcaacagc aaccgcgcaa aagttcattc aatcccttga gaacgaggcg
17101 aatgcggcaa ctctcacaag gactgagctg cttcaactta aggctgctca gctaggcgtt
17161 agcgaccaag cggcaccgat tatcgacagg attggtaagg ccgctgaggc gtcagcagtt
17221 caattgcaga agcaaagctc agcttttgtt aagtcaggtt taagcgcagg gcagtacaag
17281 caagcaatac gacagctccc tgctcagatt acagatatcg gaacctcact tgctggcggc
17341 atcccaatct ggctgattgc aattcagcaa ggcggtcaga ttaaggatag tttcggcggt
17401 attgcgaaca cggcaaggtt tgcacttgcg gccattaatc cattgacagc tgttgttggt
17461 actttggggc tggctcttgg tgctcttggt gttgcagctt atcaggctga taagaacact
17521 cgcggactag catcaacgat aacgcttgtt ggcaattcaa ctatcacttc aacggcacag
17581 ataaatcagg ttgttgacgg gattgagaaa acaacattag ccacgcgcgg tcttattcag
17641 gaaatagcaa atagtctggt agcaaacggt aatcttacgc ttacgcagat agataagata
17701 accaaggcaa cagcgcagtg ggcgactgta acatcaaccg actcaaagac tatccttgga
17761 tactttgacc agatcacaaa ggacccaata aagggactta ctgaccttga taagcaattt
17821 aactttcttc agtatgggca gttaaaatat attgagaata tcaggaagac ggctggagaa
17881 acagaggcag taaccgcagc cactgaacta ttcgctgaag ttatggaaaa taggctagca
17941 gacgtagcgc taagcctcaa ccctcttgaa agcgcgtgga atagcttcag gaagtttgta
18001 agtgagatct tggatgacat cagcaaaagg acgcttgggg cccttaactt aataactgac
18061 gttgttgctg gtacgattga gcagataagg tcactcataa actccggcga cattctcttg
18121 ggtgagtttg ctatagcagc aaccaagacg cttcagtcaa tacccggcct tggtgatata
18181 ggtaatgagg caattgctga gcagcaaaag attgttgatg cagccaagaa gcaaaatgag
18241 gagttagcaa aaagcatatc tgagcgagat gctagaatca ggaagggtga gcttggctat
18301 gtggagagaa ataataacac taacctgaat ggaaataatg catcaactga taaggagttc
18361 ggagcaagga agaaggccct agcagacgag cttgatgcca taatgaaggc tagaagggag
18421 aggaacaaag ctgtaaaaga agaaagggat ttaaccttat cttatgaatc tggcgttctt
18481 gcacttcagg ctcaattgaa ggttttgcaa gagcatagaa cgataaacga tgtaataagc

18541 aacgagcgca agcagctatt tcaggaagag gctaagtttg caatactcga gcaaagatat
18601 gctgatggta gcctaaccaa gtctcaagcc aaactgctct cgcagaagga tatcattctt
18661 gaacaggcta aacagaaggc tgaactagga gatcagattg ttcttcaaga gaggtcaaac
18721 aagttacttg atgataacct aaagaagact gttcagatta caaatgaggc aaataatgtt
18781 tctcttgctg ctaatttatc tgacagggag aagcaaaggg cgaaagagct tcaggcgctt
18841 caatccaatc agttaaacaa aggtggctct gtagatgacg ctgatttcca ggctttactg
18901 gaagcaaggc gtaattttta tgctcaagag gacgcgctac gtgaaaactg gctagccggt
18961 gtgcagtcat cattcgctaa ttgggcagag agcgcgacag atgcattttc tatagctggt
19021 gaattgacca cttccgtgtt taccggaatt acagagcaga ttaccagcct tgtaacaact
19081 ggcacggcag acttcaggag ctttaccgtt gaaatcctga aacagattgc caagattgca
19141 acacagctct taattgttaa ggcgattgag tcaagtttaa gcagcttcgg cggaactggc
19201 ggcgctattg gttcaattgg tggcgcgctt ggctttgctg gcggtggata tactggtgat
19261 ggaggtaagt accaacctgc cggaactgtg cacaaaggtg aatttgtttt caccaaagaa
19321 gcaaccagca ggattggagt caagaacctg tacaagctta tgcgaggtta tgcaaatggc
19381 ggtgctgttg gtgggccatc tggttatgct aatggtggtg ctgtcggcgg tactaatgtc
19441 agtgttggcg gcgttactgt taacgtaaat tctggaatcg gtggcggcaa tgatgctaag
19501 gctatagaat ctggagttaa ggttatcgtg gctgaggaaa tcagtaagtc attccagcag
19561 ggcggcaggg cgtatcagtt cttgcgcggt tacaactaat taatggggct tctgcccta
19621 aggatttaaa atggcattgc aaacattcaa ttggtgctta aacgtactca atggcggctc
19681 ttcatttacc aatgccaaca atattagggt tgtatcattt ggtaatggtt accaacagcg
19741 tggcactgga ggatacagga cgaacaagag gacgtatact gttagctacg ttggccgtga
19801 gtgggaggca gtaatgaact tctgccttga tcacatcgta aaacctttcg catggactac
19861 gccacagggt gagttaaaga tgtttgttat tcagcaggat agcattagcg tctcgccgag
19921 cacaaaagaa gttcagactg ttagtatgca atttactgaa gttttcactt caatgagtta
19981 aactaaggcc cataaagggc cttttttat tggagagaat atggcaagtg taaagtttga
20041 aaaccagctt caatcactat ttcctggtga ggtagtcaca cttattgagg ttgatggcag
20101 caagtttggc gcaagggttt acaggtttca tggagaggac atctcataca cgccagagga

20161 aatcatgcaa gcccaaacat ctggtcagcc actaccatcc aagacaatca
cgttccgtgg
20221 cgaagagtat ggagccaggc ctttcggcat tagcggcatc aacctgactt
ctgacggcaa
20281 agcaaacaag ctcacgctaa ttctatcaaa cgttgaccag gctgtttccg
cactcatacg
20341 tcagtataat ggtatggttc aggccaaggt gacaatctgg gttactatca
gggataatat
20401 caatgaggac ggcagcattg cagatggcga ttatcgtaag atggtttact
ttattgagcg
20461 ccctaagcag gttgactaca aaactgcctc ttttgagtta tctagcccaa
ttgatatgga
20521 tggtatttat attccagcca ggctagtcca gtcagtatgc tattgggctt
cccgtggctg
20581 gtaccgcagt gggaatggtt gtgcttatgc tggtaatcga tattttgata
aagacaataa
20641 tcctgttgat gaccctggcc tagatttctg cgccggaact gttgacgcct
gtaagttaag
20701 gtttggcgag aataaccaat tagattttgg cggttgtgcc tcagcaagtt
tgcaagctaa
20761 gaataatcag aactaagggg agtaaaatgc ttacaccaaa agttaaacta
gaaatcatgc
20821 gtcacgcaaa agaggatttc cccaacgaat gctgtggtgt agtcacccag
aaaggcatgg
20881 ctcagaagta ccatcgaatc accaacgcgt ctaaagacac tgagaatgag
ttcattcttg
20941 atgctggcga gtacatgaag attagtgatg aagaacaagt tgtttacatt
gttcactcac
21001 atacgggaga tggcgcgact actcgtccaa gccctgctga tatctgtagc
tgcaatgagt
21061 gcgagattcc ttacgtgatt gttagttacc ctgaggggga catgagaatt
ctcgagcctg
21121 aagaaatgcc gctagcagga aggccgtggg gacttggtag cttcgactgc
tggggcttga
21181 taatgtcctt ccacgcaaaa cacggcgtaa aactgactga ctaccgagtt
aattatccat
21241 ggtggaacaa ggagtacgac gaaaatatct acgacgacaa ttggattaag
gaaggatttg
21301 agctagtcga aactgatgat attcctgctg gctccatgat tatgatgcag
attcaaagcg
21361 agacaacaaa tcacgcagga atctacgttg gagataacaa gttcttgcac
catctttacg
21421 gtaagatgag taaagttgat acctacagtg attactggcg agagcgcacc
gttcgcatcg
21481 ttcgccacaa agatttacca gaaggagcaa gctacaatgc agagactgat
taaagttaaa
21541 ttgggccttt cgcttggtag gaggtatgga aagattcacg aactatgtgt
cgagtctgta
21601 ccggaggcgc ttagggcgtt gtctgtaaac attccagcat tcaaggaatt
catgcagagc
21661 catgtcgggc agaatacaag gtttgcaatc tttgcagatg gcaaaaacct
aaacgagcat
21721 cgcatcaaag actttaaggc cgtaaaggag attcgcatca tgccaatccc
tcaaggacga

21781 aagagtggtg ggttgttcca gactgttttg ggtgcggcgc taattactat tggtgcattc
21841 accggacagg tttatcttgt tgcggcaggt gtttcactcg cagctggcgg cgtagctcaa
21901 ttactgtcac ctcaagcaac aggtcttaaa agccaggagt ctgcacaaaa cagggcttct
21961 tacgcatttg gttcggcggt taacacggtt gctgctggat acccggtatg tctgccatac
22021 ggttatcgaa gtgttggtgg ctccgtattt agtgctggca gttacgctca ggatattgca
22081 taaattaaca cccgcctagt gcgggttttt tatttgatgt acaatggcac caatagtcaa
22141 aaacaaaaca ggataatgtg atatggcgca ggacattaaa atatacggca gcaagggcgg
22201 tagttctaag cagaaacaac cagtagagca gccagataac ttgatatctg taaacaaggt
22261 taaggttttg ctagctgttt ctgatggtga agttgataac tcgttctcgc ttaaagacct
22321 gtatcttgct gacgttccgg ttcagaacca gaatggcaca tacaactacg agggtgtgac
22381 agcagaattt cgccctggca cgcagagtca ggattacatc gccggcctcg agggtgccag
22441 ctctgaggtg caggtcaata gggagattac aaactcaacg ccttacataa ttgccgttaa
22501 caacactcaa ctatctgcga ttagggttaa attatactgg cctcgactga ttaatcagga
22561 aagtaacgga gacctgaacg gaacaacttg cgagtacgct attgacttat ctgttgatgg
22621 ctccacgtac agtgagtaca cgcgaggcgt agctaacggc aagacgacta gcgggtacga
22681 caggagtatc agggtaaacc ttccagctga gttttctagc gctcttgtta gggtccgtaa
22741 aattacagca gactcagaaa gctctactct ggttaacgga atgcaaatta cgacctatca
22801 ggaagtaatc gatgctaaat tccgttatcc gcttacagcg ctggtttacg ttgagtttag
22861 ttcggacctc ttccctaacg gaatcccggc catctcaatc aaaaagaagt ggaagttgat
22921 tcgcgttccg actaactata acccagagac aagaacttat agcggaactt ggaacggcac
22981 atttaagatg gcgtattcaa ataacccagc gtgggttctt tacgaccttg taactaacca
23041 gcgatatggg cttgaccaac gcgaacttgg tatcacaatt gataaatggg ggttgtatga
23101 agcggcgcaa ttctgtgacc aaatggtacc agacggaaag ggaggtgtgg agccgcgtta
23161 cttgtgtgat gttgtaattc agcaaaaggt tgaggcatac caagtcatcc gtgatatctg
23221 ttctatcttc cgtgggctaa cattctatga tggtgagcag atagggattg ttgtcgataa
23281 gccgcgccaa ccatcatacg tattcaccaa cgataacgtt gttgacgggt tatttaacag
23341 gactttcgga agcgacaaat cgctatacac agtcgctaac gttcagtttg atgatgttga

23401 aaactcatac cagcaagatg tagagccagt atttgagcta gaggcaacaa
ggcgttttgg
23461 ttacaacccg gttgacctta ccgctattgg ctgtactcgc aggagtgaag
caaaccgccg
23521 tggtcgttgg ttgttaaaga caaacttgcg cagcgagacg ataagtttca
ccacagggct
23581 agaaggtatg atcccaatga ttggtgaggt gattgcggtt aatgaccagg
catggtctag
23641 caactacacc ttaaaccttt ctggtcgtat cgagtctgta agtggtttgc
aggttttcgt
23701 gccgtttaag attgatgctg ctgccggcga caggattctg attaacaagc
ctgacggaat
23761 gccggaatac aggactattg catctgtatc tgaagatgga atgacgctgc
aacttaacac
23821 ggcgtttagc ttcaaccctc aacctgatac tgtatttgcg attgataagg
agaatcttgc
23881 gcttcagcaa tatgttgtga ccgggattca aaaagcagac agcgatggag
atgatgcgtt
23941 cggttacagt attacagcgg ttgagtacga cccgaacaaa tacgacgaga
ttgattacgg
24001 cgtaaacatt caggacaggc caacgtcaat cgttgagcca gatacattac
tggcacctga
24061 gaatgttcag gtaagcagct acagtaaggt tgtgcagggt atttcagttg
agactatggt
24121 catcacctgg gataaggttc agtacgctaa gacatacaca gttcagtggc
gtaaggagaa
24181 tggaaactgg attaatgtac cgcgcactgc aaacacagaa gttgatattg
aaggtattta
24241 cgcaggcatt tatcaggttc gtgtaagggc tgtttctgat actgaaaacg
tatctgcgtg
24301 gtctgaaatt gtttcagcta gccttacagg taagattgga actccttctg
cacctacagt
24361 tattacggct tcagatgacg aggtgtttgg cattatggtt aagtggggat
tccctgacaa
24421 ctcaactgat acgtcataca ctgaattgca gcaagttgcc gacaatggtg
atggcactta
24481 cacgccagag aacgcaagct tgctaacatt agttccttac ccgagctacg
agtattggca
24541 cactacgcta ccggcaggta aggttatctg gtacagggcg cgactaatcg
acaagattgg
24601 taacgtctca ccatggactg attttgtaat gggcatggca agcgatgacg
tttcggcaat
24661 cattggtgaa attaaggttg atatagagaa ctcggagggg ttcaagtatc
ttcagcagaa
24721 tgccattgag tcaaacggag ctattcaggc gcaagcagaa tcaatcatcg
agaacaccct
24781 ggcgaatgat ggtgacgttc gtcgaatgac taaggaaaac agcaagagga
aggctgaatt
24841 tattcaggca gtcaatctta tcgctgatga agaaagggct cgcgtagagg
ccctgactca
24901 gcttaaggca cagattgatg atgagattgt agcttcgtta acaaggattg
atactgcgct
24961 agctgaggaa actcaagcaa ggaccactgc ggacactgct ttatccgcaa
ggcttggcga

25021 gaatgaagct gcattaaatg agaagcttga cacatacgtt accgctgatt caattggcgc
25081 tcagtatgga attaagctcg gtcttactta caatggagtt cagtacagtt ctgggatgag
25141 catggagcta acaggttctg atggtaatgt tcgtagccag gtcatctttg acgctaacag
25201 gttcgcgatc agtaatggtg ttagttccgg ttccggtcag tggtcattgc ctttcgtagt
25261 tgagaataat caggtcttca ttcagagcgc agtaattcag gacggttcaa tcactaacgc
25321 gaagattggt aacaggattc aatctaacaa ctacgtggct ggatctcaag gatgggcgat
25381 tgacaaggca ggtagcgccg agtttagtaa cgcaactgta agggggaatt tatacgcaaa
25441 taacggtaac tttgcattta acggaacaaa caacactgta cagattaaca acaacggaat
25501 tacagttaat ttaccgaatg gtggcagggt tgtagttgga gtgtggtaaa tgattaggcc
25561 ccttttgggg ccttttcttt tagtagaagt ttgggataaa tggcagagcc attgatatgt
25621 ttgtgtcgaa tgggaactgc gccccagtct gtgttgaata gttccctatt acagttccag
25681 gtacagccat tacggcgcct ccagacatac agacaccctt gtaccttaag ttgttatacc
25741 caccagtaac ccttgttgta gctccacacc tgagtagtgg aaagaacccc ccatttattg
25801 actggaaacc attggttatt tgtataaaac cactaagaac gaaaggtctc ctgacgcttg
25861 agaagacaat ctgaccagaa ctgttagaca ttgtaaggcc gtggccagct attggaggct
25921 catttctgaa tatcactaat tggacgtaag tgcttgctgc cacgtcatct atgccgccgt
25981 aatttatatc gcgacaccat attgtactcc cgtcactctc gagagagacg tttgggttat
26041 cccatttacc aaacgggatt ccgctaactg gaagtgaagc cgcgccgtta acctgaatcc
26101 caccaaccca tgccgccgac attaacctag aattgcttga tattgccaaa aagtcagttg
26161 agttctcaac cagtatgcca gggcttgacg acgatgaagc atcaagtatc tcaaagcaat
26221 taccagcgaa gtcaataatt cgacctctgg caccattaaa gttactaagc gttatcctgc
26281 caactccgtc atttaccttc tgaaacccgt taatataata aacttcaggt acaacatcat
26341 aggcgtcaac gtggacggcc tttgttggca agcagaatgc ggtagagcct ggtgacatgg
26401 ttaaaccaaa gtcccatgat gatgctggcg tcgaaagtcc attagtattt attgatccag
26461 tcacagcagg tgctcttagt ccagcggtaa tctccatcgc tggccttccg tcatttaggt
26521 caatcaatat accttgcggc attatttcca ctccttagat ccgtggtcag caactgaacc
26581 accgtaaccg ccgccaacct tgctctcaat accagcgtag cgcacatcaa cttcaccagc

26641 cggtacaact acaccatttt tccatttagc aacgcacacg ccgtttgctt gcgttgcaca
26701 ccaaccgtca ggacgaggag cagatgaaca acctaccatc atgattgctg ctgcggcgat
26761 gattgaaagt ttgattaagt tagtcattta cgtattcctt atgaggttcg tttcttttcg
26821 atggagtaac tatatctcat tcttgagatt tgtctttagc tattcgtgct gtttattcta
26881 ttttttgcga cattaaaata ctcagtatct agctctatac ctataaatga cctgtttgtg
26941 tttacgcatg ctacgccagt tgttccgcta cccatgcagt tatcaagaac taagtcaccc
27001 tcgtcagtgt atgtttttat taggtactcc atcaaggaaa ccggcttttg cgttgggtgt
27061 agtgatgatt tctgcttgtc gctagccatc tttatcacat cccttgggta tctcattgtt
27121 gaatcataag atagtgattc gaaattctga tctccatagt ttgttccatt gtcgttcctc
27181 tttgttgctg tttttctttt gtgaccctct gtcatttgcg ggttgtattt gtaaggtttt
27241 ttgtagaaaa caagtatgtt ttcatgagcc ttcatcggag ctctcttggc attaaggtgt
27301 ccggttgcat gtgtttttttc ccatatccac tcatgcttca acatttcaag attggagcac
27361 ccgagtactt tatcgaatgg tgtttgcgcc gtaagtacaa ttgcaccctt gcacaccctc
27421 ctgtattgag tccatagcaa gtcaaggtct ataactgaat cccacttgtt tctggttgtt
27481 ccgtatggta agtcgcacaa aatcatgtca attgaattgt ctgggatttc tttcataagc
27541 tcaaggcagt cgccattcat taatttaatc atttccatct ctcattgcgt ttttgttgtg
27601 atgattatac ataaccaagc aagggtgtct ttagctattc gtgctactgg ttacgcgtaa
27661 ccgatgtaaa tcgatgtata ccatatgcat ccgctggaaa tcgtatacgc attgttacgc
27721 ctagttacgc catagttaca ttaaaatatc tatatatatc aacctttttac tcttattatt
27781 attattatat atattactgt gtatatcatg taaccactgt tctggagtgt tccggctata
27841 atttttatat catgtgattt tatacagtat ttcgcggcac atataccaat gcaccacccg
27901 ctaaaatcgg tatacacggt atacgcatta tttacattga gctatagcgg cccagccgga
27961 gcagttaagc gtaactaatg ccaatgtcta cacgtaatta caaaattata cgcgtatact
28021 tgattaatta atcggggata ccaattagac gtggtgacaa gcgttctgca taattatcac
28081 atcgaaagca aacaaccata gagagataga gaaatgccat acttcatcat taaagagaaa
28141 aaaaacgcac tgttcggaga ttgctacttc ggcggtgagg ctggatatac aattcacaaa
28201 tctgaagcgg accgattcga taccaaggaa gaggccaagt ctattctgac tggatatttc

28261 actgaggaag atttgaaagc tgttaagatt gttaaaatta aggattgaac
catgacattt
28321 accgtataca ccaacgacga actaacaaac gaatcctatc acgaagagca
gagccacatc
28381 tctggctctt cactggctga gattctcgcc acttcaccag caaaatggaa
attcaagcag
28441 cgcgataact caagcgcagc acttaagttt ggcactctaa gtcactgcat
gatgcttgag
28501 cgtgaggcgt tcgattcaac ctacatgcga gccactgact ttgatgaagt
tgaggggtta
28561 atcacaagcc aaaccgcgtt atcggcagca cttaagaaag ttggagtttc
tggtacgtca
28621 ggaaaaggct ataacgagct agtagagatg atgtacaagt gcggtgagga
ttggcctgtt
28681 aagtggctaa ttgaacagca ggagagcgct gaggcgttaa ttagtggcaa
ggagttggtt
28741 agtgcgaaag attacgataa ggtaatcgca atgcgcgagg ttctgaccaa
tatcccagct
28801 tacgatgcag ttattaattc accaacagcg cagaaagagc tatcaatctt
tggtgagatt
28861 cttggatgcg gcgttaaggt tagacttgac catgtggatg ttgttgatgg
cgcggtttac
28921 attactgact acaaaaccac agcagacgca agtcctcgag gatttggtaa
gtcggcttat
28981 gaccatggat acctttgcaa gatggcactc cagcgcgatt tgtttgttaa
ggcgtttagc
29041 gagaagcgta aggttgtagt tcgtcttctg gcgcaggaga agacagagcc
ttatttgcca
29101 atgtgctaca cgctcacaga tgagcagctt cacattggtc gccttcaata
tatggaagca
29161 cttgccacgt acaagcagtg taaggaactt gatgtttggc ctggatacag
taatgggatt
29221 acagagcaag atttaatgat tcctgaatgg gtagcaaaac aatacggaga
gatattaaaa
29281 tgaataaaga ttgctggcag acaccattag aattatttaa tcaactaaat
gataagtaca
29341 atttccaggt tgacgcgtgc gcaagtcaag agaatgcttt gtgtgagcat
ttttggaccg
29401 aagatgatga ctgtttgcag caggattggg gtgatgaaaa tgattttgtt
tggtgcaacc
29461 caccatacag caacccattg ccatttgtcg aaaagtgcca ggagtacggt
tacgctgtta
29521 tgcttttaaa tcaggacaca tcaacgaaat gggctaagat agtttttgaa
aaatcctcaa
29581 aggtaatact gttatcagat agggttaggt ttataaaccc atcgacgctc
gagattggga
29641 agtcaaaccc aaaatgccag atgattgtag tgttcgataa tagtgtggag
catgggcctg
29701 tcataaggtg gtgctcaata aaagatattc acaaaaacat ctaaatagca
ctaattgcta
29761 aagataaaat taacaggtgt ggtatgatta ccacatcaac aacgagatgg
tttaagcaaa
29821 tgaaagtatt taaaagtaaa atatgtagct acattaaaat ggccattaat
cgaaacgatg

29881 atattataaa aacaatagat ataaaacctg gcgacgaggt ttttgtcata atcgaaaagt
29941 ctatagcgtt agaacttgca taatacatct taaaaaattc agaaaaacta gaggattgat
30001 atgaacattt cagaatcatg caaatcaatt ttaaacgcac tgcacactgc taaatcgcta
30061 tttgcgaagg ctgagaagtc aaagcaaaac tcgcacttaa agaacaagta cgccacttta
30121 gaagacgttt tagcagcagt tgagccagga ttggatgagt gcggtttggt aatgttccaa
30181 agcgtaatag atgatgaatc aacgaaccga ataaaggttg aaactaagct gtttcatgca
30241 gaatctggag aatgggttag cttcttaatg attgtcccaa tcagtaagca agatgctcaa
30301 ggttacggct ctgctcttac atatgctcga cgctatggca ttacagcagc cttagggctt
30361 agccaggctg atgatgatgg taatcttgca gctaagggtg ttaaagactt caagcgagaa
30421 cttgagaagt gcaatacgct cgatgagctt cgtaatgtct ggaaggaggc taaacagtca
30481 cttgatgcgg caggatggaa ggttttcgaa ccacacatta tcgagcgcaa ggcagagatt
30541 caggcaaatg aaatgaatgg cttcaaccca gccacgccta agaaagttgc agaaaaaagt
30601 agtgataaag aaaagattga agtagaatca caacaaatcg atacattcta aattaaacgg
30661 ggcttcggcc ccagcaaaca gagagataaa aacatgcacg taattactgg tcagattcgc
30721 cgcgctccat tcactaaaga aggtcagaat gctaatggta cctggaaaat gtttgcggtt
30781 gatttgagtg agcgatacaa agataaagat ggtcagactc agtatagtaa ctatcgcgca
30841 gtcttcttcg cgaaagagtc aattttagat tggtataatg aagcatttca agaaggtaaa
30901 attgttagcg ttagcgctga aagtttatct gtagatgttc gagataaaga tggcaagact
30961 tacgtaacaa tcaatatgaa taatcctcga cttgagttta gtcagcgtgg agattcgcaa
31021 ccacaacaaa atcagagaca gcagcagcaa caacagcagc agaataatca gcagaagagg
31081 caaccacaac cacaacaaca gcagcagttt gacgactcca ttccattctg ataaaagata
31141 aggccccttt cggggccttt ttatggcatg tttactcgaa ttgaaacgaa cgagtcagaa
31201 ggtatatcta tagggtctcc gttactgaag ccgtcaattt tatttctcgc gaaagacgga
31261 gatgaatcgt gagtgcgatg ataagtttta acaactattg aaccatcagc ttcaacctcg
31321 taatctagcc atattctagg ctgcttattc aagtctacag gtacctcaaa tccaccgtta
31381 acaccgcccc aagaagtgtc agaatttagg ccaagcacac ctgatacctt ataaacacca
31441 gtagaaaccc tctccactga agctccttct gattcctcgt ttgtttcaaa actcccatct

31501 cggaatacct taattatagg tgaggccact ttgacgaaac cgctagggtc cactgttgtg
31561 tttaatccgt gtaaaagttc cattaccgca agtccgccat tataagttga gttagctata
31621 taacaacgcc ttgccccata agatatggca agctgacccc actcgcgtgg agttgggcca
31681 ggcatatcca tgaatacaac agaatctttg tgtaagttaa aaccggaacc gtaaggctca
31741 tatccataag accatgtttt tgttgtccta ttcgctgaaa gaaaccttgg tccataaact
31801 agctggacct gccctgcatc gtctccatga ttaatagtcg cagaggttcc aagtccaaga
31861 ttggaccttg cttgtagctt gttttgtaag tcattcaggt tttcttcttt ttttaaaaaa
31921 cttgatggat ttactgaatt agcgtaatct gccgcttgct gcgctgatgc tcagggagca
31981 atccttcttc agttgctcaa gcgcttcgcc tttaatcttc atttttaaac cacctttctt
32041 taagaacctg agcccaagac tttgcatcat cttctgtcat gaagaaactt aattttgggt
32101 acttgtgaaa cccctttaac tcaacaaccc aatgaccatt attgtgactg taagaaactg
32161 ttttttcttt cattattacc atctctctat atttgcttgt tatcgaaatc tcttcaacgt
32221 actcgctaca actcgggatt ttaaacccgg cccggcatcc tggcaaaagg acccgcaagg
32281 cgctttggtg ggttaggtaa cgcttcctca cattgcgcgg tgtcgttcac tctttttggt
32341 tcctttcgga agtgactcag agtccagcac cgtttcgatg aatacatagt agcaacttac
32401 tcatgattga gcattagcaa aaagtgctat tcgttggatt tattgattgt tgcagctatt
32461 gcaatgattc acatgactat tgcaca
//
